# On the directional asymptotic approach in optimization theory

**DOI:** 10.1007/s10107-024-02089-w

**Published:** 2024-07-05

**Authors:** Matúš Benko, Patrick Mehlitz

**Affiliations:** 1 Applied Mathematics and Optimization, University of Vienna, 1090 Vienna, Austria; 2 Johann Radon Institute for Computational and Applied Mathematics, 4040 Linz, Austria; 3 Department of Mathematics and Computer Science, Philipps-Universität Marburg, 35032 Marburg, Germany

**Keywords:** Asymptotic stationarity and regularity, Constraint qualifications, Directional limiting variational calculus, M-stationarity, Pseudo- and super-coderivatives, Pseudo- and quasi-normality, 49J52, 49J53, 49K27, 90C22, 90C30, 90C33

## Abstract

As a starting point of our research, we show that, for a fixed order $\gamma \ge 1$, each local minimizer of a rather general nonsmooth optimization problem in Euclidean spaces is either M-stationary in the classical sense (corresponding to stationarity of order 1), satisfies stationarity conditions in terms of a coderivative construction of order $\gamma$, or is asymptotically stationary with respect to a critical direction as well as order $\gamma$ in a certain sense. By ruling out the latter case with a constraint qualification not stronger than directional metric subregularity, we end up with new necessary optimality conditions comprising a mixture of limiting variational tools of orders 1 and $\gamma$. These abstract findings are carved out for the broad class of geometric constraints and $\gamma :=2$, and visualized by examples from complementarity-constrained and nonlinear semidefinite optimization. As a byproduct of the particular setting $\gamma :=1$, our general approach yields new so-called directional asymptotic regularity conditions which serve as constraint qualifications guaranteeing M-stationarity of local minimizers. We compare these new regularity conditions with standard constraint qualifications from nonsmooth optimization. Further, we extend directional concepts of pseudo- and quasi-normality to arbitrary set-valued mappings. It is shown that these properties provide sufficient conditions for the validity of directional asymptotic regularity. Finally, a novel coderivative-like variational tool is used to construct sufficient conditions for the presence of directional asymptotic regularity. For geometric constraints, it is illustrated that all appearing objects can be calculated in terms of initial problem data.

## Introduction

In order to identify local minimizers of optimization problems analytically or numerically, it is desirable that such points satisfy applicable necessary optimality conditions. Typically, under validity of a constraint qualification, first-order necessary optimality conditions of abstract Karush–Kuhn–Tucker (KKT)-type hold at local minimizers. Here, *first-order* refers to the fact that first-order tools of (generalized) differentiation are used to describe the variation of all involved data functions. In the case where the celebrated tools of limiting variational analysis are exploited, one speaks of so-called Mordukhovich (or, briefly, M-) stationarity, see [66]. In the absence of constraint qualifications, i.e., in a *degenerate* situation, local minimizers still satisfy a Fritz–John (FJ)-type first-order necessary optimality condition which allows for a potentially vanishing multiplier associated with the generalized derivative of the objective function. Since such a condition allows to discard the objective function, it might be too weak in practically relevant scenarios.

In recent years, *asymptotic* (*approximate* or *sequential* are also common) concepts of stationarity and regularity received much attention not only in standard nonlinear optimization, see [3, 5–7], but also in complementarity-, cardinality-, and switching-constrained programming, see [4, 53, 61, 70], conic optimization, see [2], nonsmooth optimization, see [45, 63, 64], or even infinite-dimensional optimization, see [26, 55, 58]. The interest in asymptotic stationarity conditions is based on the observation that they hold at local minimizers in the absence of constraint qualifications while being more restrictive than the corresponding FJ-type conditions, and that different types of solution algorithms like multiplier-penalty- and some SQP-methods naturally compute such points. Asymptotic constraint qualifications provide conditions which guarantee that an asymptotically stationary point is already stationary in classical sense. It has been reported, e.g., in [5, 61, 63, 70] that asymptotic constraint qualifications are comparatively mild. Inherently from their construction, asymptotic constraint qualifications simplify the convergence analysis of some numerical solution algorithms.

The aim of this paper is to apply the *directional* approach to limiting variational analysis, see e.g. [18], in order to enrich the asymptotic stationarity and regularity conditions from [58, 63] with the aid of directional information. Noting that the directional tools of variational analysis were successfully applied to find refined M-stationarity-type optimality conditions and mild constraint qualifications for diverse problems in optimization theory, see e.g. [14–16, 36, 38, 39, 42] and the references therein, this seems to be a desirable goal.

Section 4 contains the core of our research. As a starting point, we show in Sect. 4.2 (see, particularly, Theorem 4.1) that local minimizers of rather general optimization problems in Euclidean spaces, which we formally introduce in Sect. 4, are either M-stationary, satisfy a stationarity condition combining the limiting subdifferential of the objective function and a coderivative-like tool associated with the constraints of some arbitrary order $\gamma \ge 1$, a so-called *pseudo-coderivative*, see [37], or come along with an asymptotic stationarity condition depending on a critical direction as well as the order $\gamma$ where the involved sequence of multipliers is diverging. Even for $\gamma :=1$, this enhances the findings from [58, 63]. Furthermore, this result opens a new way on how to come up with applicable necessary optimality conditions for the original problem, namely, by ruling out the irregular situation of asymptotic stationarity which can be done in the presence of so-called *metric pseudo-subregularity* of order $\gamma$, see [37] again. In the case $\gamma :=1$, we end up with M-stationarity, and metric pseudo-subregularity reduces to metric subregularity, i.e., we obtain results related to [36]. For $\gamma >1$, this procedure leads to a mixed-order stationarity condition involving the pseudo-coderivative of order $\gamma$, and metric pseudo-subregularity is weaker than metric subregularity. If $\gamma :=2$ and so-called geometric constraints, induced by a twice continuously differentiable mapping *g* as well as a closed set *D*, are investigated, this pseudo-coderivative can be estimated from above in terms of initial problem data, i.e., in terms of (first- and second-order) derivatives associated with *g* as well as tangent and normal cones to *D*, under mild conditions. These estimates of the pseudo-coderivative of order 2 are interesting on their own and presented in Sect. 3, which is the essence to all applications of our general findings. The associated mixed-order necessary optimality conditions and qualification conditions are worked out in Sect. 4.3, and in Sect. 4.4, they are applied to complementarity-constrained and nonlinear semidefinite optimization problems in order to illustrate our findings. Let us note that related necessary optimality conditions for optimization problems which comprise first- and second-order tools at the same time can be found e.g. in [9, 11–13, 35, 37, 51]. These results are based on the concept of 2–regularity and its extensions, see [11, 74] for its origins. Indeed, even Gfrerer’s metric pseudo-subregularity from [37], utilized in this paper, can be seen as an extension of 2–regularity to arbitrary set-valued mappings. For us, however, these mixed-order conditions are only a by-product - we focus on how they can be used to find new constraint qualifications guaranteeing M-stationarity of local minimizers.

Section 5 is dedicated to the investigation of *directional* asymptotic regularity conditions, which are motivated by the asymptotic stationarity conditions from Theorem 4.1 (for $\gamma :=1$) and whose validity directly yields M-stationarity of local minimizers. Roughly speaking, these conditions demand certain control of unbounded input sequences (multipliers) associated with the regular coderivative of the underlying set-valued mapping in a neighborhood of the reference point. We enrich and refine the asymptotic regularity conditions from [63] in two ways. First, the directional approach reveals that asymptotic regularity is only necessary in critical directions. Second, we observe an additional restriction the problematic multipliers satisfy: while their norm tends to infinity, their direction is tightly controlled. These insights enable us to relate our new constraint qualifications with already existing ones from the literature. Similarly as standard asymptotic regularity, the directional counterpart is also independent of (directional) metric subregularity. However, several sufficient conditions for metric subregularity, which are independent of asymptotic regularity, imply directional asymptotic regularity. For instance, this is true for the First-Order Sufficient Condition for Metric Subregularity from [39], see Sect. 5.1. Moreover, in Sect. 5.2, we extend the (directional) concepts of pseudo- and quasi-normality from [15, 16] to abstract set-valued mappings and show that these conditions are sufficient for directional metric subregularity as well as directional asymptotic regularity. Notably, even standard (nondirectional) versions of pseudo- and quasi-normality do not imply asymptotic regularity since the latter does not restrict the direction of the problematic multipliers. Finally, a new directional coderivative-like tool, the *directional super-coderivative*, see Sect. 2.3, is used in Sect. 5.3 to construct sufficient conditions for the validity of directional asymptotic regularity. In the presence of so-called metric pseudo-regularity, see [37] again, this leads to conditions in terms of the aforementioned pseudo-coderivatives. Noting that these generalized derivatives can be computed in terms of initial problem data for geometric constraint systems, we can specify our findings in this situation. As it turns out, the approach is closely related to our findings from Sect. 4.3. Furthermore, we show that the explicit sufficient conditions for directional asymptotic regularity provide constraint qualifications for M-stationarity which are not stronger than the First- and Second-Order Sufficient Condition for Metric Subregularity from [39].

## Notation and preliminaries

We rely on standard notation taken from [10, 25, 66, 71].

### Basic notation

Let ${\mathbb {R}}$, ${\mathbb {R}}_+$, and ${\mathbb {R}}_-$ denote the real, the nonnegative real, and the nonpositive real numbers, respectively. The sign function ${\text {sgn}}:{\mathbb {R}}\rightarrow \{-1,0,1\}$ is defined by ${\text {sgn}}(t):=-1$ for all $t<0$, ${\text {sgn}}(t):=1$ for all $t>0$, and ${\text {sgn}}(0):=0$. Throughout the paper, ${\mathbb {X}}$ and ${\mathbb {Y}}$ denote Euclidean spaces, i.e., finite-dimensional Hilbert spaces. For simplicity, the associated inner product will be represented by $\langle \cdot , \cdot \rangle$ since the underlying space will be clear from the context. The norm induced by the inner product is denoted by $\left\| \cdot \right\|$. The unit sphere in ${\mathbb {X}}$ will be represented by ${\mathbb {S}}_{\mathbb X}$. Furthermore, for $\varepsilon >0$ and ${\bar{x}}\in {\mathbb {X}}$, ${\mathbb {B}}_\varepsilon ({\bar{x}}):=\{x\in {\mathbb {X}}\,|\!\left\| x-\bar{x}\right\| \le \varepsilon \}$ is the closed $\varepsilon$-ball around $\bar{x}$. We are also concerned with so-called (closed) directional neighborhoods of given directions. These are sets of type

$$\begin{aligned} {\mathbb {B}}_{\varepsilon ,\delta }(u):= \{w\in {\mathbb {X}}\,|\!\left\| \left\| w\right\| u-\left\| u\right\| w\right\| \le \delta \left\| u\right\| \left\| w\right\| ,\,\left\| w\right\| \le \varepsilon \}, \end{aligned}$$

where $u\in {\mathbb {X}}$ is a reference direction and $\varepsilon ,\delta >0$. Clearly, ${\mathbb {B}}_{\varepsilon ,\delta }(0)={\mathbb {B}}_{\varepsilon }(0)$. For a nonempty set $Q\!\!\subset \!{\mathbb {X}}$, the closed convex cone $Q^\circ :=\!\!\{\eta \in {\mathbb {X}}|\forall x\in Q:\langle \eta , x\rangle \le 0\}$ is referred to as the polar cone of *Q*. Furthermore, for some ${\bar{x}}\in {\mathbb {X}}$, $[{\bar{x}}]^\perp :=\{\eta \in {\mathbb {X}}\,|\,\langle \eta , {\bar{x}}\rangle =0\}$ and ${\text {span}}({\bar{x}})$ are the annihilator of ${\bar{x}}$ and the smallest subspace of ${\mathbb {X}}$ containing ${\bar{x}}$, respectively. By ${\text {dist}}({\bar{x}},Q):=\inf _x\{\left\| x-{\bar{x}}\right\| \,|\,x\in Q\}$, we denote the distance of ${\bar{x}}$ to *Q*. For simplicity, we use ${\bar{x}}+Q:=Q+{\bar{x}}:=\{x+{\bar{x}}\in {\mathbb {X}}\,|\,x\in Q\}$. The closure and the horizon cone of *Q* are represented by ${\text {cl}}(Q)$ and $Q^\infty$, respectively. For a given linear operator $A:{\mathbb {X}}\rightarrow {\mathbb {Y}}$, $A^*:{\mathbb {Y}}\rightarrow {\mathbb {X}}$ is used to denote its adjoint while ${\text {Im}}A:=\{Ax\in {\mathbb {Y}}\,|\,x\in {\mathbb {X}}\}$ is the image of *A*.

Let $g:{\mathbb {X}}\rightarrow {\mathbb {Y}}$ be a continuously differentiable mapping. We use $\nabla g({\bar{x}}):{\mathbb {X}}\rightarrow {\mathbb {Y}}$ to denote the derivative of *g* at ${\bar{x}}\in {\mathbb {X}}$. Note that $\nabla g({\bar{x}})$ is a linear operator. Let us emphasize that, in the special case ${\mathbb {Y}}:={\mathbb {R}}$, $\nabla g({\bar{x}})$ does not coincide with the standard gradient which would correspond to $\nabla g({\bar{x}})^*1$. For twice continuously differentiable *g* and a vector $\lambda \in {\mathbb {Y}}$, we set $\langle \lambda ,g\rangle (x):=\langle \lambda , g(x) \rangle$ for each $x\in {\mathbb {X}}$ in order to denote the associated scalarization mapping $\langle \lambda , g\rangle :{\mathbb {X}}\rightarrow {\mathbb {R}}$. By $\nabla \langle \lambda , g\rangle ({\bar{x}})$ and $\nabla ^2\langle \lambda , g\rangle ({\bar{x}})$ we represent the first- and second-order derivatives of this map at ${\bar{x}}\in {\mathbb {X}}$ (w.r.t. the variable which enters *g*). Furthermore, for $u,u'\in {\mathbb {X}}$, we make use of

$$\begin{aligned} \nabla ^2g({\bar{x}})[u,u']:= \sum _{i=1}^m \langle u, \nabla ^2\langle e_i^c , g\rangle ({\bar{x}})(u')\rangle \,e_i^c \end{aligned}$$

for brevity where $m\in {\mathbb {N}}$ is the dimension of ${\mathbb {Y}}$ and $e_1^c ,\ldots ,e_m^c \in {\mathbb {Y}}$ denote the *m* canonical unit vectors of ${\mathbb {Y}}$. In the case ${\mathbb {Y}}:={\mathbb {R}}$, the second-order derivative $\nabla ^2\,g({\bar{x}}):{\mathbb {X}}\times {\mathbb {X}}\rightarrow {\mathbb {R}}$ is a bilinear mapping, and for each $u\in {\mathbb {X}}$, we identify $\nabla ^2g({\bar{x}})u$ with an element of ${\mathbb {X}}$.

### Fundamentals of variational analysis

Let us fix a closed set $Q\subset {\mathbb {X}}$ and some point $x\in Q$. We use

$$\begin{aligned} {\mathcal {T}}_Q(x) := \left\{ u\in {\mathbb {X}}\,\left| \, \begin{aligned}&\exists \{u_k\}_{k\in {\mathbb {N}}}\subset {\mathbb {X}},\,\exists \{t_k\}_{k\in {\mathbb {N}}}\subset {\mathbb {R}}_+:\\&\qquad u_k\rightarrow u,\,t_k\downarrow 0,\,x+t_ku_k\in Q\,\forall k\in {\mathbb {N}}\end{aligned}\right. \right\} \end{aligned}$$

to denote the (Bouligand) *tangent cone* to *Q* at *x*. Furthermore, we make use ofthe *regular* (or Fréchet) and *limiting* (or Mordukhovich) *normal cone* to *Q* at *x*. Observe that both of these normal cones coincide with the standard normal cone of convex analysis as soon as *Q* is convex. For ${{\tilde{x}}}\notin Q$, we set ${\mathcal {T}}_Q({{\tilde{x}}}):=\emptyset$ and $\widehat{{\mathcal {N}}}_Q({{\tilde{x}}}):={\mathcal {N}}_Q({{\tilde{x}}}):=\emptyset$. Finally, for some $u\in {\mathbb {X}}$, we use

$$\begin{aligned} {\mathcal {N}}_Q(x;u):= \left\{ \eta \in {\mathbb {X}}\,\left| \, \begin{aligned}&\exists \{u_k\}_{k\in {\mathbb {N}}}\subset {\mathbb {X}},\,\exists \{t_k\}_{k\in {\mathbb {N}}}\subset {\mathbb {R}}_+,\, \exists \{\eta _k\}_{k\in {\mathbb {N}}}\subset {\mathbb {X}}:\\&\qquad u_k\rightarrow u,\,t_k\downarrow 0,\,\eta _k\rightarrow \eta ,\,\eta _k\in \widehat{{\mathcal {N}}}_Q(x+t_ku_k)\,\forall k\in {\mathbb {N}}\end{aligned}\right. \right\} \end{aligned}$$

in order to represent the *directional limiting normal cone* to *Q* at *x* in direction *u*. Note that this set is empty if *u* does not belong to ${\mathcal {T}}_Q(x)$. If *Q* is convex, we have ${\mathcal {N}}_Q(x;u)={\mathcal {N}}_Q(x)\cap [u]^\perp$.

The limiting normal cone to a set is well known for its *robustness*, i.e., it is outer semicontinuous as a set-valued mapping. In the course of the paper, we exploit an analogous property of the directional limiting normal cone which has been validated in [42, Proposition 2].

#### Lemma 2.1

Let $Q\subset {\mathbb {X}}$ be closed and fix $x\in Q$. Then, for each $u\in {\mathbb {X}}$, we have

$$\begin{aligned} {\mathcal {N}}_Q(x;u) = \left\{ \eta \in {\mathbb {X}}\,\left| \, \begin{aligned}&\exists \{u_k\}_{k\in {\mathbb {N}}}\subset {\mathbb {X}},\, \exists \{t_k\}_{k\in {\mathbb {N}}}\subset {\mathbb {R}}_+,\, \exists \{\eta _k\}_{k\in {\mathbb {N}}}\subset {\mathbb {X}}:\\&\qquad u_k\rightarrow u,\,t_k\downarrow 0,\,\eta _k\rightarrow \eta ,\, \eta _k\in {\mathcal {N}}_Q(x+t_ku_k)\,\forall k\in {\mathbb {N}}\end{aligned}\right. \right\} . \end{aligned}$$

In this paper, the concept of *polyhedrality* will be of essential importance. Let us recall that a set $Q\subset {\mathbb {R}}^m$ will be called polyhedral if it is the union of finitely many convex polyhedral sets. Similarly, it is referred to as locally polyhedral around $x\in Q$ whenever $Q\cap \{z\in {\mathbb {R}}^m\,|\,\forall i\in \{1,\ldots ,m\}:\,|z_i-x_i|\le \varepsilon \}$ is polyhedral for some $\varepsilon >0$. The following lemma provides some basic properties of polyhedral sets. Statement (a) is proven in [48, Proposition 8.24]. The equality in statement (b) follows from [19, Proposition 2.11] and the rest is straightforward, see [38, Lemma 2.1] as well.

#### Lemma 2.2

Let $Q\subset \mathbb {\mathbb {R}}^m$ be a closed set which is locally polyhedral around some fixed point $x\in Q$. Then the following statements hold.

- There exists a neighborhood $U\subset {\mathbb {R}}^m$ of *x* such that $(x+{\mathcal {T}}_Q(x))\cap U=Q\cap U$.
- For arbitrary $u\in {\mathbb {R}}^m$, we have
  2.1 $$\begin{aligned} {\mathcal {N}}_Q(x;u) = {\mathcal {N}}_{{\mathcal {T}}_Q(x)}(u) \subset {\mathcal {N}}_{Q}(x)\cap [u]^\perp . \end{aligned}$$
  If *Q* is, additionally, convex, and $u\in {\mathcal {T}}_Q(x)$, then the final inclusion holds as an equality.

It is well known that the regular and limiting normal cone enjoy an exact product rule which is not true for the tangent cone in general. However, the following lemma shows that such a product rule also holds for tangents as soon as polyhedral sets are under consideration. Its proof is straightforward and, hence, omitted.

#### Lemma 2.3

- For closed sets $P\subset {\mathbb {X}}$ and $Q\subset {\mathbb {Y}}$ as well as $x\in P$ and $y\in Q$, we have ${\mathcal {T}}_{P\times Q}(x,y)\subset {\mathcal {T}}_P(x)\times {\mathcal {T}}_Q(y)$.
- For closed sets $P\subset {\mathbb {R}}^n$ and $Q\subset {\mathbb {R}}^m$ as well as $x\in P$ and $y\in Q$, such that *P* and *Q* are locally polyhedral around *x* and *y*, respectively, we have ${\mathcal {T}}_{P\times Q}(x,y)={\mathcal {T}}_P(x)\times {\mathcal {T}}_Q(y)$.

Let us mention that a slightly more general version of the above lemma can be found in [41, Proposition 1].

For a set-valued mapping $\varPhi :{\mathbb {X}}\rightrightarrows {\mathbb {Y}}$, we use ${\text {dom}}\varPhi :=\{x\in {\mathbb {X}}\,|\,\varPhi (x)\ne \emptyset \}$, ${\text {gph}}\varPhi :=\{(x,y)\in {\mathbb {X}}\times {\mathbb {Y}}\,|\,y\in \varPhi (x)\}$, $\ker \varPhi :=\{x\in {\mathbb {X}}\,|\,0\in \varPhi (x)\}$, and ${\text {Im}}\varPhi :=\bigcup _{x\in {\mathbb {X}}}\varPhi (x)$ in order to represent the domain, graph, kernel, and image of $\varPhi$, respectively. Furthermore, the so-called inverse mapping $\varPhi ^{-1}:{\mathbb {Y}}\rightrightarrows {\mathbb {X}}$ is defined via ${\text {gph}}\varPhi ^{-1}:=\{(y,x)\in {\mathbb {Y}}\times {\mathbb {X}}\,|\,(x,y)\in {\text {gph}}\varPhi \}$.

There exist numerous concepts of local *regularity* or *Lipschitzness* associated with set-valued mappings. In this paper, we are mostly concerned with so-called directional metric pseudo-(sub)regularity which originates from [37, Definition 1].

#### Definition 2.1

Fix a set-valued mapping $\varPhi :{\mathbb {X}}\rightrightarrows {\mathbb {Y}}$ which has a closed graph locally around $({\bar{x}},{\bar{y}})\in {\text {gph}}\varPhi$, a pair of directions $(u,v)\in {\mathbb {X}}\times {\mathbb {Y}}$, and a constant $\gamma \ge 1$.

- We say that $\varPhi$ is *metrically pseudo-regular of order* $\gamma$ *in direction* (*u*, *v*) at $({\bar{x}},{\bar{y}})$ if there are constants $\varepsilon >0$, $\delta >0$, and $\kappa >0$ such that the estimate
  2.2 $$\begin{aligned} \left\| x-{\bar{x}}\right\| ^{\gamma -1}{\text {dist}}(x,\varPhi ^{-1}(y)) \le \kappa \,{\text {dist}}(y,\varPhi (x)) \end{aligned}$$
  holds for all $(x,y)\in ({\bar{x}},{\bar{y}})+{\mathbb {B}}_{\varepsilon ,\delta }(u,v)$ with ${\text {dist}}(y,\varPhi (x))\le \delta \left\| x-{\bar{x}}\right\| ^\gamma$. In the case where this is fulfilled for $(u,v):=(0,0)$, we say that $\varPhi$ is *metrically pseudo-regular of order* $\gamma$ at $({\bar{x}},{\bar{y}})$.
- We say that $\varPhi$ is *metrically pseudo-subregular of order *$\gamma$* in direction u* at $({\bar{x}},{\bar{y}})$ if there are constants $\varepsilon >0$, $\delta >0$, and $\kappa >0$ such that (2.2) holds for $y:={\bar{y}}$ and all $x\in {\bar{x}}+{\mathbb {B}}_{\varepsilon ,\delta }(u)$. In the case where this is fulfilled for $u:=0$, we say that $\varPhi$ is *metrically pseudo-subregular of order* $\gamma$ at $({\bar{x}},{\bar{y}})$.

Metric pseudo-regularity of order $\gamma \ge 1$ in direction (*u*, 0) at $({\bar{x}},{\bar{y}})$ is a sufficient condition for metric pseudo-subregularity of order $\gamma$ in direction *u* at the same point, see [37, Lemma 3]. Observe that metric pseudo-subregularity in a specified direction of some order $\gamma \ge 1$ implies metric pseudo-subregularity of arbitrary order larger than $\gamma$ in the same direction. For $\gamma :=1$, the above definition of (directional) metric pseudo-subregularity recovers the one of *(directional) metric subregularity*, see [36, Definition 1.2]. On the contrary, for $\gamma :=1$, the above definition of directional metric pseudo-regularity does not recover the one of *directional metric regularity* which demands that (2.2) holds for all $(x,y)\in ({\bar{x}},{\bar{y}})+{\mathbb {B}}_{\varepsilon ,\delta }(u,v)$ such that $\left\| (u,v)\right\| {\text {dist}}((x,y),{\text {gph}}\varPhi )\le \delta \left\| (u,v)\right\| \left\| (x,y)-({\bar{x}},{\bar{y}})\right\|$, see [36, Definition 1.1]. Particularly, for $(u,v):=(0,0)$, the notion of directional metric regularity reduces to the classical one of metric regularity, while directional metric pseudo-regularity does not. This was shown in [37, Example 1.1], which is a very natural example, and we will use it to illustrate some novel concepts.

#### Example 2.1

For every $\gamma \ge 1$, the mapping $\varPhi :{\mathbb {R}}\rightrightarrows {\mathbb {R}}$, given by $\varPhi (x):= \{ \vert x \vert ^\gamma \}$, $x\in {\mathbb {R}}$, is metrically pseudo-regular of order $\gamma$ at (0, 0). The case $\gamma := 1$ provides an example of a mapping which is metrically pseudo-regular of order 1 at (0, 0) but not metrically regular there. The violation of metric regularity is clear as any points $y<0$ approaching 0 come along with $\varPhi ^{-1}(y) = \emptyset$, blowing up the left-hand side of (2.2). These problematic elements *y* are, however, ruled out by the condition ${\text {dist}}(y,\varPhi (x))\le \delta \left\| x-{\bar{x}}\right\| ^\gamma$ in the definition of metric pseudo-regularity, which reads $\vert y - \vert x \vert \vert \le \delta \vert x \vert$ in the present situation.

Another important case, which we will explore in detail, corresponds to $\gamma :=2$. In this case, the notions from Definition 2.1 provide an extension of so-called *2-regularity* from [11, 74] to set-valued mappings. In Sect. 3.2, we compare our approach with an extension of 2-regularity to constraint mappings from [8, 9].

Recall that a single-valued function $g:{\mathbb {X}}\rightarrow {\mathbb {Y}}$ is called *calm in direction* $u\in {\mathbb {X}}$ at $x\in {\mathbb {X}}$ whenever there are constants $\varepsilon >0$, $\delta >0$, and $L>0$ such that

$$\begin{aligned} \forall x'\in x+{\mathbb {B}}_{\varepsilon ,\delta }(u):\quad \Vert g(x')-g(x)\Vert \le L\Vert x'-x\Vert . \end{aligned}$$

If this holds for $u:=0$, we simply say that *g* is calm at *x*. Clearly, the latter property is weaker than Lipschitzness of *g* at *x*.

### Generalized differentiation

In this section, we recall some notions from generalized differentiation and introduce some novel derivatives for set-valued mappings.

#### Subdifferentials

Let us start with a lower semicontinuous function $\varphi :{\mathbb {X}}\rightarrow {\mathbb {R}}\cup \{\infty \}$ and some point ${\bar{x}}\in {\text {dom}}\varphi :=\{x\in {\mathbb {X}}\,|\,\varphi (x)<\infty \}$. The lower semicontinuous function $\textrm{d}\varphi ({\bar{x}}):{\mathbb {X}}\rightarrow {\mathbb {R}}\cup \{-\infty ,\infty \}$ given by

$$\begin{aligned} \forall u\in {\mathbb {X}}:\quad \textrm{d}\varphi ({\bar{x}})(u):= \liminf \limits _{t\downarrow 0,\,u'\rightarrow u} \frac{\varphi ({\bar{x}}+tu')-\varphi ({\bar{x}})}{t} \end{aligned}$$

is referred to as the *subderivative* of $\varphi$ at ${\bar{x}}$. The *regular* (or Fréchet) and *limiting* (or Mordukhovich) *subdifferential* of $\varphi$ at ${\bar{x}}$ are given by

$$\begin{aligned} {\widehat{\partial }} \varphi ({\bar{x}})&:= \{ \eta \in {\mathbb {X}}\,|\, (\eta ,-1)\in \widehat{{\mathcal {N}}}_{{\text {epi}}\varphi }({\bar{x}},\varphi ({\bar{x}})) \},\\ \partial \varphi ({\bar{x}})&:= \{ \eta \in {\mathbb {X}}\,|\, (\eta ,-1)\in {\mathcal {N}}_{{\text {epi}}\varphi }({\bar{x}},\varphi ({\bar{x}})) \}, \end{aligned}$$

respectively, where ${\text {epi}}\varphi :=\{(x,\alpha )\in {\mathbb {X}}\times {\mathbb {R}}\,|\,\varphi (x)\le \alpha \}$ is the epigraph of $\varphi$. In the case where $\varphi$ is continuously differentiable at ${\bar{x}}$, both sets reduce to the singleton containing only the gradient $\nabla \varphi ({\bar{x}})^*1$. We note that for any sequences $\{x_k\}_{k\in {\mathbb {N}}}\subset {\text {dom}}\varphi$ and $\{x_k^*\}_{k\in {\mathbb {N}}}\subset {\mathbb {X}}$ such that $x_k\rightarrow {\bar{x}}$, $\varphi (x_k)\rightarrow \varphi ({\bar{x}})$, $x_k^*\rightarrow x^*$ for some $x^*\in {\mathbb {X}}$, and $x_k^*\in \partial \varphi (x_k)$ for each $k\in {\mathbb {N}}$, we also have $x^*\in \partial \varphi ({\bar{x}})$, see [71, Proposition 8.7]. This property is referred to as robustness of the limiting subdifferential.

In the case where $\varphi$ is locally Lipschitzian around ${\bar{x}}$, and for some direction $u\in {\mathbb {X}}$,

$$\begin{aligned} \partial \varphi ({\bar{x}};u):= \left\{ \eta \in {\mathbb {X}}\,\left| \, \begin{aligned}&\exists \{u_k\}_{k\in {\mathbb {N}}}\subset {\mathbb {X}},\, \exists \{t_k\}_{k\in {\mathbb {N}}}\subset {\mathbb {R}}_+,\, \exists \{\eta _k\}_{k\in {\mathbb {N}}}\subset {\mathbb {X}}:\\&\qquad u_k\rightarrow u,\,t_k\downarrow 0,\,\eta _k\rightarrow \eta ,\, \eta _k\in {\widehat{\partial }}\varphi ({\bar{x}}+t_ku_k)\,\forall k\in {\mathbb {N}}\end{aligned}\right. \right\} \end{aligned}$$

is referred to as the *limiting subdifferential* of $\varphi$ at ${\bar{x}}$ *in direction* *u*. We note that $\partial \varphi ({\bar{x}};0)=\partial \varphi ({\bar{x}})$ and $\partial \varphi ({\bar{x}};u)\subset \partial \varphi ({\bar{x}})$ for all $u\in {\mathbb {X}}$. Furthermore, let us mention that, in the definition of the directional limiting subdifferential, we can equivalently replace the requirement $\eta _k\in {\widehat{\partial }}\varphi ({\bar{x}}+t_ku_k)$ by $\eta _k\in \partial \varphi ({\bar{x}}+t_ku_k)$ for each $k\in {\mathbb {N}}$. This can be easily checked by means of a classical diagonal sequence argument. Hence, the directional limiting subdifferential also enjoys a certain kind of robustness.

#### Graphical derivatives

Below, we introduce three different graphical derivatives of a set-valued mapping. While the standard graphical derivative is well known from the literature, the concepts of graphical pseudo-derivative and graphical subderivative are, to the best of our knowledge, new.

##### Definition 2.2

Let $\varPhi :{\mathbb {X}}\rightrightarrows {\mathbb {Y}}$ be a set-valued mapping possessing a closed graph locally around $({\bar{x}},{\bar{y}})\in {\text {gph}}\varPhi$.

- The *graphical derivative* of $\varPhi$ at $({\bar{x}},{\bar{y}})$ is the mapping $D\varPhi ({\bar{x}},{\bar{y}}):{\mathbb {X}}\rightrightarrows {\mathbb {Y}}$ given by
  $$\begin{aligned} {\text {gph}}D\varPhi ({\bar{x}},{\bar{y}})={\mathcal {T}}_{{\text {gph}}\varPhi }({\bar{x}},{\bar{y}}). \end{aligned}$$
  In the case where $\varPhi$ is single-valued at ${\bar{x}}$, we use $D\varPhi ({\bar{x}}):{\mathbb {X}}\rightrightarrows {\mathbb {Y}}$ for brevity.
- Given $\gamma \ge 1$, the *graphical pseudo-derivative of order* $\gamma$ of $\varPhi$ at $({\bar{x}},{\bar{y}})$ is the mapping $D_{\gamma }\varPhi ({\bar{x}},{\bar{y}}):{\mathbb {X}} \rightrightarrows {\mathbb {Y}}$ which assigns to $u\in {\mathbb {X}}$ the set of all $v\in {\mathbb {Y}}$ such that there are sequences $\{u_k\}_{k\in {\mathbb {N}}}\subset {\mathbb {X}}$, $\{v_k\}_{k\in {\mathbb {N}}}\subset {\mathbb {Y}}$, and $\{t_k\}_{k\in {\mathbb {N}}}\subset {\mathbb {R}}_+$ which satisfy $u_k\rightarrow u$, $v_k\rightarrow v$, $t_k\downarrow 0$, and $({\bar{x}}+t_ku_k,{\bar{y}} + (t_k \left\| u_k\right\| )^{\gamma } v_k)\in {\text {gph}}\varPhi$ for all $k\in {\mathbb {N}}$.
- The *graphical subderivative* of $\varPhi$ at $({\bar{x}},{\bar{y}})$ is the mapping $D_sub \varPhi ({\bar{x}},{\bar{y}}):{\mathbb {S}}_{{\mathbb {X}}} \rightrightarrows {\mathbb {S}}_{{\mathbb {Y}}}$ which assigns to $u\in {\mathbb {S}}_{{\mathbb {X}}}$ the set of all $v\in {\mathbb {S}}_{{\mathbb {Y}}}$ such that there are sequences $\{u_k\}_{k\in {\mathbb {N}}}\subset {\mathbb {X}}$, $\{v_k\}_{k\in {\mathbb {N}}}\subset {\mathbb {Y}}$, and $\{t_k\}_{k\in {\mathbb {N}}},\{\tau _k\}_{k\in {\mathbb {N}}}\subset {\mathbb {R}}_+$ which satisfy $u_k\rightarrow u$, $v_k\rightarrow v$, $t_k\downarrow 0$, $\tau _k\downarrow 0$, $\tau _k/t_k\rightarrow \infty$, and $({\bar{x}}+t_ku_k,{\bar{y}}+\tau _kv_k)\in {\text {gph}}\varPhi$ for all $k\in {\mathbb {N}}$.

Let us note that for every set-valued mapping $\varPhi :{\mathbb {X}}\rightrightarrows {\mathbb {Y}}$, whose graph is closed locally around $({\bar{x}},{\bar{y}})\in {\text {gph}}\varPhi$, we have $D_1\varPhi ({\bar{x}},{\bar{y}})(u)=D\varPhi ({\bar{x}},{\bar{y}})(u)$ for all $u\in {\mathbb {S}}_{{\mathbb {X}}}$. Furthermore, for each $\gamma > 1$, one obtains the trivial estimates

$$\begin{aligned} {\text {dom}}D_\gamma \varPhi ({\bar{x}},{\bar{y}}) \subset \ker D\varPhi ({\bar{x}},{\bar{y}}) \end{aligned}$$

and

2.3 $$\begin{aligned} \forall u\in {\mathbb {S}}_{{\mathbb {X}}}:\quad D_sub \varPhi ({\bar{x}},{\bar{y}})(u) \subset D\varPhi ({\bar{x}},{\bar{y}})(0) \end{aligned}$$

right from the definition of these objects.

In the course of the paper, we are mainly interested in the graphical (sub)derivative associated with so-called normal cone mappings. In the next lemma, we present some corresponding upper estimates.

##### Lemma 2.4

Let $D\subset {\mathbb {Y}}$ be a nonempty, closed, convex set such that the (single-valued) projection operator onto *D*, denoted by $\varPi _D:{\mathbb {Y}}\rightarrow {\mathbb {Y}}$, is directionally differentiable. Fix ${\bar{y}}\in D$ and ${\bar{y}}^*\in {\mathcal {N}}_D({\bar{y}})$. Then, for arbitrary $u\in {\mathbb {Y}}$, we find

$$\begin{aligned} D{\mathcal {N}}_D({\bar{y}},{\bar{y}}^*)(u)&\subset \{v\in {\mathbb {Y}}\,|\,\varPi _D'({\bar{y}}+{\bar{y}}^*;u+v)=u\}, \end{aligned}$$

and for $u\in {\mathbb {S}}_{{\mathbb {Y}}}$, we find

$$\begin{aligned} D_sub {\mathcal {N}}_D({\bar{y}},{\bar{y}}^*)(u)&\subset \{v\in {\mathbb {S}}_{{\mathbb {Y}}}\,|\, \varPi _D'({\bar{y}}+{\bar{y}}^*;v)=0, \langle u, v\rangle \ge 0\}. \end{aligned}$$

Above, $\varPi _D'(y,v)$ denotes the directional derivative of $\varPi _D$ at $y\in {\mathbb {Y}}$ in direction $v\in {\mathbb {Y}}$.

##### Proof

By convexity of *D*, we have the well-known equivalence

$$\begin{aligned} \forall y,y^*\in {\mathbb {Y}}:\quad y^*\in {\mathcal {N}}_D(y) \quad \Longleftrightarrow \quad \varPi _D(y+y^*)=y. \end{aligned}$$

In the remainder of the proof, we set ${{\tilde{y}}}:={\bar{y}}+{\bar{y}}^*$ for brevity. Next, let us fix $u,v\in {\mathbb {Y}}$ as well as $\{u_k\}_{k\in {\mathbb {N}}},\{v_k\}_{k\in {\mathbb {N}}}\subset {\mathbb {Y}}$ and $\{\tau _k\}_{k\in {\mathbb {N}}},\{\varepsilon _k\}_{k\in {\mathbb {N}}}\subset {\mathbb {R}}_+$ such that $u_k\rightarrow u$, $v_k\rightarrow v$, $\tau _k\downarrow 0$, and ${\bar{y}}^*+\tau _kv_k\in {\mathcal {N}}_D({\bar{y}}+\tau _k\varepsilon _k u_k)$, i.e., $\varPi _D({{\tilde{y}}}+\tau _k\varepsilon _ku_k+\tau _kv_k)={\bar{y}}+\tau _k\varepsilon _ku_k$, for each $k\in {\mathbb {N}}$. Using $\varPi _D({{\tilde{y}}})={\bar{y}}$, we find

2.4 $$\begin{aligned} \forall k\in {\mathbb {N}}:\quad \varepsilon _k u_k = \frac{\varPi _D({{\tilde{y}}}+\tau _k\varepsilon _ku_k+\tau _kv_k)-\varPi _D({{\tilde{y}}})}{\tau _k}. \end{aligned}$$

In the case where $v\in D{\mathcal {N}}_D({\bar{y}},{\bar{y}}^*)(u)$ holds, we can choose $\varepsilon _k=1$ for each $k\in {\mathbb {N}}$, and taking the limit $k\rightarrow \infty$ in (2.4) while exploiting directional differentiability and Lipschitzness of $\varPi _D$ yields $\varPi '_D({{\tilde{y}}};u+v)=u$. This shows the first estimate.

Now, assume that $v\in D_sub {\mathcal {N}}_D({\bar{y}},{\bar{y}}^*)(u)$ is valid. Then $\varepsilon _k\downarrow 0$ and $u, v \in {\mathbb {S}}_{{\mathbb {Y}}}$ can be postulated, and taking the limit $k\rightarrow \infty$ in (2.4) shows $\varPi '_D({{\tilde{y}}};v)=0$. By nature of the projection, we have

$$\begin{aligned} \langle {{\tilde{y}}}+\tau _k\varepsilon _ku_k+\tau _kv_k - \varPi _D({{\tilde{y}}}+\tau _k\varepsilon _ku_k+\tau _kv_k) , \varPi _D({{\tilde{y}}}) - \varPi _D({{\tilde{y}}}+\tau _k\varepsilon _ku_k+\tau _kv_k) \rangle \le 0 \end{aligned}$$

for each $k\in {\mathbb {N}}$. Exploiting (2.4), this is equivalent to

$$\begin{aligned} \langle {{\tilde{y}}}+\tau _kv_k-\varPi _D({{\tilde{y}}}) , \varPi _D({{\tilde{y}}}) - \varPi _D({{\tilde{y}}}+\tau _k\varepsilon _ku_k+\tau _kv_k) \rangle \le 0 \end{aligned}$$

for each $k\in {\mathbb {N}}$. Some rearrangements and the characterization of the projection lead to

$$\begin{aligned}&\tau _k\, \langle v_k, \varPi _D({{\tilde{y}}}) - \varPi _D({{\tilde{y}}}+\tau _k\varepsilon _ku_k+\tau _kv_k) \rangle \\&\quad \le \langle {{\tilde{y}}}-\varPi _D({{\tilde{y}}}) , \varPi _D({{\tilde{y}}}+\tau _k\varepsilon _ku_k+\tau _kv_k) - \varPi _D({{\tilde{y}}}) \rangle \le 0. \end{aligned}$$

Division by $\tau _k^2\varepsilon _k$ and (2.4), thus, give us $\langle v_k, u_k \rangle \ge 0$ for each $k\in {\mathbb {N}}$, and taking the limit, we obtain $\langle u, v\rangle \ge 0$ which shows the second estimate. $\square$

Let us note that it has been shown in [75, Theorem 3.1, Corollary 3.1] that the estimate on the graphical derivative of the normal cone mapping ${\mathcal {N}}_D$ holds as an equality in the situation where *D* is the convex cone of positive semidefinite symmetric matrices, and that the presented proof extends to arbitrary convex cones as long as the associated projection operator is directionally differentiable. This result can also be found in slightly more general form in [67, Theorem 3.3]. In order to make the estimates from Lemma 2.4 explicit, one needs to be in position to characterize the directional derivative of the projection onto the convex set *D*. This is easily possible if *D* is polyhedral, see [44] and Remark 3.1, but even in nonpolyhedral situations, e.g., where *D* is the second-order cone or the cone of positive semidefinite symmetric matrices, closed formulas for this directional derivative are available in the literature, see [69, Lemma 2] and [73, Theorem 4.7], respectively.

The following technical result will become handy later on.

##### Lemma 2.5

Let $D\subset {\mathbb {Y}}$ be nonempty and closed, and fix ${\bar{y}}\in D$. Then the following assertions hold.

- For each $u\in {\mathbb {Y}}$, we have $D{\mathcal {N}}_D({\bar{y}},0)(u)\subset {\mathcal {N}}_D({\bar{y}};u)$.
- For each $u\in {\mathbb {S}}_{{\mathbb {Y}}}$, we have $D_sub {\mathcal {N}}_D({\bar{y}},0)(u)\subset {\mathcal {N}}_D({\bar{y}};u)$.

##### Proof

We only prove validity of the first assertion. The second one can be shown in analogous fashion.

Fix $u\in {\mathbb {Y}}$ and $v\in D{\mathcal {N}}_D({\bar{y}},0)(u)$. Then we find sequences $\{u_k\}_{k\in {\mathbb {N}}},\{v_k\}_{k\in {\mathbb {N}}}\subset {\mathbb {Y}}$ and $\{t_k\}_{k\in {\mathbb {N}}}\subset {\mathbb {R}}_+$ with $u_k\rightarrow u$, $v_k\rightarrow v$, $t_k\downarrow 0$, and $t_kv_k\in {\mathcal {N}}_D({\bar{y}}+t_ku_k)$ for each $k\in {\mathbb {N}}$. Since, for each $k\in {\mathbb {N}}$, ${\mathcal {N}}_D({\bar{y}}+t_ku_k)$ is a cone, we find $v_k\in {\mathcal {N}}_D({\bar{y}}+t_ku_k)$, and $v\in {\mathcal {N}}_D({\bar{y}};u)$ follows by robustness of the directional limiting normal cone, see Lemma 2.1. $\square$

In the next two results, we investigate the special situation ${\mathbb {Y}}:={\mathbb {R}}^m$ in detail. First, in the case where we consider the normal cone mapping associated with polyhedral sets, there is no difference between graphical derivative and graphical subderivative as the subsequent lemma shows.

##### Lemma 2.6

Let $D\subset {\mathbb {R}}^m$ be a polyhedral set. Then ${\text {gph}}{\mathcal {N}}_D$ is polyhedral as well, and for arbitrary $({\bar{y}},{\bar{y}}^*)\in {\text {gph}}{\mathcal {N}}_D$ and $u, v\in {\mathbb {R}}^m {\setminus } \{0\}$, we have

$$\begin{aligned} v \in D{\mathcal {N}}_D({\bar{y}},{\bar{y}}^*)(u) \quad \Longleftrightarrow \quad v/\left\| v\right\| \in D_sub {\mathcal {N}}_D({\bar{y}},{\bar{y}}^*)(u/\left\| u\right\| ). \end{aligned}$$

##### Proof

It follows from [1, Theorem 2] that there exist finitely many convex polyhedral sets $D_1,\ldots ,D_\ell \subset {\mathbb {R}}^m$ and closed, convex, polyhedral cones $K_1,\ldots ,K_\ell \subset {\mathbb {R}}^m$ such that ${\text {gph}}{\mathcal {N}}_D=\bigcup _{i=1}^\ell D_i\times K_i$. Particularly, ${\text {gph}}{\mathcal {N}}_D$ is polyhedral.

Next, consider some nonzero $u, v\in {\mathbb {R}}^m$ with $v/\left\| v\right\| \in D_sub {\mathcal {N}}_D({\bar{y}},{\bar{y}}^*)(u/\left\| u\right\| )$. Then we find $\{{{\tilde{u}}}_k\}_{k\in {\mathbb {N}}},\{{{\tilde{v}}}_k\}_{k\in {\mathbb {N}}}\subset {\mathbb {R}}^m$ and $\{{{\tilde{t}}}_k\}_{k\in {\mathbb {N}}},\{\tau _k\}_{k\in {\mathbb {N}}}\subset {\mathbb {R}}_+$ such that $u_k:={{\tilde{u}}}_k \left\| u\right\| \rightarrow u$, $v_k:={{\tilde{v}}}_k \left\| v\right\| \rightarrow v$, $t_k:= {{\tilde{t}}}_k/\left\| u\right\| \downarrow 0$, $\tau _k\downarrow 0$, $\tau _k/t_k\rightarrow \infty$, as well as $({\bar{y}}+t_k u_k,{\bar{y}}^*+ (\tau _k/\left\| v\right\| ) v_k)\in {\text {gph}}{\mathcal {N}}_D$ for all $k\in {\mathbb {N}}$. Thus, we can pick $j\in \{1,\ldots ,\ell \}$ and a subsequence (without relabeling) such that $({\bar{y}}+t_k u_k,{\bar{y}}^*+ (\tau _k/\left\| v\right\| ) v_k) \in D_j\times K_j$ and $\tau _k/\left\| v\right\| >t_k$ for all $k\in {\mathbb {N}}$. By convexity of $K_j$, we also have $({\bar{y}}+t_k u_k,{\bar{y}}^*+ t_k v_k) \in D_j\times K_j$ which shows $v\in D{\mathcal {N}}_D({\bar{y}},{\bar{y}}^*)(u)$. The converse implication can be proven in analogous fashion by multiplying the null sequence in the domain space with another null sequence. $\square$

The next lemma shows how the graphical derivative of normal cone mappings associated with Cartesian products of polyhedral sets can be computed.

##### Lemma 2.7

Fix some $\ell \in {\mathbb {N}}$. For each $i\in \{1,\ldots ,\ell \}$, let $D_i\subset {\mathbb {R}}^{m_i}$ for some $m_i\in {\mathbb {N}}$ be polyhedral. Set $D:=\prod _{i=1}^\ell D_i$, $m:=\sum _{i=1}^\ell m_i$, and $L:=\{1,\ldots ,\ell \}$. Then we have

$$\begin{aligned} {\text {gph}}{\mathcal {N}}_D = \{((y_1,\ldots ,y_\ell ),(y_1^*,\ldots ,y_\ell ^*))\in {\mathbb {R}}^m\times {\mathbb {R}}^m\,|\, \forall i\in L:\,(y_i,y_i^*)\in {\text {gph}}{\mathcal {N}}_{D_i}\}, \end{aligned}$$

and for arbitrary ${\bar{y}}:=({\bar{y}}_1,\ldots ,{\bar{y}}_\ell ),{\bar{y}}^*:=({\bar{y}}_1^*,\ldots ,{\bar{y}}_\ell ^*)\in {\mathbb {R}}^m$ satisfying $({\bar{y}},{\bar{y}}^*)\in {\text {gph}}{\mathcal {N}}_D$ as well as $u:=(u_1,\ldots ,u_\ell )\in {\mathbb {R}}^m$, we find

$$\begin{aligned} D{\mathcal {N}}_D({\bar{y}},{\bar{y}}^*)(u) = \{v=(v_1,\ldots ,v_\ell )\in {\mathbb {R}}^m\,|\, \forall i\in L:\,v_i\in D{\mathcal {N}}_{D_i}({\bar{y}}_i,{\bar{y}}_i^*)(u_i)\}. \end{aligned}$$

##### Proof

The representation of ${\text {gph}}{\mathcal {N}}_D$ is a simple consequence of the product rule for the computation of limiting normals, see e.g. [66, Proposition 1.4], and does not rely on the polyhedrality of the underlying sets. Thus, ${\text {gph}}{\mathcal {N}}_D$ is, up to a permutation of components, the same as $\prod _{i=1}^\ell {\text {gph}}{\mathcal {N}}_{D_i}$. Since, for each $i\in L$, ${\text {gph}}{\mathcal {N}}_{D_i}$ is polyhedral by Lemma 2.6, the same has to hold for ${\text {gph}}{\mathcal {N}}_D$. The final formula of the lemma is a simple consequence of Lemma 2.3 and [71, Exercise 6.7]. $\square$

#### Coderivatives, pseudo-coderivatives, and super-coderivatives

In the subsequently stated definition, we first recall the notion of regular and limiting coderivative of a set-valued mapping before introducing its so-called directional pseudo-coderivative. The latter will be of essential importance in the course of the paper. It corresponds to a minor modification of the notion of directional pseudo-coderivative introduced by Gfrerer in [37, Definition 2], which we recall as well.

##### Definition 2.3

Let $\varPhi :{\mathbb {X}}\rightrightarrows {\mathbb {Y}}$ be a set-valued mapping possessing a closed graph locally around $({\bar{x}},{\bar{y}})\in {\text {gph}}\varPhi$. Furthermore, let $(u,v)\in {\mathbb {X}}\times {\mathbb {Y}}$ be a pair of directions.

- The *regular and limiting coderivative* of $\varPhi$ at $({\bar{x}},{\bar{y}})$ are the set-valued mappings ${{\widehat{D}}}^*\varPhi ({\bar{x}},{\bar{y}}):{\mathbb {Y}}\rightrightarrows {\mathbb {X}}$ and $D^*\varPhi ({\bar{x}},{\bar{y}}):{\mathbb {Y}}\rightrightarrows {\mathbb {X}}$ given, respectively, by
  $$\begin{aligned} \forall y^*\in {\mathbb {Y}}:\quad {{\widehat{D}}}^*\varPhi ({\bar{x}},{\bar{y}})(y^*)&:= \{x^*\in {\mathbb {X}}\,|\, (x^*,-y^*)\in \widehat{{\mathcal {N}}}_{{\text {gph}}\varPhi }({\bar{x}},{\bar{y}})\}, \\ D^*\varPhi ({\bar{x}},{\bar{y}})(y^*)&:= \{x^*\in {\mathbb {X}}\,|\, (x^*,-y^*)\in {\mathcal {N}}_{{\text {gph}}\varPhi }({\bar{x}},{\bar{y}})\}. \end{aligned}$$
  The set-valued mapping $D^*\varPhi (({\bar{x}},{\bar{y}});(u,v)):{\mathbb {Y}}\rightrightarrows {\mathbb {X}}$ given by
  $$\begin{aligned}&\forall y^*\in {\mathbb {Y}}:\quad D^*\varPhi (({\bar{x}},{\bar{y}});(u,v))(y^*)\\&\qquad \qquad \qquad \qquad := \{x^*\in {\mathbb {X}}\,|\, (x^*,-y^*)\in {\mathcal {N}}_{{\text {gph}}\varPhi }(({\bar{x}},{\bar{y}});(u,v))\} \end{aligned}$$
  is the *limiting coderivative* of $\varPhi$ at $({\bar{x}},{\bar{y}})$ *in direction* (*u*, *v*). If $\varPhi$ is single-valued at ${\bar{x}}$, we use ${{\widehat{D}}}^*\varPhi ({\bar{x}}),D^*\varPhi ({\bar{x}}),D^*\varPhi ({\bar{x}};(u,v)) :{\mathbb {Y}}\rightrightarrows {\mathbb {X}}$ for brevity.
- Given $\gamma \ge 1$ and $u\in {\mathbb {S}}_{{\mathbb {X}}}$, the *pseudo-coderivative of order* $\gamma$ of $\varPhi$ at $({\bar{x}},{\bar{y}})$ *in direction (u, v)* is the mapping $D^*_{\gamma } \varPhi (({\bar{x}},{\bar{y}}); (u,v)):{\mathbb {Y}}\rightrightarrows {\mathbb {X}}$ which assigns to $y^*\in {\mathbb {Y}}$ the set of all $x^*\in {\mathbb {X}}$ such that there are sequences $\{u_k\}_{k\in {\mathbb {N}}},\{x_k^*\}_{k\in {\mathbb {N}}}\subset {\mathbb {X}}$, $\{v_k\}_{k\in {\mathbb {N}}},\{y_k^*\}_{k\in {\mathbb {N}}}\subset {\mathbb {Y}}$, and $\{t_k\}_{k\in {\mathbb {N}}}\subset {\mathbb {R}}_+$ which satisfy $u_k\rightarrow u$, $v_k\rightarrow v$, $t_k\downarrow 0$, $x_k^*\rightarrow x^*$, $y_k^*\rightarrow y^*$, and
  2.5 $$\begin{aligned} \forall k\in {\mathbb {N}}:\quad \left( x^*_k,-\frac{y^*_k}{(t_k\left\| u_k\right\| )^{\gamma -1}}\right) \in \widehat{{\mathcal {N}}}_{{\text {gph}}\varPhi }({\bar{x}}+ t_k u_k,{\bar{y}}+ (t_k\left\| u_k\right\| )^{\gamma } v_k). \nonumber \\ \end{aligned}$$
  In the case $\gamma :=1$, this definition recovers the one of $D^*\varPhi (({\bar{x}},{\bar{y}});(u,v))$.
- Given $\gamma \ge 1$ and $u\in {\mathbb {S}}_{{\mathbb {X}}}$, *Gfrerer’s pseudo-coderivative of order* $\gamma$ of $\varPhi$ at $({\bar{x}},{\bar{y}})$ *in direction (u, v)* is the mapping ${{\widetilde{D}}}^*_{\gamma } \varPhi (({\bar{x}},{\bar{y}}); (u,v)):{\mathbb {Y}}\rightrightarrows {\mathbb {X}}$ which assigns to $y^*\in {\mathbb {Y}}$ the set of all $x^*\in {\mathbb {X}}$ such that there are sequences $\{u_k\}_{k\in {\mathbb {N}}},\{x_k^*\}_{k\in {\mathbb {N}}}\subset {\mathbb {X}}$, $\{v_k\}_{k\in {\mathbb {N}}},\{y_k^*\}_{k\in {\mathbb {N}}}\subset {\mathbb {Y}}$, and $\{t_k\}_{k\in {\mathbb {N}}}\subset {\mathbb {R}}_+$ which satisfy $u_k\rightarrow u$, $v_k\rightarrow v$, $t_k\downarrow 0$, $x_k^*\rightarrow x^*$, $y_k^*\rightarrow y^*$, and
  2.6 $$\begin{aligned} \forall k\in {\mathbb {N}}:\quad \left( x^*_k,-\frac{y^*_k}{(t_k\left\| u_k\right\| )^{\gamma -1}}\right) \in \widehat{{\mathcal {N}}}_{{\text {gph}}\varPhi }({\bar{x}}+ t_k u_k,{\bar{y}}+ t_k v_k). \end{aligned}$$
  Again, for $\gamma :=1$, we recover the definition of $D^*\varPhi (({\bar{x}},{\bar{y}});(u,v))$.

Let $\varPhi :{\mathbb {X}}\rightrightarrows {\mathbb {Y}}$ be a set-valued mapping whose graph is closed locally around $({\bar{x}},{\bar{y}})\in {\text {gph}}\varPhi$ and fix a pair of directions $(u,v)\in {\mathbb {S}}_{{\mathbb {X}}}\times {\mathbb {Y}}$, $(x^*,y^*) \in {\mathbb {X}}\times {\mathbb {Y}}$, and $\gamma >1$. Then we obtain the trivial relations

2.7 $$\begin{aligned} x^* \in D^*_\gamma \varPhi (({\bar{x}},{\bar{y}});(u,v))(y^*) \ \Longrightarrow \ \left\{ \begin{aligned}&0 \in D\varPhi ({\bar{x}},{\bar{y}})(u), \ 0 \in D^*\varPhi ({\bar{x}},{\bar{y}})(y^*), \\&0 \in D^*\varPhi (({\bar{x}},{\bar{y}});(u,0))(y^*),\\&v \in D_\gamma \varPhi ({\bar{x}},{\bar{y}})(u),\\&x^* \in {{\widetilde{D}}}^*_\gamma \varPhi (({\bar{x}},{\bar{y}});(u,0))(y^*). \end{aligned} \right. \end{aligned}$$

Note also that the mappings $D^*\varPhi (({\bar{x}},{\bar{y}});(u,v))$ and ${{\widetilde{D}}}^*_\gamma \varPhi (({\bar{x}},{\bar{y}});(u,v))$ have a nonempty graph if and only if $v \in D\varPhi ({\bar{x}},{\bar{y}})(u)$ while the mapping $D^*_\gamma \varPhi (({\bar{x}},{\bar{y}});(u,v))$ has a nonempty graph if and only if $v \in D_\gamma \varPhi ({\bar{x}},{\bar{y}})(u)$.

Since the (directional) limiting coderivative is defined via the (directional) limiting normal cone, it possesses a robust behavior as well. In the subsequent lemma, we show a somewhat robust behavior of the directional pseudo-coderivatives under consideration, which will be important later on. Basically, we prove that one can replace the regular by the limiting normal cone in (2.5) and (2.6) without changing the resulting pseudo-coderivative. The technical proof, which is based on a standard diagonal sequence argument, is presented in Appendix A for the purpose of completeness.

##### Lemma 2.8

Definition 2.3 (b) and Definition 2.3 (c) can equivalently be formulated in terms of limiting normals.

To illustrate the pseudo-coderivatives from Definition 2.3, we revisit Example 2.1.

##### Example 2.2

For $\gamma > 1$, we consider the mapping $\varPhi :{\mathbb {R}}\rightrightarrows {\mathbb {R}}$, given by $\varPhi (x):= \{ \vert x \vert ^\gamma \}$, $x\in {\mathbb {R}}$, already discussed in Example 2.1. Set $({\bar{x}}, {\bar{y}}):= (0,0)$ as well as $u:=\pm 1$ and choose $v\in {\mathbb {R}}$ arbitrarily. First, $v \in D \varPhi ({\bar{x}},{\bar{y}})(u)$ by definition requires sequences $\{t_k\}_{k\in {\mathbb {N}}}\subset {\mathbb {R}}_+$ and $\{u_k\}_{k\in {\mathbb {N}}},\{v_k\}_{k\in {\mathbb {N}}}\subset {\mathbb {R}}$ satisfying $t_k \downarrow 0$, $u_k \rightarrow u$, $v_k \rightarrow v$, and $t_k v_k = (t_k \vert u_k \vert )^\gamma$ for all $k\in {\mathbb {N}}$, showing $v=0$. Thus, we fix $v:=0$ to find $D^*\varPhi (({\bar{x}},{\bar{y}});(u,0))(y^*) = \{0\}$ for all $y^*\in {\mathbb {R}}$ as the defining sequences $\{x_k^*\}_{k\in {\mathbb {N}}},\{y_k^*\}_{k\in {\mathbb {N}}}\subset {\mathbb {R}}$ satisfy $x_k^* \rightarrow x^*$, $y_k^* \rightarrow y^*$, and $x_k^* = \gamma (t_k \vert u_k \vert )^{\gamma -1} {\text {sgn}}(u_k) y_k^*$ for all $k\in {\mathbb {N}}$. Furthermore, ${{\widetilde{D}}}^*_{\gamma } \varPhi (({\bar{x}},{\bar{y}}); (u,0))(y^*) = \{\gamma {\text {sgn}}(u) y^*\}$ holds for each $y^*\in {\mathbb {R}}$ as the defining sequences $\{x_k^*\}_{k\in {\mathbb {N}}},\{y_k^*\}_{k\in {\mathbb {N}}}\subset {\mathbb {R}}$ satisfy $x_k^* \rightarrow x^*$, $y_k^* \rightarrow y^*$, and $x_k^* = \gamma {\text {sgn}}(u_k) y_k^*$ for all $k\in {\mathbb {N}}$. Using similar arguments as above, one can check that $v \in D_\gamma \varPhi ({\bar{x}},{\bar{y}})(u)$ yields $v=1$, and for $v:=1$, we get $D^*_\gamma \varPhi (({\bar{x}},{\bar{y}});(u,1))(y^*)=\{\gamma {\text {sgn}}(u)y^*\}$ for all $y^*\in {\mathbb {R}}$.

Below, we introduce yet another concept of coderivative which will become important in Sect. 5.3.

##### Definition 2.4

Let $\varPhi :{\mathbb {X}}\rightrightarrows {\mathbb {Y}}$ be a set-valued mapping with a closed graph and fix $({\bar{x}},{\bar{y}})\in {\text {gph}}\varPhi$ and $(u,v)\in {\mathbb {S}}_{{\mathbb {X}}}\times {\mathbb {S}}_{{\mathbb {Y}}}$. The *super-coderivative* of $\varPhi$ at $({\bar{x}},{\bar{y}})$ in direction (*u*, *v*) is the mapping $D^*_sup \varPhi (({\bar{x}},{\bar{y}}); (u,v)):{\mathbb {Y}} \rightrightarrows {\mathbb {X}}$, which assigns to $y^* \in {\mathbb {Y}}$ the set of all $x^*\in {\mathbb {X}}$ such that there are sequences $\{u_k\}_{k\in {\mathbb {N}}},\{x_k^*\}_{k\in {\mathbb {N}}}\subset {\mathbb {X}}$, $\{v_k\}_{k\in {\mathbb {N}}},\{y_k^*\}_{k\in {\mathbb {N}}}\subset {\mathbb {Y}}$, and $\{t_k\}_{k\in {\mathbb {N}}},\{\tau _k\}_{k\in {\mathbb {N}}}\subset {\mathbb {R}}_+$ which satisfy $u_k\rightarrow u$, $v_k\rightarrow v$, $x_k^*\rightarrow x^*$, $y_k^*\rightarrow y^*$, $t_k\downarrow 0$, $\tau _k\downarrow 0$, and $\tau _k/t_k\rightarrow 0$ such that

2.8 $$\begin{aligned} x_k^* \in {\widehat{D}}^*\varPhi ({\bar{x}}+ t_k u_k,{\bar{y}}+ \tau _k v_k) (((t_k \left\| u_k\right\| )/(\tau _k \left\| v_k\right\| )) y_k^*) \end{aligned}$$

holds for all $k\in {\mathbb {N}}$.

We start with some remarks regarding Definition 2.4. First, observe that we only exploit the super-coderivative w.r.t. unit directions $(u,v)\in {\mathbb {S}}_{{\mathbb {X}}}\times {\mathbb {S}}_{{\mathbb {Y}}}$ which also means that $\{u_k\}_{k\in {\mathbb {N}}}\subset {\mathbb {X}}$ and $\{v_k\}_{k\in {\mathbb {N}}}\subset {\mathbb {Y}}$ can be chosen such that $u_k\ne 0$ and $v_k\ne 0$ hold for all $k\in {\mathbb {N}}$. Particularly, condition (2.8) is reasonable.

Second, we would like to note that $x^*\in D^*_sup \varPhi (({\bar{x}},{\bar{y}});(u,v))(y^*)$ implies the existence of $\{u_k\}_{k\in {\mathbb {N}}}\subset {\mathbb {X}}$, $\{v_k\}_{k\in {\mathbb {N}}}\subset {\mathbb {Y}}$, and $\{t_k\}_{k\in {\mathbb {N}}},\{\tau _k\}_{k\in {\mathbb {N}}}\subset {\mathbb {R}}_+$ which satisfy $u_k\rightarrow u$, $v_k\rightarrow v$, $t_k\downarrow 0$, $\tau _k\downarrow 0$, and $\tau _k/t_k\rightarrow 0$ as well as $({\bar{x}}+t_ku_k,{\bar{y}}+\tau _kv_k)\in {\text {gph}}\varPhi$ for all $k\in {\mathbb {N}}$. Thus, in the light of Definition 2.2 (c) of the graphical subderivative, one might be tempted to say that the pair (*u*, *v*) belongs to the graph of the graphical *super-derivative* of $\varPhi$ at $({\bar{x}},{\bar{y}})$. This justifies the terminology in Definition 2.4.

Let us briefly discuss the relation between pseudo-coderivatives and the novel super-coderivative from Definition 2.4. Consider $\gamma > 1$ and $x^* \in D^*_\gamma \varPhi (({\bar{x}},{\bar{y}}); (u,v))(y^*)$ for $(u,v)\in {\mathbb {S}}_{{\mathbb {X}}}\times {\mathbb {S}}_{{\mathbb {Y}}}$ and $y^*\in {\mathbb {Y}}^*$. Setting $\tau _k:= (t_k \left\| u_k\right\| )^{\gamma }$ for each $k\in {\mathbb {N}}$, where $\{t_k\}_{k\in {\mathbb {N}}}\subset {\mathbb {R}}_+$ and $\{u_k\}_{k\in {\mathbb {N}}}\subset {\mathbb {X}}$ are the sequences from the definition of the pseudo-coderivative, we get $x^* \in D^*_sup \varPhi (({\bar{x}},{\bar{y}}); (u,v))(y^*)$ since $t_k^{\gamma -1}\left\| u_k\right\| ^\gamma \rightarrow 0$.

In the subsequent lemma, we comment on the converse inclusion which, to some extent, holds in the presence of a qualification condition in terms of the pseudo-coderivative.

##### Lemma 2.9

Let $({\bar{x}},{\bar{y}})\in {\text {gph}}\varPhi$, $(u,v)\in {\mathbb {S}}_{{\mathbb {X}}}\times {\mathbb {S}}_{{\mathbb {Y}}}$, $y^* \in {\mathbb {Y}}$, and $\gamma > 1$ be fixed. Furthermore, assume that $\ker D^*_\gamma \varPhi (({\bar{x}},{\bar{y}});(u,0))\subset \{0\}$ holds. Then there exists $\alpha > 0$ such that

$$\begin{aligned} D^*_sup \varPhi (({\bar{x}},{\bar{y}});(u,v))(y^*)&\subset {\widetilde{D}}^*_\gamma \varPhi (({\bar{x}},{\bar{y}});(u,0))(0) \cup D^*_\gamma \varPhi (({\bar{x}},{\bar{y}});(u,\alpha v))(y^*/\alpha ) \\&\qquad \cup {\text {Im}}D^*_\gamma \varPhi (({\bar{x}},{\bar{y}});(u,0)) \\&\subset {\text {Im}}{{\widetilde{D}}}^*_\gamma \varPhi (({\bar{x}},{\bar{y}});(u,0)). \end{aligned}$$

##### Proof

Let $x^*\in D^*_sup \varPhi (({\bar{x}},{\bar{y}});(u,v))(y^*)$ be arbitrarily chosen. Then we find sequences $\{u_k\}_{k\in {\mathbb {N}}},\{x_k^*\}_{k\in {\mathbb {N}}}\subset {\mathbb {X}}$, $\{v_k\}_{k\in {\mathbb {N}}},\{y_k^*\}_{k\in {\mathbb {N}}}\subset {\mathbb {Y}}$, and $\{t_k\}_{k\in {\mathbb {N}}},\{\tau _k\}_{k\in {\mathbb {N}}}\subset {\mathbb {R}}_+$ which satisfy $u_k\rightarrow u$, $v_k\rightarrow v$, $x_k^*\rightarrow x^*$, $y_k^*\rightarrow y^*$, $t_k\downarrow 0$, $\tau _k\downarrow 0$, and $\tau _k/t_k\rightarrow 0$ as well as (2.8) for all $k\in {\mathbb {N}}$. This also gives us

2.9 $$\begin{aligned} x_k^* \in {{\widehat{D}}}^*\varPhi \left( {\bar{x}}+t_ku_k, {\bar{y}}+(t_k\left\| u_k\right\| )^\gamma \frac{\tau _kv_k}{(t_k\left\| u_k\right\| )^\gamma } \right) \left( (t_k\left\| u_k\right\| )^{1-\gamma } \frac{(t_k\left\| u_k\right\| )^\gamma }{\tau _k\left\| v_k\right\| }y_k^* \right) \nonumber \\ \end{aligned}$$

for all $k\in {\mathbb {N}}$. Set ${{\tilde{y}}}_k^*:=(t_k\left\| u_k\right\| )^\gamma /(\tau _k\left\| v_k\right\| )y_k^*$ for each $k\in {\mathbb {N}}$. In the case where $\{{{\tilde{y}}}_k^*\}_{k\in {\mathbb {N}}}$ is not bounded, we have $(\tau _k\left\| v_k\right\| )/(t_k\left\| u_k\right\| )^\gamma \rightarrow 0$ along a subsequence (without relabeling), and taking the limit in

$$\begin{aligned} x_k^*/\Vert {{\tilde{y}}}_k^*\Vert \in {{\widehat{D}}}^*\varPhi \left( {\bar{x}}+t_ku_k, {\bar{y}}+(t_k\left\| u_k\right\| )^\gamma \frac{\tau _kv_k}{(t_k\left\| u_k\right\| )^\gamma } \right) \left( (t_k\left\| u_k\right\| )^{1-\gamma } {{\tilde{y}}}_k^*/\Vert {{\tilde{y}}}_k^*\Vert \right) \end{aligned}$$

yields that $\ker D^*_\gamma \varPhi (({\bar{x}},{\bar{y}});(u,0))$ contains a nonzero element, which is a contradiction. Hence, $\{{{\tilde{y}}}_k^*\}_{k\in {\mathbb {N}}}$ is bounded.

For each $k\in {\mathbb {N}}$, we set $\alpha _k:=\tau _k\left\| v_k\right\| /(t_k\left\| u_k\right\| )^\gamma$. First, suppose that $\{\alpha _k\}_{k\in {\mathbb {N}}}$ is not bounded. Then, along a subsequence (without relabeling), we may assume $\alpha _k\rightarrow \infty$. By boundedness of $\{y_k^*\}_{k\in {\mathbb {N}}}$, ${{\tilde{y}}}_k^*\rightarrow 0$ follows. Rewriting (2.9) yields

$$\begin{aligned} x_k^*\in {{\widehat{D}}}^*\varPhi \left( {\bar{x}}+t_ku_k,{\bar{y}}+t_k\frac{\tau _kv_k}{t_k}\right) \left( (t_k\left\| u_k\right\| )^{1-\gamma }{{\tilde{y}}}_k^*\right) \end{aligned}$$

for each $k\in {\mathbb {N}}$, and taking the limit $k\rightarrow \infty$ while respecting $\tau _k/t_k\rightarrow 0$, thus, gives $x^*\in {{\widetilde{D}}}^*_\gamma \varPhi (({\bar{x}},{\bar{y}});(u,0))(0)$. In the case where $\{\alpha _k\}_{k\in {\mathbb {N}}}$ converges to some $\alpha >0$ (along a subsequence without relabeling), we can simply take the limit $k\rightarrow \infty$ in (2.9) in order to find $x^*\in D^*_\gamma \varPhi (({\bar{x}},{\bar{y}});(u,\alpha v))(y^*/\alpha )$. Finally, let us consider the case $\alpha _k\rightarrow 0$ (along a subsequence without relabeling). Then, by boundedness of $\{{{\tilde{y}}}_k^*\}_{k\in {\mathbb {N}}}$, taking the limit $k\rightarrow \infty$ in (2.9) gives $x^*\in {\text {Im}}D^*_\gamma \varPhi (({\bar{x}},{\bar{y}});(u,0))$. Thus, we have shown the first inclusion.

The second inclusion follows by the upper estimate (2.7) for the pseudo-coderivative. $\square$

#### Sufficient conditions for pseudo-(sub)regularity

Graphical derivative and (directional) limiting coderivative are powerful tools for studying regularity properties of set-valued mappings, such as (strong) metric regularity and subregularity, as well as their inverse counterparts of Lipschitzness, such as Aubin property and (isolated) calmness. Indeed, given a closed-graph set-valued mapping $\varPhi :{\mathbb {X}}\rightrightarrows {\mathbb {Y}}$, metric regularity and strong metric subregularity at some point $({\bar{x}},{\bar{y}}) \in {\text {gph}}\varPhi$ are characterized, respectively, by

2.10a $$\begin{aligned} \ker D^*\varPhi ({\bar{x}},{\bar{y}})&= \{0\}, \end{aligned}$$

2.10b $$\begin{aligned} \ker D\varPhi ({\bar{x}},{\bar{y}})&= \{0\}, \end{aligned}$$

see e.g. [60, 66, 71] for the definition of these Lipschitzian properties as well as the above results. Let us mention that (2.10a) is referred to as *Mordukhovich criterion* in the literature, while (2.10b) is called *Levy–Rockafellar criterion*.

For fixed $u\in {\mathbb {S}}_{{\mathbb {X}}}$, we will refer to

2.11 $$\begin{aligned} \ker D^*\varPhi (({\bar{x}},{\bar{y}});(u,0)) \subset \{0\}, \end{aligned}$$

which implies that $\varPhi$ is metrically subregular at $({\bar{x}},{\bar{y}})$ in direction *u*, see e.g. [36, Theorem 5], as FOSCMS(*u*). Note that it is formulated as an inclusion as the left-hand side in (2.11) is empty whenever $u\notin \ker D\varPhi ({\bar{x}},{\bar{y}})$. Indeed, in this case, $\varPhi$ is trivially metrically subregular at $({\bar{x}},{\bar{y}})$ in direction *u*. Furthermore, whenever (2.11) holds for all $u\in \ker D\varPhi ({\bar{x}},{\bar{y}})\cap {\mathbb {S}}_{{\mathbb {X}}}$, which we will refer to as FOSCMS, then $\varPhi$ is already metrically subregular at $({\bar{x}},{\bar{y}})$, see [38, Lemma 2.7]. Above, FOSCMS abbreviates *First-Order Sufficient Condition for Metric Subregularity*, and this terminology has been coined in [36]. Clearly, each of the conditions from (2.10) is sufficient for FOSCMS. The relations (2.7) suggest that the pseudo-coderivative can be useful particularly in situations where the above regularity properties, which are related to (first-order) coderivatives, fail.

Note that the aforementioned notions of regularity and Lipschitzness express certain linear rate of change of the mapping. Similarly, there is an underlying linearity in the definition of graphical derivative and coderivatives. Take the graphical derivative for instance. Since the same sequence $\{t_k\}_{k\in {\mathbb {N}}}$ appears in the domain as well as in the range space, if $v \in D\varPhi ({\bar{x}},{\bar{y}})(u)$ implies that $u\in {\mathbb {X}}$ and $v\in {\mathbb {Y}}$ are both nonzero, it suggests a proportional (linear) rate of change. Thus, in order to characterize pseudo-(sub)regularity of order $\gamma >1$ of $\varPhi$, it is not very surprising that we need to exploit derivative-like objects based on sub- or superlinear structure. Exemplary, this has been successfully visualized in [37, Corollary 2] by means of Gfrerer’s directional pseudo-coderivative of order $\gamma >1$ from Definition 2.3 (c). Here, we show that the fundamental result from [37, Theorem 1(2)] yields also an analogous sufficient condition for metric pseudo-subregularity via the pseudo-coderivative from Definition 2.3 (b).

##### Lemma 2.10

Let $\varPhi :{\mathbb {X}}\rightrightarrows {\mathbb {Y}}$ be a set-valued mapping having a closed graph locally around $({\bar{x}},{\bar{y}})\in {\text {gph}}\varPhi$, fix a direction $u\in {\mathbb {S}}_{{\mathbb {X}}}$, and some $\gamma \ge 1$. Assume that

2.12 $$\begin{aligned} \ker D^*_{\gamma } \varPhi (({\bar{x}},{\bar{y}}); (u,0)) \subset \{0\} \end{aligned}$$

holds. Then $\varPhi$ is metrically pseudo-subregular of order $\gamma$ at $({\bar{x}},{\bar{y}})$ in direction *u*.

##### Proof

Suppose that $\varPhi$ is not metrically pseudo-subregular of order $\gamma$ at $({\bar{x}},{\bar{y}})$ in direction *u*. Due to [37, Theorem 1(2)], we find sequences $\{t_k\}_{k\in {\mathbb {N}}}\subset {\mathbb {R}}_+$, $\{u_k\}_{k\in {\mathbb {N}}},\{x_k^*\}_{k\in {\mathbb {N}}}\subset {\mathbb {X}}$, and $\{v_k\}_{k\in {\mathbb {N}}},\{y_k^*\}_{k\in {\mathbb {N}}}\subset {\mathbb {Y}}$ satisfying (among other things) $t_k\downarrow 0$, $u_k\rightarrow u$, $t_k^{1-\gamma }v_k\rightarrow 0$, as well as $x_k^*\rightarrow 0$, such that $\vert \vert {y_k^*}\vert \vert =1$ and

$$\begin{aligned} (x_k^*,-y_k^*/(t_k\left\| u_k\right\| )^{\gamma -1}) \in \widehat{{\mathcal {N}}}_{{\text {gph}}\varPhi }(({\bar{x}},{\bar{y}})+t_k(u_k,v_k)) \end{aligned}$$

for each $k\in {\mathbb {N}}$. Let us set ${{\tilde{v}}}_k:=t_k^{1-\gamma }\left\| u_k\right\| ^{-\gamma } v_k$ for each $k\in {\mathbb {N}}$. Then we have

$$\begin{aligned} (x_k^*,-y_k^*/(t_k\left\| u_k\right\| )^{\gamma -1}) \in \widehat{{\mathcal {N}}}_{{\text {gph}}\varPhi }({\bar{x}}+t_ku_k,{\bar{y}}+(t_k\left\| u_k\right\| )^\gamma {{\tilde{v}}}_k) \end{aligned}$$

for each $k\in {\mathbb {N}}$ and ${{\tilde{v}}}_k\rightarrow 0$ from $t_k^{1-\gamma }v_k\rightarrow 0$. Observing that $\{y_k^*\}_{k\in {\mathbb {N}}}$ possesses a nonvanishing accumulation point $y^*\in {\mathbb {Y}}$, taking the limit along a suitable subsequence yields $0\in D^*_\gamma \varPhi (({\bar{x}},{\bar{y}});(u,0))(y^*)$ which contradicts the assumptions of the lemma. $\square$

Let us remark that due to (2.7), condition

2.13 $$\begin{aligned} \ker {\widetilde{D}}^*_\gamma \varPhi (({\bar{x}},{\bar{y}});(u,0)) \subset \{0\} \end{aligned}$$

is stronger than (2.12) and, thus, also sufficient for metric pseudo-subregularity of $\varPhi$ of order $\gamma \ge 1$ at $({\bar{x}},{\bar{y}})$ in direction *u*. By means of [37, Corollary 2], (2.13) is actually equivalent to $\varPhi$ being metrically pseudo-regular at $({\bar{x}},{\bar{y}})$ in direction (*u*, 0). Note that in the case $\gamma :=1$, both conditions (2.12) and (2.13) recover FOSCMS(*u*). In Example 2.2, (2.12) and (2.13) hold simultaneously. The following example illustrates that (2.12) can be strictly milder than (2.13).

##### Example 2.3

For $\gamma > 1$, we consider the mapping $\varPhi :{\mathbb {R}}\rightrightarrows {\mathbb {R}}$ given by

$$\begin{aligned} {\text {gph}}\varPhi := \{(x,y)\,|\,|x|^\gamma \le y \le 2|x|^\gamma \} \cap \left( \bigcup \nolimits _{k\in {\mathbb {N}}}{\mathbb {R}}\times \{1/2^k\}\right) . \end{aligned}$$

Essentially, ${\text {gph}}\varPhi$ is a closed staircase enclosed by the graphs of the functions $x\mapsto |x|^\gamma$ and $x\mapsto 2|x|^\gamma$. Set $({\bar{x}}, {\bar{y}}):= (0,0)$ and $u:=1$. First, it is easy to see that (2.12) is satisfied, because one can show $\ker D^*_\gamma \varPhi (({\bar{x}},{\bar{y}});(u,0)) = \emptyset$. Indeed, the sequences $\{t_k\}_{k\in {\mathbb {N}}}\subset {\mathbb {R}}_+$ and $\{u_k\}_{k\in {\mathbb {N}}},\{v_k\}_{k\in {\mathbb {N}}}\subset {\mathbb {R}}$ from the definition of the pseudo-coderivative satisfy, among others, $t_k \downarrow 0$, $u_k \rightarrow u$, $v_k \rightarrow v$, and $(t_k |u_k|)^\gamma \le (t_k |u_k|)^\gamma v_k \le 2 (t_k |u_k|)^\gamma$ for each $k\in {\mathbb {N}}$. Thus, $D^*_\gamma \varPhi (({\bar{x}},{\bar{y}});(u,v))$ can have a nonempty graph only for $v\in [1,2]$. Next, let us argue that (2.13) fails due to $1 \in \ker {\widetilde{D}}^*_\gamma \varPhi (({\bar{x}},{\bar{y}});(u,0))$. We consider the sequences $\{t_k\}_{k\in {\mathbb {N}}}\subset {\mathbb {R}}_+$ and $\{u_k\}_{k\in {\mathbb {N}}},\{v_k\}_{k\in {\mathbb {N}}}\subset {\mathbb {R}}$ given by

$$\begin{aligned} \forall k\in {\mathbb {N}}:\quad t_k:= \left( \frac{3}{2^{k+2}}\right) ^{1/\gamma }, \qquad u_k:= 1, \qquad v_k:= \frac{1}{2^{k}} \left( \frac{3}{2^{k+2}}\right) ^{-1/\gamma }. \end{aligned}$$

We obviously have $t_k\downarrow 0$, $u_k\rightarrow 1$, as well as $v_k\rightarrow 0$, and one can easily check that $(t_ku_k,t_kv_k)\in {\text {gph}}\varPhi$ holds for all $k\in {\mathbb {N}}$. By construction, there exist vertical normals to ${\text {gph}}\varPhi$ at $(t_k u_k, t_k v_k)$ for each $k\in {\mathbb {N}}$, so we can choose $x_k^*:= 0$ and $y_k^*:= 1$ satisfying (2.6). Taking the limit $k\rightarrow \infty$ shows $1 \in \ker {\widetilde{D}}^*_\gamma \varPhi (({\bar{x}},{\bar{y}});(u,0))$.

##### Remark 2.1

Let $\varPhi :{\mathbb {X}}\rightrightarrows {\mathbb {Y}}$ be a set-valued mapping having locally closed graph around $({\bar{x}},{\bar{y}})\in {\text {gph}}\varPhi$, and fix some $\gamma \ge 1$. Note that if we replace the set $\varPhi ^{-1}({\bar{y}})$ by just the singleton $\{{\bar{x}}\}$ in Definition 2.1 of metric pseudo-subregularity, the estimate (2.2) simplifies to

$$\begin{aligned} \left\| x-{\bar{x}}\right\| ^{\gamma } \le \kappa \,{\text {dist}}({\bar{y}},\varPhi (x)). \end{aligned}$$

Asking this to hold for all $x\in {\mathbb {B}}_\varepsilon ({\bar{x}})$ and some $\varepsilon >0$ seems like a natural way to define *strong metric pseudo-subregularity* of order $\gamma$ of $\varPhi$ at $({\bar{x}},{\bar{y}})$. It is an easy exercise to verify that this condition is satisfied if and only if $\ker D_{\gamma }\varPhi ({\bar{x}},{\bar{y}})=\{0\}$. This characterization is clearly an extension of the Levy–Rockafellar criterion (2.10b), and it provides a justification for the graphical pseudo-derivative.

Finally, by definition of the pseudo-coderivatives, we easily find the inclusions

$$\begin{aligned} \ker D^*_{\gamma +\varepsilon }\varPhi (({\bar{x}},{\bar{y}});(u,0))&\subset \ker D^*_\gamma \varPhi (({\bar{x}},{\bar{y}});(u,0)),\\ \ker {{\widetilde{D}}}^*_{\gamma +\varepsilon }\varPhi (({\bar{x}},{\bar{y}});(u,0))&\subset \ker {{\widetilde{D}}}^*_\gamma \varPhi (({\bar{x}},{\bar{y}});(u,0)) \end{aligned}$$

for each $\gamma \ge 1$ and $\varepsilon >0$. Hence, as $\gamma$ increases, the qualification conditions (2.12) and (2.13) become weaker.

## Pseudo-(sub)regularity of order 2 for constraint mappings

In this section, we address the pseudo-coderivative calculus for so-called constraint mappings $\varPhi :{\mathbb {X}}\rightrightarrows {\mathbb {Y}}$ which are given by $\varPhi (x):=g(x)-D$ for all $x\in {\mathbb {X}}$, where $g:{\mathbb {X}}\rightarrow {\mathbb {Y}}$ is a single-valued continuous function and $D\subset {\mathbb {Y}}$ is a closed set, and apply our findings from Sect. 2.3.4 in order to derive sufficient conditions for directional metric pseudo-(sub)regularity of order 2. Let us emphasize that this representation of $\varPhi$ will be a standing assumption in the overall section. The constraint mapping $\varPhi$ plays an important role for the analysis of so-called geometric constraint systems of type $g(x)\in D$.

### Directional pseudo-coderivatives and sufficient conditions

The first lemma of this subsection addresses upper estimates of the regular, limiting, and directional limiting coderivative of constraint mappings. These results are in principle quite standard, with the exception of the lower estimates in (a) and (c), which can be shown using [20, Theorem 3.1] and [18, Lemma 6.1], respectively. However, since we proceed in a fairly mild setting where *g* is assumed to be merely continuous, we cannot simply rely on change-or-coordinates formulas, see e.g. [71, Exercise 6.7], even for the proof of the standard parts in (a) and (b). Thus, we prove everything using the results from our recent paper [20].

#### Lemma 3.1

Fix $(x,y)\in {\text {gph}}\varPhi$. Then the following statements hold.

- For each $y^*\in {\mathbb {Y}}$, we have
  $$\begin{aligned}{} & {} {\widehat{D}}^*\varPhi (x,y)(y^*) \subset {\left\{ \begin{array}{ll} {\widehat{D}}^*g(x) (y^*) &{} y^*\in \widehat{{\mathcal {N}}}_D(g(x)-y),\\ \emptyset &{} \text {otherwise,} \end{array}\right. } \end{aligned}$$
  and the opposite inclusion holds if *g* is calm at *x*.
- For each $y^*\in {\mathbb {Y}}$, we have
  $$\begin{aligned} D^*\varPhi (x,y)(y^*) \subset {\left\{ \begin{array}{ll} D^*g(x) (y^*) &{} y^*\in {\mathcal {N}}_D(g(x)-y),\\ \emptyset &{} \text {otherwise}, \end{array}\right. } \end{aligned}$$
  and the opposite inclusion holds whenever *g* is continuously differentiable at *x*.
- For each pair of directions $(u,v)\in {\mathbb {X}}\times {\mathbb {Y}}$ and each $y^*\in {\mathbb {Y}}$, we have
  $$\begin{aligned}{} & {} D^*\varPhi ((x,y);(u,v))(y^*)\\{} & {} \qquad \subset {\left\{ \begin{array}{ll} \bigcup \limits _{w\in Dg(x)(u)} D^*g(x;(u,w))(y^*) &{} y^*\in {\mathcal {N}}_D(g(x)-y;w-v),\\ \emptyset &{}\text {otherwise} \end{array}\right. } \end{aligned}$$
  provided *g* is calm at *x*, and the opposite inclusion holds whenever *g* is continuously differentiable at *x*.

#### Proof

- For the proof, we observe that ${\text {gph}}\varPhi ={\text {gph}}g+(\{0\}\times (-D))$ is valid. Now, we exploit the sum rule from [20]. Therefore, let us introduce the surrogate mapping $M:{\mathbb {X}}\times {\mathbb {Y}}\rightrightarrows ({\mathbb {X}}\times {\mathbb {Y}}) \times ({\mathbb {X}}\times {\mathbb {Y}})$ given by
  3.1 $$\begin{aligned} M(x,y):= & {} \left\{ ((x_1,y_1),(x_2,y_2))\in {\text {gph}}g\times (\{0\}\times (-D))\,\left| \, \begin{aligned} x&=x_1+x_2\\ y&=y_1+y_2 \end{aligned}\right. \right\} \nonumber \\= & {} {\left\{ \begin{array}{ll} \{((x,g(x)),(0,y-g(x)))\} &{} g(x)-y\in D,\\ \emptyset &{} \text {otherwise} \end{array}\right. } \end{aligned}$$
  for all $(x,y)\in {\mathbb {X}}\times {\mathbb {Y}}$, and observe that ${\text {gph}}\varPhi ={\text {dom}}M$ holds while *M* is single-valued and continuous on ${\text {gph}}\varPhi$. Now, we find
  $$\begin{aligned} \widehat{{\mathcal {N}}}_{{\text {gph}}\varPhi }(x,y) \subset {\widehat{D}}^*M((x,y),((x,g(x)),(0,y-g(x))))((0,0),(0,0)) \end{aligned}$$
  for all $(x,y)\in {\text {gph}}\varPhi$ from [20, Theorem 3.1], and the converse inclusion holds if *g* is calm at *x* since this ensures that *M* is so-called isolatedly calm at the point of interest, see [20, Corollary 4.4, Section 5.1.1]. Now, computing the regular normal cone to ${\text {gph}}M$ via [20, Lemmas 2.1, 2.2] and applying the definition of the regular coderivative yields the claim.
- The proof of the inclusion $\subset$ is similar as the one of the first statement. Again, we exploit the mapping *M* given in (3.1) and apply [20, Theorem 3.1] while observing that *M* is so-called inner semicompact w.r.t. its domain at each point $(x,y)\in {\text {gph}}\varPhi$ by continuity of *g*. In the presence of continuous differentiability, the converse inclusion $\supset$ follows easily by applying the change-of-coordinates formula provided in [71, Exercise 6.7].
- This assertion can be shown in similar way as the second one, see [20, Lemma 2.1] as well.

$$\square $$

Let us note that the upper estimate in (a) was also shown in [15, Lemma 3.2], but it actually follows directly from [71, Exercise 6.44] upon realizing ${\text {gph}}\varPhi ={\text {gph}}g+(\{0\}\times (-D))$. In the case where *g* is not calm at the reference point, one can still obtain an upper estimate for the directional limiting coderivative from [20, Theorem 3.1] which is slightly more technical since it comprises another union over $w\in Dg(x)(0)\cap {\mathbb {S}}_{{\mathbb {Y}}}$.

Next, we estimate the directional pseudo-coderivatives of order 2 of constraint mappings in terms of initial problem data.

#### Theorem 3.1

Let *g* be twice continuously differentiable. Given $({\bar{x}},0) \in {\text {gph}}\varPhi$ and a direction $u \in {\mathbb {S}}_{{\mathbb {X}}}$, let

$$\begin{aligned} x^* \in {\widetilde{D}}^*_{2} \varPhi (({\bar{x}},0);(u,v))(y^*) \end{aligned}$$

for some $v, y^*\in {\mathbb {Y}}$. Then there exists $z^*\in {\mathbb {Y}}$ such that

3.2a $$\begin{aligned} x^*&= \nabla ^2\langle y^*,g\rangle ({\bar{x}})(u) + \nabla g({\bar{x}})^* z^*, \end{aligned}$$

3.2b $$\begin{aligned} y^*&\in {\mathcal {N}}_{D}(g({\bar{x}});\nabla g({\bar{x}}) u - v) \cap \ker \nabla g({\bar{x}})^*. \end{aligned}$$

Further specifications of $z^*$ satisfying (3.2) are available under additional assumptions.

- Each of the following two conditions
  3.3a $$\begin{aligned}&D{\mathcal {N}}_{D}(g({\bar{x}}),y^*)(0) \cap \ker \nabla g({\bar{x}})^*=\{0\}, \end{aligned}$$
  3.3b $$\begin{aligned} \nabla g({\bar{x}})u \ne v, \quad&D_{sub }{\mathcal {N}}_{D}(g({\bar{x}}),y^*) \left( \frac{\nabla g({\bar{x}})u - v}{\left\| \nabla g({\bar{x}})u - v\right\| } \right) \cap \ker \nabla g({\bar{x}})^* =\emptyset \qquad \end{aligned}$$
  implies that we can find $z^* \in D{\mathcal {N}}_{D}(g({\bar{x}}),y^*)(\nabla g({\bar{x}})u - v)$ satisfying (3.2).
- If $${\mathbb {Y}}:={\mathbb {R}}^m$$ and *D* is locally polyhedral around $$g({\bar{x}})$$, then $${\mathcal {N}}_{D}(g({\bar{x}});\nabla g({\bar{x}})$$$$u - v)={\mathcal {N}}_{{\mathcal {T}}_D(g({\bar{x}}))}(\nabla g({\bar{x}}) u - v)$$, and there are two elements $$z^*_1,z^*_2\in {\mathbb {R}}^m$$ satisfying (3.2) (for $$z^*:=z_i^*$$ with $$i=1,2$$, respectively) with $$z_1^*\in {\mathcal {N}}_{{\mathcal {T}}_D(g({\bar{x}}))}(\nabla g({\bar{x}}) u - v)$$ and $$z_2^*\in {\mathcal {T}}_{{\mathcal {N}}_{{\mathcal {T}}_D(g({\bar{x}}))}(\nabla g({\bar{x}}) u - v)}(y^*)$$.

#### Proof

Since $x^* \in {\widetilde{D}}^*_{2} \varPhi (({\bar{x}},0);(u,v))(y^*)$, we find sequences $\{t_k\}_{k\in {\mathbb {N}}}\subset {\mathbb {R}}_+$, $\{u_k\}_{k\in {\mathbb {N}}},\{x_k^*\}_{k\in {\mathbb {N}}}\subset {\mathbb {X}}$, and $\{v_k\}_{k\in {\mathbb {N}}},\{y_k^*\}_{k\in {\mathbb {N}}}\subset {\mathbb {Y}}$ with $t_k\downarrow 0$, $u_k\rightarrow u$, $v_k\rightarrow v$, $x_k^*\rightarrow x^*$, $y_k^*\rightarrow y^*$, as well as

$$\begin{aligned} (x_k^*,-y_k^*/\tau _k) \in \widehat{{\mathcal {N}}}_{{\text {gph}}\varPhi }({\bar{x}}+ t_ku_k,t_k v_k) \end{aligned}$$

for all $k\in {\mathbb {N}}$ where we used $\tau _k:= t_k \left\| u_k\right\|$ for brevity of notation. Lemma 3.1 yields $x_k^*=\nabla g({\bar{x}}+t_ku_k)^* y_k^*/\tau _k$ and $y_k^*\in \tau _k\widehat{{\mathcal {N}}}_D(g({\bar{x}}+t_ku_k)-t_k v_k)$ for each $k\in {\mathbb {N}}$. Taking the limit in $\tau _kx_k^*=\nabla g({\bar{x}}+t_ku_k)^* y_k^*$, we find $y^*\in \ker \nabla g({\bar{x}})^*$. Combining this with a Taylor expansion and denoting ${{\tilde{w}}}_k:=g({\bar{x}}+t_ku_k)-t_k v_k$ gives us 3.4a3.4b for each $k\in {\mathbb {N}}$. We readily obtain $y^* \in {\mathcal {N}}_{D}(g({\bar{x}});\nabla g({\bar{x}}) u - v)$, i.e., (3.2b), as well as

$$\begin{aligned} x^* - \nabla ^2\langle y^*,g\rangle ({\bar{x}})(u) \in {\text {Im}}\nabla g({\bar{x}})^*, \end{aligned}$$

i.e., (3.2a), due to the closedness of ${\text {Im}}\nabla g({\bar{x}})^*$.

In the general case (a), we will us the identity (3.4a) only with the right-hand side $\nabla g({\bar{x}})^*(y_k^* - y^*)/\tau _k$, but in the polyhedral case (b), it is also reasonable to take a closer look at the expression $\nabla g({\bar{x}})^*y_k^*/\tau _k$.

Let us now prove (a). Using the notation from above, let us first assume that $\{z_k^*\}_{k\in {\mathbb {N}}}$, given by $z_k^*:=(y_k^* - y^*)/\tau _k$ for each $k\in {\mathbb {N}}$, remains bounded. Then we may pass to a subsequence (without relabeling) so that it converges to some $z^*\in {\mathbb {Y}}$. We getand $z^* \in D{\mathcal {N}}_{D}(g({\bar{x}}),y^*)(\nabla g({\bar{x}})u - v)$ follows. Clearly, taking the limit in (3.4a) yields (3.2a) as well.

On the other hand, if $\{z_k^*\}_{k\in {\mathbb {N}}}$ does not remain bounded, we pass to a subsequence (without relabeling) such that $\tau _k/\Vert {y_k^* - y^*}\Vert \rightarrow 0$ and ${\hat{z}}_k^* \rightarrow {\hat{z}}^*$ for some ${{\hat{z}}}^*\in {\mathbb {S}}_{{\mathbb {Y}}}$ where we used ${{\hat{z}}}_k^*:= (y_k^* - y^*)/\Vert {y_k^* - y^*}\Vert$ for each $k\in {\mathbb {N}}$. Multiplying (3.4a) by $\tau _k/\Vert {y_k^* - y^*}\Vert$ and taking the limit yields $\nabla g({\bar{x}})^* {\hat{z}}^* = 0$. Taking into account $({{\tilde{w}}}_k - g({\bar{x}}))/\tau _k \rightarrow \nabla g({\bar{x}})u - v$, we get

3.5 $$\begin{aligned} \frac{\left\| {{\tilde{w}}}_k - g({\bar{x}})\right\| }{\Vert y_k^* - y^*\Vert } = \frac{\left\| {{\tilde{w}}}_k - g({\bar{x}})\right\| }{\tau _k}\frac{\tau _k}{\Vert y_k^* - y^*\Vert } \rightarrow 0. \end{aligned}$$

Let us assume that $\nabla g({\bar{x}})u\ne v$. Then, for sufficiently large $k\in {\mathbb {N}}$, we have ${{\tilde{w}}}_k\ne g({\bar{x}})$, so we can set ${{\hat{q}}}_k:=({{\tilde{w}}}_k-g({\bar{x}}))/\left\| {{\tilde{w}}}_k-g({\bar{x}})\right\|$ for any such $k\in {\mathbb {N}}$ and find ${{\hat{q}}}\in {\mathbb {S}}_{{\mathbb {Y}}}$ such that ${{\hat{q}}}_k\rightarrow {{\hat{q}}}$ (along a subsequence without relabeling). Moreover, we have

$$\begin{aligned} y^* + \Vert y_k^* - y^*\Vert {\hat{z}}_k^* = y_k^* \in \widehat{{\mathcal {N}}}_D\left( g({\bar{x}}) + \left\| {{\tilde{w}}}_k - g({\bar{x}})\right\| {\hat{q}}_k\right) \end{aligned}$$

from (3.4b), so that (3.5) yields ${\hat{z}}^* \in D_{sub }{\mathcal {N}}_{D}(g({\bar{x}}),y^*)({\hat{q}})$. This contradicts (3.3b). In the case where $\nabla g({\bar{x}})u=v$ holds, (3.3b) is not applicable. However, we still have

$$\begin{aligned} y^*+\Vert y_k^*-y^*\Vert {{\hat{z}}}_k^* = y_k^* \in \widehat{{\mathcal {N}}}_D\left( g({\bar{x}})+\Vert y_k^*-y^*\Vert \, \frac{{{\tilde{w}}}_k-g({\bar{x}})}{\Vert y_k^*-y^*\Vert }\right) , \end{aligned}$$

so that taking the limit $k\rightarrow \infty$ while respecting (3.5) yields ${{\hat{z}}}^*\in D{\mathcal {N}}_D(g({\bar{x}}),y^*)(0)$ which contradicts (3.3a).

In the polyhedral case (b), we will show that one can always replace the potentially unbounded sequences from (3.4a) by bounded ones. To start, we prove that $y_k^* \in {\mathcal {N}}_{{\mathcal {T}}_D(g({\bar{x}}))}\left( \nabla g({\bar{x}}) u - v\right)$ for all sufficiently large $k\in {\mathbb {N}}$. Lemma 2.2 (a) yields the existence of a neighborhood $V\subset {\mathbb {R}}^m$ of 0 such that

3.6 $$\begin{aligned} {\mathcal {T}}_D(g({\bar{x}})) \cap V = \big (D - g({\bar{x}}) \big ) \cap V, \end{aligned}$$

as well as the fact that ${\mathcal {T}}_D(g({\bar{x}}))$ is polyhedral. Thus, from (3.4b) we concludefor all sufficiently large $k\in {\mathbb {N}}$.

Next, let us set $K:={\mathcal {N}}_{{\mathcal {T}}_D(g({\bar{x}}))}(\nabla g({\bar{x}})u-v)$ for brevity of notation, and note that *K* is a polyhedral cone. From above we know that $y_k^* \in K$ holds for all sufficiently large $k\in {\mathbb {N}}$. Then we also get $y^*, y_k^*/\tau _k \in K$ and, by Lemma 2.2 (a), $(y_k^* - y^*)/\tau _k \in {\mathcal {T}}_K(y^*)$, where ${\mathcal {T}}_K(y^*)$ is also a polyhedral cone. Thus, referring to (3.4a), we may invoke Hoffman’s lemma, see [31, Lemma 3C.4], to find some bounded sequences $\{z_{1,k}^*\}_{k\in {\mathbb {N}}} \subset K$ and $\{z_{2,k}^*\}_{k\in {\mathbb {N}}} \subset {\mathcal {T}}_K(y^*)$ satisfyingfor $i=1,2$. Thus, accumulation points $z_i^*\in {\mathbb {R}}^m$ of $\{z_{i,k}^*\}_{k\in {\mathbb {N}}}$ for $i=1,2$ satisfy (3.2a) and $z_1^* \in K$ and $z_2^* \in {\mathcal {T}}_K(y^*)$. $\square$

Below, we comment on the findings of Theorem 3.1. To start, we illustrate that the additional information on the multiplier $z^*$ provided in statements (a) and (b) is the same whenever *D* is a convex polyhedral set in ${\mathbb {Y}}:={\mathbb {R}}^m$.

#### Remark 3.1

We use the notation from Theorem 3.1. Suppose that *D* is a *convex* polyhedral set in ${\mathbb {Y}}:={\mathbb {R}}^m$. First, we claim that

$$\begin{aligned} {\mathcal {N}}_{{\mathcal {T}}_D(g({\bar{x}}))}(\nabla g({\bar{x}})u - v)&\subset {\mathcal {N}}_{{\mathcal {T}}_D(g({\bar{x}}))}(\nabla g({\bar{x}})u - v) + {\text {span}}(y^*) \\&= {\mathcal {T}}_{{\mathcal {N}}_{{\mathcal {T}}_D(g({\bar{x}}))}(\nabla g({\bar{x}}) u - v)}(y^*)\\&=D{\mathcal {N}}_{D}(g({\bar{x}}),y^*)(\nabla g({\bar{x}})u - v). \end{aligned}$$

The first two relations are straightforward and so let us prove the last one. Based on the so-called reduction lemma, see [31, Lemma 2E.4], and [31, Proposition 2A.3], for each pair $({\bar{z}}, {\bar{z}}^*) \in {\text {gph}}{\mathcal {N}}_D$, we get

$$\begin{aligned}&\big ({\text {gph}}{\mathcal {N}}_D - ({\bar{z}},{\bar{z}}^*)\big ) \cap {\mathcal {O}} \\&\qquad = \{(w,w^*) \,|\, w \in {\mathcal {K}}_D({\bar{z}},{\bar{z}}^*),\, w^* \in {\mathcal {K}}_D({\bar{z}},{\bar{z}}^*)^{\circ },\, \langle w, w^* \rangle =0\} \cap {\mathcal {O}}, \end{aligned}$$

where ${\mathcal {O}}\subset {\mathbb {R}}^m\times {\mathbb {R}}^m$ is a neighborhood of (0, 0) and ${\mathcal {K}}_D({\bar{z}},{\bar{z}}^*):= {\mathcal {T}}_D({\bar{z}}) \cap [{\bar{z}}^*]^\perp$ represents the *critical cone* to *D* at $({\bar{z}},{\bar{z}}^*)$. By Lemma 2.2 (a), this simply means

$$\begin{aligned} {\mathcal {T}}_{{\text {gph}}{\mathcal {N}}_D}({\bar{z}},{\bar{z}}^*) = \{(w,w^*) \,|\, w \in {\mathcal {K}}_D({\bar{z}},{\bar{z}}^*),\, w^* \in {\mathcal {K}}_D({\bar{z}},{\bar{z}}^*)^{\circ },\, \langle w, w^* \rangle =0\}. \end{aligned}$$

Thus, $z^* \in D{\mathcal {N}}_{D}(g({\bar{x}}),y^*)(\nabla g({\bar{x}})u - v)$ means $\nabla g({\bar{x}})u - v\in {\mathcal {T}}_D(g({\bar{x}})) \cap [y^*]^\perp$, which gives us

$$\begin{aligned} y^* \in {\mathcal {N}}_D(g({\bar{x}}))\cap [\nabla g({\bar{x}})u-v]^\perp = {\mathcal {N}}_{{\mathcal {T}}_D(g({\bar{x}}))}(\nabla g({\bar{x}}) u - v), \end{aligned}$$

and

$$\begin{aligned} z^* \in {\mathcal {N}}_{{\mathcal {K}}_D(g({\bar{x}}),y^*)}(\nabla g({\bar{x}})u - v)&= \big ({\mathcal {T}}_D(g({\bar{x}})) \cap [y^*]^\perp \big )^{\circ } \cap [\nabla g({\bar{x}})u - v]^\perp \\&= \big ({\mathcal {N}}_D(g({\bar{x}}))+{\text {span}}(y^*)\bigr )\cap [\nabla g({\bar{x}})u-v]^\perp \\&= {\mathcal {N}}_D(g({\bar{x}}))\cap [\nabla g({\bar{x}})u-v]^\perp + {\text {span}}(y^*)\\&= {\mathcal {N}}_{{\mathcal {T}}_D(g({\bar{x}}))}(\nabla g({\bar{x}})u - v) + {\text {span}}(y^*)\\&= {\mathcal {T}}_{{\mathcal {N}}_{{\mathcal {T}}_D(g({\bar{x}}))}(\nabla g({\bar{x}}) u - v)}(y^*) \end{aligned}$$

by the basic properties of convex polyhedral cones and Lemma 2.2 (b).

Hence, in the convex polyhedral case, the information on $y^*$ and $z^*$ from statements (a) and (b) (case $z_2^*$) of Theorem 3.1 is the same, while the information from statement (b) (case $z_1^*$) is seemingly sharper. Let us now demonstrate that it is actually also equivalent to the others.

Note that (3.2b) can be equivalently written as $y^* \in {\mathcal {N}}_D(g({\bar{x}}))\cap [v]^\perp \cap \ker \nabla g({\bar{x}})^*$ due to Lemma 2.2 (b) and $[\nabla g({\bar{x}})s-v]^\perp \cap \ker \nabla g({\bar{x}})^*=[v]^\perp \cap \ker \nabla g({\bar{x}})^*$ for all $s\in {\mathbb {X}}$. This also means that, for any such $y^*$, the sets

$$\begin{aligned} A_1(y^*,v)&:= \{s \in {\mathbb {X}} \,|\, \nabla g({\bar{x}})s - v \in {\mathcal {T}}_D(g({\bar{x}}))\}, \\ A_2(y^*,v)&:= \{s \in {\mathbb {X}} \,|\, \nabla g({\bar{x}})s - v \in {\mathcal {K}}_D(g({\bar{x}}),y^*)\} \end{aligned}$$

coincide, and viewing $x^*$, $y^*$, *u*, and *v* as parameters, the linear programs 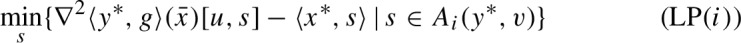 are the same for $i=1,2$. On the other hand, (3.2a) with $z^* \in {\mathcal {N}}_{{\mathcal {T}}_D(g({\bar{x}}))}(\nabla g({\bar{x}})u - v)$ and $z^* \in {\mathcal {N}}_{{\mathcal {K}}_D(g({\bar{x}}),y^*)}(\nabla g({\bar{x}})u - v)$, respectively, precisely characterizes the fact that *u* is a minimizer of LP(1) and LP(2). Hence, this information on $z^*$ is the same.

Some additional comments on Theorem 3.1 are stated subsequently.

#### Remark 3.2

We use the notation from Theorem 3.1.

- Note that, in the case $\nabla g({\bar{x}})u\ne v$, assumption (3.3b), which is stated in terms of the graphical subderivative, is milder than (3.3a) in terms or the standard graphical derivative, and it preserves the connection to the direction $\nabla g({\bar{x}})u - v$. Let us also note that the case $\nabla g({\bar{x}})u=v$ is, anyhow, special since this would annihilate the directional information in (3.2b) completely.
- If ${\mathbb {Y}}:={\mathbb {R}}^m$ and *D* is locally polyhedral around $g({\bar{x}})$, conditions (3.3) reduce to
  $$\begin{aligned} D{\mathcal {N}}_{D}(g({\bar{x}}),y^*)(\nabla g({\bar{x}})u - v) \cap \ker \nabla g({\bar{x}})^* \subset \{0\} \end{aligned}$$
  thanks to Lemma 2.6.

In the polyhedral case, we can derive yet sharper information on $z^*$ if we start with the new pseudo-coderivative instead of the one utilized by Gfrerer. This is also the main reason for introducing the new definition. Throughout the paper, we will rely on the following result. Particularly, it plays an important role in Proposition 5.4 and Corollary 5.3, which we were not able to get using the estimates from Theorem 3.1.

#### Theorem 3.2

Let *g* be twice continuously differentiable. Given $({\bar{x}},0) \in {\text {gph}}\varPhi$, assume that ${\mathbb {Y}}:={\mathbb {R}}^m$ and *D* is locally polyhedral around $g({\bar{x}})$. For a direction $u \in {\mathbb {S}}_{{\mathbb {X}}}$, let

$$\begin{aligned} x^* \in D^*_{2} \varPhi (({\bar{x}},0);(u,v))(y^*) \end{aligned}$$

for some $v, y^*\in {\mathbb {R}}^m$. Then there exists $s\in {\mathbb {X}}$ satisfying $y^* \in {\mathcal {N}}_{{\textbf{T}}(u)}(w_s(u,v))\cap \ker \nabla g({\bar{x}})^*$ where

3.7 $$\begin{aligned} {\textbf{T}}(u):={\mathcal {T}}_{{\mathcal {T}}_D(g({\bar{x}}))}(\nabla g({\bar{x}}) u),\quad w_s(u,v):= \nabla g({\bar{x}}) s + 1/2 \nabla ^2 g({\bar{x}})[u,u] - v,\qquad \end{aligned}$$

together with two elements $z_1^*\in {\mathcal {N}}_{{\textbf{T}}(u)}(w_s(u,v))$ and $z_2^*\in {\mathcal {T}}_{{\mathcal {N}}_{{\textbf{T}}(u)}(w_s(u,v))}(y^*)$ satisfying $x^* = \nabla ^2\langle y^*,g\rangle ({\bar{x}})(u) + \nabla g({\bar{x}})^* z_i^*$ for $i=1,2$. Moreover, $v \in D_2 \varPhi ({\bar{x}},0)(u)$ is equivalent to the existence of $s\in {\mathbb {X}}$ with $w_s(u,v) \in {\textbf{T}}(u)$.

#### Proof

Similar arguments as in the proof of Theorem 3.1 yield (3.4a) together with $y_k^* \in \widehat{{\mathcal {N}}}_D(w_k)$ for each $k\in {\mathbb {N}}$ whereAs in the final part of the proof of Theorem 3.1, all we need to show is $y^*_k \in {\mathcal {N}}_{{\textbf{T}}(u)}(w_s(u,v))$ for all sufficiently large $k\in {\mathbb {N}}$ and some appropriately chosen $s\in {\mathbb {X}}$.

Noting that *D* is polyhedral while ${\textbf{T}}(u)$ is a polyhedral cone, we can apply Lemma 2.2 (a) to find neighborhoods $V,W\subset {\mathbb {R}}^m$ of 0 such that (3.6) and

$$\begin{aligned} {\textbf{T}}(u) \cap W = {\mathcal {T}}_{{\mathcal {T}}_D(g({\bar{x}}))}(\nabla g({\bar{x}}) u) \cap W = \big ({\mathcal {T}}_D(g({\bar{x}})) - \nabla g({\bar{x}}) u \big ) \cap W. \end{aligned}$$

Consequently, we have $w_k - g({\bar{x}}) \in {\mathcal {T}}_D(g({\bar{x}}))$ and, hence, also $\big ( w_k - g({\bar{x}}) \big ) / t_k \in {\mathcal {T}}_D(g({\bar{x}}))$ for sufficiently large $k\in {\mathbb {N}}$. Similarly, we conclude that $z_k \in {\textbf{T}}(u)$. Taking into account that for each cone *K*, $q \in K$, and $\alpha > 0$, one has ${\mathcal {T}}_K(q) = {\mathcal {T}}_K(\alpha q)$, we find

$$\begin{aligned} {\mathcal {T}}_D(w_k)&= {\mathcal {T}}_{g({\bar{x}})+{\mathcal {T}}_D(g({\bar{x}}))}(w_k) = {\mathcal {T}}_{{\mathcal {T}}_D(g({\bar{x}}))}((w_k-g({\bar{x}}))/t_k) \\&= {\mathcal {T}}_{{\mathcal {T}}_D(g({\bar{x}}))}\left( \nabla g({\bar{x}}) u + t_k z_k \right) = {\mathcal {T}}_{{\mathcal {T}}_D(g({\bar{x}}))-\nabla g({\bar{x}})u}\left( z_k\right) = {\mathcal {T}}_{{\textbf{T}}(u)}\left( z_k\right) \end{aligned}$$

for all sufficiently large $k\in {\mathbb {N}}$, and we obtain $y_k^* \in \widehat{{\mathcal {N}}}_D(w_k) = \widehat{{\mathcal {N}}}_{{\textbf{T}}(u)}(z_k)$.

Since ${\textbf{T}}(u)$ is polyhedral, so is ${\text {gph}}{\mathcal {N}}_{{\textbf{T}}(u)}$, see Lemma 2.6, and it can be written as the union of finitely many convex polyhedral sets, say $C_1,\ldots ,C_\ell \subset {\mathbb {R}}^m\times {\mathbb {R}}^m$. Thus, we have

$$\begin{aligned} (z_k,y_k^*) \in {\text {gph}}\widehat{{\mathcal {N}}}_{{\textbf{T}}(u)} \subset {\text {gph}}{\mathcal {N}}_{{\textbf{T}}(u)} = \bigcup _{j=1}^\ell C_j \end{aligned}$$

for sufficiently large $k\in {\mathbb {N}}$. We may pick an index ${\bar{j}}\in \{1,\ldots ,\ell \}$ such that $(z_k,y_k^*) \in C_{{\bar{j}}}$ holds for infinitely many $k\in {\mathbb {N}}$ and suppose that $C_{{\bar{j}}}$ can be represented as $C_{{\bar{j}}} = \{(z,y) \,|\, A z + B y \le c\}$ for some matrices *A*, *B*, as well as *c* of appropriate dimensions. Hence, by passing to a subsequence (without relabeling), we getFor each $k\in {\mathbb {N}}$, a generalized version of Hoffman’s lemma, see [47, Theorem 3], now yields the existence of $s_k\in {\mathbb {X}}$ withfor some constant $\beta > 0$ not depending on *k*. Thus, $\{s_k\}_{k\in {\mathbb {N}}}$ is bounded and satisfiesWe may assume that $\{s_k\}_{k\in {\mathbb {N}}}$ converges to some $s\in {\mathbb {X}}$. Exploiting (3.7), we inferfor all sufficiently large $k\in {\mathbb {N}}$ from polyhedrality of ${\textbf{T}}(u)$ and the definition of the limiting normal cone.

To show the second statement, note that $v \in D_2 \varPhi ({\bar{x}},0)(u)$ is equivalent to $0 \in D^*_{2} \varPhi (({\bar{x}},0);(u,v))(0)$, so that any of these two conditions readily yields the existence of $s\in {\mathbb {X}}$ with $w_s(u,v) \in {\textbf{T}}(u)$. Conversely, suppose that there exists $s\in {\mathbb {X}}$ with $w_s(u,v) \in {\textbf{T}}(u)$. Let $\{t_k\}_{k \in {\mathbb {N}}} \subset {\mathbb {R}}_+$ be an arbitrary sequence with $t_k\downarrow 0$, and define the sequences $\{u_k\}_{k \in {\mathbb {N}}} \subset {\mathbb {X}}$ and $\{v_k\}_{k \in {\mathbb {N}}},\{{{\hat{w}}}_k\}_{k\in {\mathbb {N}}} \subset {\mathbb {Y}}$ by $u_k:= u + t_k s$ and

$$\begin{aligned} v_k:= \big (g({\bar{x}} + t_k u_k) - {{\hat{w}}}_k\big )/(t_k\left\| u_k\right\| )^2, \qquad {{\hat{w}}}_k:= g({\bar{x}}) + t_k \nabla g({\bar{x}}) u + t_k^2 w_s(u,v) \end{aligned}$$

for all $k \in {\mathbb {N}}$. First, a second-order Taylor expansion together with $\left\| u_k\right\| \rightarrow 1$ yields $v_k \rightarrow v$. Next, using similar arguments as before, polyhedrality of ${\mathcal {T}}_D(g({\bar{x}}))$ and, locally around $g({\bar{x}})$, *D*, together with $w_s(u,v) \in {\textbf{T}}(u)$, yields $g({\bar{x}} + t_k u_k) - (t_k\left\| u_k\right\| )^2 v_k = {{\hat{w}}}_k \in D$, i.e., $({\bar{x}}+t_ku_k,(t_k\left\| u_k\right\| )^2v_k)\in {\text {gph}}\varPhi$, for sufficiently large $k\in {\mathbb {N}}$. Taking the limit $k\rightarrow \infty$ gives $v\in D_2\varPhi ({\bar{x}},0)(u)$, and this completes the proof. $\square$

#### Remark 3.3

Let us mention that if ${\mathbb {Y}}:={\mathbb {R}}^m$ and *D* is locally polyhedral around $g({\bar{x}})$, we get the relations

$$\begin{aligned} {\mathcal {N}}_{{\textbf{T}}(u)}(w_s(u,v))&= {\mathcal {N}}_{{\mathcal {T}}_D(g({\bar{x}}))}(\nabla g({\bar{x}})u;w_s(u,v)) \\&\subset {\mathcal {N}}_{{\mathcal {T}}_D(g({\bar{x}}))}(\nabla g({\bar{x}})u)\cap [w_s(u,v)]^\perp \\&\subset {\mathcal {N}}_{{\mathcal {T}}_D(g({\bar{x}}))}(\nabla g({\bar{x}})u) = {\mathcal {N}}_D(g({\bar{x}});\nabla g({\bar{x}})u) = {\mathcal {N}}_{{\textbf{T}}(u)}(0) \end{aligned}$$

from Lemma 2.2 (b). This also yields ${\mathcal {T}}_{{\mathcal {N}}_{{\textbf{T}}(u)}(w_s(u,v))}(y^*) \subset {\mathcal {T}}_{{\mathcal {N}}_{{\mathcal {T}}_D(g({\bar{x}}))}(\nabla g({\bar{x}})u)}(y^*)$.

Again, in the convex polyhedral case, the two options provided by Theorem 3.2 coincide. This can be shown using the same arguments as in Remark 3.1 but with the sets

3.8 $$\begin{aligned} \begin{aligned} {{\widetilde{A}}}_1(y^*,u,v)&:= \left\{ {{\tilde{s}}} \in {\mathbb {X}} \,|\, w_{{{\tilde{s}}}}(u,v) \in {\textbf{T}}(u)\right\} , \\ {{\widetilde{A}}}_2(y^*,u,v)&:= \left\{ {{\tilde{s}}} \in {\mathbb {X}} \,|\, w_{{{\tilde{s}}}}(u,v) \in {\mathcal {K}}_{{\mathcal {T}}_D(g({\bar{x}}))}(\nabla g({\bar{x}})u,y^*)\right\} \end{aligned} \end{aligned}$$

which coincide because the required existence of $s\in {\mathbb {X}}$ with $y^* \in {\mathcal {N}}_{{\textbf{T}}(u)}(w_s(u,v))\cap \ker \nabla g({\bar{x}})^*=({\textbf{T}}(u))^\circ \cap [w_s(u,v)]^\perp \cap \ker \nabla g({\bar{x}})^*$ obviously yields the inclusion $y^*\in [1/2\nabla ^2 g({\bar{x}})[u,u]-v]^\perp$ and, thus, $\langle y^*, w_{{{\tilde{s}}}}(u,v)\rangle = 0$ for all ${{\tilde{s}}} \in {\mathbb {X}}$. This means that our conditions from Theorem 3.2 precisely state that the associated linear programs (LP(*i*)), $i=1,2$, with $A_i(y^*,v)$ replaced by ${{\widetilde{A}}}_i(y^*,u,v)$, have a solution.

From Theorems 3.1 and 3.2 we obtain the following explicit sufficient conditions for metric pseudo-(sub)regularity of constraint mappings.

#### Corollary 3.1

Let *g* be twice continuously differentiable. Consider $({\bar{x}},0) \in {\text {gph}}\varPhi$ and a direction $u \in {\mathbb {S}}_{{\mathbb {X}}}$. The characterization (2.13) of metric pseudo-regularity of order 2 of $\varPhi$ in direction (*u*, 0) at $({\bar{x}},0)$ holds under conditions (a), (b), and (c), while the sufficient condition (2.12) for metric pseudo-subregularity of order 2 of $\varPhi$ in direction *u* at $({\bar{x}},0)$ is valid also under (d).

- One has
  $$\begin{aligned} \left. \begin{aligned}&\nabla g({\bar{x}})^* y^* = 0, \, \nabla ^2\langle y^*,g\rangle ({\bar{x}})(u) + \nabla g({\bar{x}})^* z^* = 0, \\&y^* \in {\mathcal {N}}_{D}(g({\bar{x}});\nabla g({\bar{x}}) u) \end{aligned} \right\} \quad \Longrightarrow \quad y^* = 0. \end{aligned}$$
- One has
  3.9 $$\begin{aligned} \left. \begin{aligned}&\nabla g({\bar{x}})^* y^* = 0, \, \nabla ^2\langle y^*,g\rangle ({\bar{x}})(u) + \nabla g({\bar{x}})^* z^* = 0, \\&y^* \in {\mathcal {N}}_{D}(g({\bar{x}});\nabla g({\bar{x}}) u), \, z^*\in D{\mathcal {N}}_D(g({\bar{x}}),y^*)(\nabla g({\bar{x}})u) \end{aligned} \right\} \quad \Longrightarrow \quad y^* = 0.\nonumber \\ \end{aligned}$$
  Furthermore, we either have
  3.10 $$\begin{aligned} \left. \begin{aligned}&\nabla g({\bar{x}})^* y^* = 0, \, \nabla g({\bar{x}})^* {{\hat{z}}}^* = 0, \\&y^* \in {\mathcal {N}}_{D}(g({\bar{x}});\nabla g({\bar{x}}) u), \, {{\hat{z}}}^* \in D{\mathcal {N}}_D(g({\bar{x}}),y^*)(0) \end{aligned} \right\} \quad \Longrightarrow \quad {{\hat{z}}}^* = 0\nonumber \\ \end{aligned}$$
  or $$\nabla g({\bar{x}})u\ne 0$$ and
  3.11 $$\begin{aligned} \left. \begin{aligned}&\nabla g({\bar{x}})^* y^* = 0, \, \nabla g({\bar{x}})^* {{\hat{z}}}^* = 0, \\&y^* \in {\mathcal {N}}_{D}(g({\bar{x}});\nabla g({\bar{x}}) u) \end{aligned} \right\} \ \Longrightarrow \ {{\hat{z}}}^* \notin D_sub {\mathcal {N}}_D(g({\bar{x}}),y^*) \left( \frac{\nabla g({\bar{x}})u}{\left\| \nabla g({\bar{x}})u\right\| }\right) .\nonumber \\ \end{aligned}$$
- It holds ${\mathbb {Y}}:={\mathbb {R}}^m$, *D* is locally polyhedral around $g({\bar{x}})$, and
  3.12 $$\begin{aligned} \left. \begin{aligned}&\nabla g({\bar{x}})^*y^*=0,\, \nabla ^2\langle y^*,g\rangle ({\bar{x}})(u) + \nabla g({\bar{x}})^* z^*=0,\\&y^*\in {\mathcal {N}}_{{\mathcal {T}}_D(g({\bar{x}}))}(\nabla g({\bar{x}})u),\\&z^*\in {\mathcal {N}}_{{\mathcal {T}}_D(g({\bar{x}}))}(\nabla g({\bar{x}})u)\, \big ( \text {or } \, z^* \in {\mathcal {T}}_{{\mathcal {N}}_{{\mathcal {T}}_ D(g({\bar{x}}))}(\nabla g({\bar{x}})u)}(y^*) \big ) \end{aligned} \right\} \quad \Longrightarrow \quad y^*=0.\nonumber \\ \end{aligned}$$
- It holds ${\mathbb {Y}}:={\mathbb {R}}^m$, *D* is locally polyhedral around $g({\bar{x}})$, and for each $s\in {\mathbb {X}}$ one has
  3.13 $$\begin{aligned} \left. \begin{aligned}&\nabla g({\bar{x}})^* y^* = 0, \, \nabla ^2\langle y^*,g\rangle ({\bar{x}})(u) + \nabla g({\bar{x}})^* z^* = 0, \\&y^*\in {\mathcal {N}}_{{\textbf{T}}(u)}(w_s(u,0)),\\&z^* \in {\mathcal {N}}_{{\textbf{T}}(u)}(w_s(u,0))\,\big ( \text {or } \, z^* \in {\mathcal {T}}_{{\mathcal {N}}_{{\textbf{T}}(u)}(w_s(u,0))}(y^*) \big ) \end{aligned} \right\} \quad \Longrightarrow \quad y^* = 0\qquad \nonumber \\ \end{aligned}$$
  where ${\textbf{T}}(u)$ and $w_s(u,0)$ are given as in (3.7).

Due to Remark 3.3, (3.12) indeed implies validity of (3.13) for arbitrarily chosen $s\in {\mathbb {X}}$.

#### Remark 3.4

Let us note that if ${\mathbb {Y}}:={\mathbb {R}}^m$ and *D* is locally polyhedral around $g({\bar{x}})$, then (3.10) and (3.11) appearing in Corollary 3.1 (b) reduce to

3.14 $$\begin{aligned} \left. \begin{aligned}&\nabla g({\bar{x}})^* y^* = 0, \, \nabla g({\bar{x}})^* {{\hat{z}}}^* = 0, \\&y^* \in {\mathcal {N}}_{D}(g({\bar{x}});\nabla g({\bar{x}}) u), \, {{\hat{z}}}^* \in D{\mathcal {N}}_D(g({\bar{x}}),y^*)(\nabla g({\bar{x}})u) \end{aligned} \right\} \quad \Longrightarrow \quad {{\hat{z}}}^* = 0\nonumber \\ \end{aligned}$$

thanks to Remark 3.2 (b).

### The convex polyhedral case: a comparison with related results

Throughout the subsection, we assume that *D* is a convex polyhedral set in ${\mathbb {Y}}:={\mathbb {R}}^m$, and aim to compare our findings, at least partially, with available results from the literature. To start, we recall the definition of directional 2-regularity taken from [9, Definition 1].

#### Definition 3.1

Set ${\mathbb {Y}}:={\mathbb {R}}^m$, let *D* be convex and polyhedral, and fix $({\bar{x}},0)\in {\text {gph}}\varPhi$ as well as $u\in {\mathbb {X}}$. Then the *2-regularity condition* is said to hold at ${\bar{x}}$ *in direction u* if the following is valid:

3.15 $$\begin{aligned} {\text {Im}}\nabla g({\bar{x}}) + \nabla ^2g({\bar{x}})[u,\nabla g({\bar{x}})^{-1}{\mathcal {T}}_D(g({\bar{x}}))] - {\mathcal {T}}_D(g({\bar{x}})) = {\mathbb {R}}^m. \end{aligned}$$

Let us mention that the original definition of directional 2-regularity from [9, Definition 1] is different from the one stated in Definition 3.1. However, both conditions are equivalent by [9, Proposition 1]. Furthermore, it should be noted that, in the setting of Definition 3.1, the 2-regularity condition in direction $u:=0$ reduces to Robinson’s constraint qualification, see [25, Proposition 2.97]. Observe that, since ${\text {Im}}\nabla g({\bar{x}})$, $\nabla g({\bar{x}})^{-1}{\mathcal {T}}_D(g({\bar{x}}))$, and ${\mathcal {T}}_D(g({\bar{x}}))$ are cones, 2-regularity in a nonzero direction *u* is equivalent to 2-regularity in direction $\alpha u$ for arbitrary $\alpha >0$. Hence, it is reasonable to consider merely directions from ${\mathbb {S}}_{\mathbb {X}}$ in Definition 3.1. In Proposition 3.1 below, we derive a dual characterization of 2-regularity in direction *u*, which states that the conditions 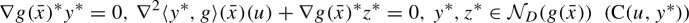 can be satisfied only for $y^* = 0$. Note that (C$(u, y^*)$) can be stated in a $z^*$-free manner by means of

$$\begin{aligned} \nabla g({\bar{x}})^* y^*=0,\, 0 \in \nabla ^2\langle y^*,g\rangle ({\bar{x}})(u)+\nabla g({\bar{x}})^* {\mathcal {N}}_D(g({\bar{x}})),\, y^* \in {\mathcal {N}}_D(g({\bar{x}})) \end{aligned}$$

which is why we did not include $z^*$ in the abbreviation (C$(u, y^*)$).

Second, we will compare our findings with the ones from [37]. Again, we just consider the situation where *D* is a convex polyhedral set. In [37, Theorem 2 (2)], pseudo-subregularity of the feasibility mapping $\varPhi$ of order 2 at $({\bar{x}},0)\in {\text {gph}}\varPhi$ in some direction $u\in {\mathbb {S}}_{{\mathbb {X}}}$ which satisfies $\nabla g({\bar{x}})u\in {\mathcal {T}}_D(g({\bar{x}}))$ (for other directions, the concept is trivial) was shown to be present under the following condition:(C$(u, y^*)$)

3.16 $$\begin{aligned} (\textrm{C}(u, y^*)), y^* \in \mathop {\mathrm {{\text {argmax}}}}\limits \limits _{ {{\hat{y}}}^* \in {\mathcal {N}}_D(g({\bar{x}})) \cap \ker \nabla g({\bar{x}})^* } 1/2\nabla ^2\langle {{\hat{y}}}^* , g\rangle ({\bar{x}})[u,u] \quad \Longrightarrow \quad y^*=0.\nonumber \\ \end{aligned}$$

We will now derive alternative representations of (3.12) and (3.15), which are sufficient for directional pseudo-regularity of $\varPhi$ of order 2, as well as (3.13) and (3.16), being sufficient for directional pseudo-subregularity of $\varPhi$ of order 2, which allow for a comparison of all these conditions.

To start, let us present a technical lemma, collecting some consequences of having $s \in {\mathbb {X}}$ with $w_s(u,0) \in {\textbf{T}}(u)$, see (3.7) for the definition of $w_{s}(u,0)$ and ${\textbf{T}}(u)$.

#### Lemma 3.2

Set ${\mathbb {Y}}:={\mathbb {R}}^m$, let *D* be convex and polyhedral, and fix $({\bar{x}},0)\in {\text {gph}}\varPhi$ as well as $u\in {\mathbb {S}}_{\mathbb {X}}$ such that $\nabla g({\bar{x}})u\in {\mathcal {T}}_D(g({\bar{x}}))$. The existence of $s \in {\mathbb {X}}$ with $w_s(u,0) \in {\textbf{T}}(u)$ is equivalent to the existence of ${{\tilde{s}}} \in {\mathbb {X}}$ with $w_{{{\tilde{s}}}}(u,0) \in {\mathcal {T}}_D(g({\bar{x}}))$, and these conditions imply $\nabla ^2 \langle y^*, g\rangle ({\bar{x}})[u,u]\le 0$ for arbitrary $y^* \in {\mathcal {N}}_D(g({\bar{x}})) \cap \ker \nabla g({\bar{x}})^*$. If, additionally, (C$(u, y^*)$) holds, then we even have $\nabla ^2 \langle y^*, g\rangle ({\bar{x}})[u,u]=0$.

#### Proof

Let us start to prove the first assertion. Note that ${\textbf{T}}(u) = {\mathcal {T}}_D(g({\bar{x}}))+{\text {span}}(\nabla g({\bar{x}})u)$ holds due to polyhedrality of *D* yielding polyhedrality of ${\mathcal {T}}_D(g({\bar{x}}))$. Hence, if $s\in {\mathbb {X}}$ satisfies $w_s(u,0) \in {\textbf{T}}(u)$, then ${{\tilde{s}}}:= s + \alpha u$ for some $\alpha \in {\mathbb {R}}$ satisfies $w_{{{\tilde{s}}}}(u,0) = w_{s}(u,0) + \alpha \nabla g({\bar{x}}) u \in {\mathcal {T}}_D(g({\bar{x}}))$. The converse relation is trivial due to ${\mathcal {T}}_D(g({\bar{x}}))\subset {\textbf{T}}(u)$.

The second assertion is a consequence of the definition of $w_s(u,0)$.

To show the final assertion, note that (C$(u, y^*)$) gives

$$\begin{aligned} \nabla ^2 \langle y^*,g\rangle ({\bar{x}})[u,u] = - \langle z^*, \nabla g({\bar{x}})u\rangle \ge 0 \end{aligned}$$

as $$z^*\in {\mathcal {N}}_D(g({\bar{x}}))$$ and $$\nabla g({\bar{x}})u\in {\mathcal {T}}_D(g({\bar{x}}))$$. $$\square $$

Now, we are in position to state the central result of this subsection.

#### Proposition 3.1

Set ${\mathbb {Y}}:={\mathbb {R}}^m$, let *D* be convex and polyhedral, and fix $({\bar{x}},0)\in {\text {gph}}\varPhi$ as well as $u\in {\mathbb {S}}_{\mathbb {X}}$ such that $\nabla g({\bar{x}})u\in {\mathcal {T}}_D(g({\bar{x}}))$. Then the following statements hold.

- The 2-regularity condition (3.15) is equivalent to the implication
  3.17 $$\begin{aligned} (\textrm{C}(u, y^*)) \quad \Longrightarrow \quad y^*=0. \end{aligned}$$
- Condition (3.12) is equivalent to
  3.18 $$\begin{aligned} (\textrm{C}(u, y^*)),\, \langle z^*, \nabla g({\bar{x}})u\rangle = 0 \quad \Longrightarrow \quad y^*=0. \end{aligned}$$
- Gfrerer’s condition (3.16) and condition (3.13) are both equivalent to
  3.19 $$\begin{aligned} (\textrm{C}(u, y^*)),\, w_s(u,0)\in {\textbf{T}}(u) \quad \Longrightarrow \quad y^*=0. \end{aligned}$$

#### Proof

Let us start to prove (a). If the 2-regularity condition holds at ${\bar{x}}$ in direction *u*, then computing the polar cone on both sides of (3.15) while respecting [23, Exercises 3.4(d) and 3.5] gives

$$\begin{aligned} \{0\} = {\mathcal {N}}_D(g({\bar{x}}))\cap \ker \nabla g({\bar{x}})^* \cap \{y^*\in {\mathbb {R}}^m\,|\,-\nabla ^2\langle y^*,g\rangle ({\bar{x}})(u)\in (\nabla g({\bar{x}})^{-1}{\mathcal {T}}_D(g({\bar{x}})))^\circ \}. \end{aligned}$$

Relying on [23, Exercise 3.5] again while taking convexity and polyhedrality of *D* (and, thus, of ${\mathcal {N}}_D(g({\bar{x}}))$) into account, we find

3.20 $$\begin{aligned} \{0\}= {\mathcal {N}}_D(g({\bar{x}}))\cap \ker \nabla g({\bar{x}})^* \cap \{y^*\in {\mathbb {R}}^m\,|\,-\nabla ^2\langle y^*,g\rangle ({\bar{x}})(u)\in \nabla g({\bar{x}})^*{\mathcal {N}}_D(g({\bar{x}}))\}.\nonumber \\ \end{aligned}$$

Hence, (3.17) holds. Conversely, if (3.17) is valid, then (3.20) holds as well. Computing the polar cone on both sides, we can exploit [23, Exercises 3.4(d) and 3.5] once again in order to obtain

$$\begin{aligned} {\text {cl}}\left( {\text {Im}}\nabla g({\bar{x}})+\nabla ^2g({\bar{x}})[u,\nabla g({\bar{x}})^{-1}{\mathcal {T}}_D(g({\bar{x}}))]-{\mathcal {T}}_D(g({\bar{x}})) \right) = {\mathbb {R}}^m. \end{aligned}$$

Finally, one has to observe that the set within the closure operator is a convex polyhedral cone and, thus, closed in order to find validity of the 2-regularity condition at ${\bar{x}}$ in direction *u*.

Statement (b) follows immediately from Lemma 2.2 (b).

Finally, let us turn to the proof of statement (c). In order to show the equivalence between conditions (3.16) and (3.19), it suffices to prove that (3.16) is equivalent to

3.21 $$\begin{aligned} (\textrm{C}(u, y^*)),\,\nabla ^2\langle y^*, g\rangle ({\bar{x}})[u,u]=0,\, w_{{{\tilde{s}}}}(u,0)\in {\mathcal {T}}_D(g({\bar{x}})) \quad \Longrightarrow \quad y^*=0,\qquad \end{aligned}$$

since the latter is equivalent to (3.19) by Lemma 3.2. The maximization problem appearing in (3.16) is a linear program whose feasible set is a nonempty, convex polyhedral cone. Furthermore, $y^*\in {\mathcal {N}}_D(g({\bar{x}}))\cap \ker \nabla g({\bar{x}})^*$ is a maximizer if and only if

$$\begin{aligned} \begin{aligned} 1/2\nabla ^2g({\bar{x}})[u,u]&\in {\mathcal {N}}_{{\mathcal {N}}_D(g({\bar{x}}))\cap \ker \nabla g({\bar{x}})^*}(y^*) = \left( {\mathcal {N}}_D(g({\bar{x}}))\cap \ker \nabla g({\bar{x}})^*\right) ^\circ \cap [y^*]^\perp \\&= \left( {\mathcal {T}}_D(g({\bar{x}}))+{\text {Im}}\nabla g({\bar{x}})\right) \cap [y^*]^\perp . \end{aligned} \end{aligned}$$

Here, we made use of [23, Exercise 3.4(d)] to compute the polar cone of the appearing intersection, and the latter is a polyhedral cone and, thus, closed. This inclusion, in turn, is equivalent to the existence of ${{\tilde{s}}}\in {\mathbb {X}}$ such that

$$\begin{aligned} \nabla ^2\langle y^*, g\rangle ({\bar{x}})[u,u]=0,\, w_{{{\tilde{s}}}}(u,0)\in {\mathcal {T}}_D(g({\bar{x}})), \end{aligned}$$

showing the claimed equivalence between (3.16) and (3.21) as $y^*\in {\mathcal {N}}_D(g({\bar{x}}))\cap \ker \nabla g({\bar{x}})^*$ is already included in (C$(u, y^*)$).

Clearly, (3.19) implies (3.13) by Lemma 2.2 (b) and Remark 3.3, so we only need to verify the converse implication. Thus, let us prove the premise of (3.13) assuming that (C$(u, y^*)$) holds while there exists some $s \in {\mathbb {X}}$ with $w_s(u,0)\in {\textbf{T}}(u)$. Particularly, from these two we infer $\langle z^*, \nabla g({\bar{x}})u\rangle =0$ with the help of Lemma 3.2, so the premise of (3.18) is valid. Taking into account Remark 3.1, this means that *u* is a solution of the linear program ($\widehat{\text {LP}}(0))$ where we used 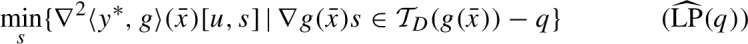 for some parameter $q\in {\mathbb {R}}^m$.

For arbitrary $q\in {\mathbb {R}}^m$, we claim that whether ($\widehat{\text {LP}}(q)$) has a solution depends only on its feasibility since, for feasible problems, the issue of boundedness is independent of *q*. This follows from [17, Lemma 4], stating that, whenever ($\widehat{\text {LP}}(q)$) is feasible, then it possesses a solution if and only if there does not exist $s\in {\mathbb {X}}$ satisfying $\nabla ^2\langle y^*,g\rangle ({\bar{x}})[u,s] < 0$ and

$$\begin{aligned} \nabla g({\bar{x}})s \in ({\mathcal {T}}_D(g({\bar{x}})) - q)^\infty = ({\mathcal {T}}_D(g({\bar{x}})))^\infty = {\mathcal {T}}_D(g({\bar{x}})), \end{aligned}$$

and these conditions are, indeed, independent of *q*. Above, we have used [71, Exercises 3.12 and 6.34(c)]. Since ($\widehat{\text {LP}}(0))$ has a solution, ($\widehat{\text {LP}}(q)$) has a solution for each $q\in {\mathbb {R}}^m$ for which it is feasible. Particularly, Lemma 3.2 thus yields that ($\widehat{\text {LP}}(\bar{q}))$ has a solution ${\bar{s}}\in {\mathbb {X}}$ for ${\bar{q}}:=\nabla ^2\,g ({\bar{x}})[u,u]$.

Finally, we claim that ${\bar{s}}$ is also a solution of the (feasible) linear program

$$\begin{aligned} \min \limits _{{{\tilde{s}}}}\{\nabla ^2\langle y^*, g\rangle ({\bar{x}})[u,{{\tilde{s}}}]\,|\, w_{{{\tilde{s}}}}(u,0)\in {\textbf{T}}(u)\}, \end{aligned}$$

whose feasible set equals the set ${{\widetilde{A}}}_1(y^*,u,0)$ from (3.8). As explained just below (3.8), this will confirm the premise of (3.13) and thus conclude the proof. Suppose that ${\bar{s}}$ is not a solution of this problem, i.e., there exists ${{\hat{s}}}$ with $\nabla ^2\langle y^*,g\rangle ({\bar{x}})[u,{{\hat{s}}} - {\bar{s}}] < 0$ and

$$\begin{aligned} w_{{{\hat{s}}}}(u,0) \in {\textbf{T}}(u) = {\mathcal {T}}_D(g({\bar{x}}))+{\text {span}}(\nabla g({\bar{x}})u). \end{aligned}$$

Then ${{\hat{s}}} + \alpha u$ is a feasible point of ($\widehat{\text {LP}}(\bar{q}))$ for some $\alpha \in {\mathbb {R}}$, while

$$\begin{aligned} \nabla ^2\langle y^*,g\rangle ({\bar{x}})[u,({{\hat{s}}} + \alpha u) - {\bar{s}}] = \nabla ^2\langle y^*,g\rangle ({\bar{x}})[u,{{\hat{s}}} - {\bar{s}}] < 0 \end{aligned}$$

follows from $\nabla ^2\langle y^*,g\rangle ({\bar{x}})[u,u] = 0$ which holds by Lemma 3.2. The latter, however, means that ${\bar{s}}$ is not optimal for ($\widehat{\text {LP}}(\bar{q}))$ - a contradiction. $\square$

Let us mention that the first assertion of Proposition 3.1 generalizes [40, Proposition 2].

As a corollary of Proposition 3.1, we now can easily interrelate the different sufficient conditions for pseudo-(sub)regularity.

#### Corollary 3.2

Set ${\mathbb {Y}}:={\mathbb {R}}^m$, let *D* be convex and polyhedral, and fix $({\bar{x}},0)\in {\text {gph}}\varPhi$ as well as $u\in {\mathbb {S}}_{{\mathbb {X}}}$ such that $\nabla g({\bar{x}})u\in {\mathcal {T}}_D(g({\bar{x}}))$. Then the following implications hold:

$$\begin{aligned} (3.15) \quad \Longrightarrow \quad (3.12) \quad \Longrightarrow \quad (3.16) \quad \Longleftrightarrow \quad (3.13). \end{aligned}$$

Particularly, (3.15) implies that $\varPhi$ is metrically pseudo-regular of order 2 at $({\bar{x}},0)$ in direction (*u*, 0). Moreover, if there exists $s \in {\mathbb {X}}$ with $w_s(u,0)\in {\textbf{T}}(u)$, all four conditions are equivalent.

#### Proof

The first implication and the equivalence are immediately clear by Proposition 3.1. In order to show the second implication, we first make use of Proposition 3.1 in order to see that it suffices to verify that (3.18) implies (3.19). This, however, is clear since (C$(u, y^*)$) and the existence of $s \in {\mathbb {X}}$ such that $w_s(u,0)\in {\textbf{T}}(u)$ imply $\nabla ^2\langle y^*, g\rangle ({\bar{x}})[u,u]=0$, see Lemma 3.2, and $\langle z^*, \nabla g({\bar{x}})u\rangle =0$ follows by (C$(u, y^*)$).

The fact that (3.15) is sufficient for directional pseudo-regularity of $\varPhi$ now follows from Corollary 3.1. The final statement is obvious from Proposition 3.1. $\square$

The following example shows that our sufficient condition (3.12) for directional pseudo-regularity is strictly milder than directional 2-regularity from (3.15).

#### Example 3.1

Let $g:{\mathbb {R}}\rightarrow {\mathbb {R}}^2$ and $D_i \subset {\mathbb {R}}^2$, $i=1,2$, be given by $g(x):= (x,-x^2)$, $x\in {\mathbb {R}}$, and

$$\begin{aligned} D_1:={\mathbb {R}}\times {\mathbb {R}}_+, \qquad D_2:={\mathbb {R}}_-\times {\mathbb {R}}_+. \end{aligned}$$

Observe that $D_i$ is a convex polyhedral set for $i=1,2$. We consider the constraint mappings $\varPhi _i:{\mathbb {R}}\rightrightarrows {\mathbb {R}}^2$ given by $\varPhi _i(x):=g(x)-D_i$, $x\in {\mathbb {R}}$, for $i=1,2$ and fix ${\bar{x}}:=0$ and $u:=-1$. Note that $({\bar{x}},0)\in {\text {gph}}\varPhi _i$ for $i=1,2$.

Let us start with the investigation of the mapping $\varPhi _1$. Due to ${\mathcal {N}}_{D_1}(g({\bar{x}}))=\{0\}\times {\mathbb {R}}_-$ and

$$\begin{aligned} \nabla g({\bar{x}})^*y^*=y_1^*, \qquad \nabla ^2\langle y^*,g\rangle ({\bar{x}})(u) + \nabla g({\bar{x}})^* z^* = 2y_2^* + z_1^*, \end{aligned}$$

one can easily check that (3.17) and (3.18) are both satisfied. Consequently, due to Proposition 3.1, (3.12) and (3.15) hold in parallel.

Let us now consider the mapping $\varPhi _2$. Clearly, (3.18) remains valid since the appearing variable $z^*$ has to be chosen from the set ${\mathcal {N}}_{D_2}(g({\bar{x}})) \cap [\nabla g({\bar{x}})u]^\perp =\{0\} \times {\mathbb {R}}_-$. Hence, due to Proposition 3.1, (3.12) holds (and, thus, pseudo-regularity of order 2 of $\varPhi _2$ in direction *u* at $({\bar{x}},0)$). However, we have ${\mathcal {N}}_{D_2}(g({\bar{x}}))={\mathbb {R}}_+\times {\mathbb {R}}_-$, so that choosing $y^*:=(0,-1)$ and $z^*:=(2,0)$ yields a violation of (3.17) in this situation. Consulting Proposition 3.1 once again, (3.15) is violated as well. Let us also note that, for each $s\in {\mathbb {R}}$, we have

$$\begin{aligned} w_s(u,0) = \nabla g({\bar{x}})s+1/2\nabla ^2g({\bar{x}})[u,u] = (s,-1) \notin {\mathbb {R}}_-\times {\mathbb {R}}_+ = {\mathcal {T}}_{D_2}(g({\bar{x}})), \end{aligned}$$

see Corollary 3.2. Hence, (3.12) is strictly milder than (3.15).

Let us take a closer look at the particular situation where $D:=\{0\}$.

#### Remark 3.5

Set ${\mathbb {Y}}:={\mathbb {R}}^m$, $D:=\{0\}$, and fix $({\bar{x}},0)\in {\text {gph}}\varPhi$ as well as $u\in {\mathbb {S}}_{{\mathbb {X}}}$ such that $u\in \ker \nabla g({\bar{x}})$. Let us consider the sufficient conditions for directional metric pseudo-(sub)regularity discussed in Proposition 3.1. The constraint qualification (3.17) obviously reduces to

3.22 $$\begin{aligned} \nabla g({\bar{x}})^*y^*=0,\, \nabla ^2\langle y^*,g\rangle ({\bar{x}})(u)+\nabla g({\bar{x}})^*z^*=0 \quad \Longrightarrow \quad y^*=0, \end{aligned}$$

and the latter is equivalent to the 2-regularity condition (3.15) at ${\bar{x}}$ in direction *u* by Proposition 3.1. One can easily check that (3.12) also reduces to (3.22). Furthermore, due to Proposition 3.1, (3.13) and (3.16) reduce to

$$\begin{aligned} \nabla g({\bar{x}})^*y^*=0,\, \nabla ^2\langle y^*,g\rangle ({\bar{x}})(u)+\nabla g({\bar{x}})^*z^*=0,\, w_s(u,0)=0 \quad \Longrightarrow \quad y^*=0, \end{aligned}$$

and the latter is strictly milder than (3.22) as we will illustrate in Example 3.2 below.

To close the remark, let us mention that whenever (3.22) has to hold for all $u\in {\mathbb {S}}_{{\mathbb {X}}}$ (this implies metric pseudo-subregularity of order 2 of $\varPhi$ at $({\bar{x}},0)$ for all unit directions), then either $\nabla g({\bar{x}})$ is surjective or the zero operator, see [34, Remark 2.1], i.e., this situation is rather special. We believe, however, that this is mainly because *D* is trivial and partially due to the precise definition of 2-regularity. Let us point the interested reader to [37, Example 2], which suggests that metric pseudo-subregularity of order 2 in all unit directions might be a reasonable assumption.

The following example, which has been motivated by Remark 3.5, indicates that (3.16) is strictly milder than (3.12).

#### Example 3.2

Let $g:{\mathbb {R}}^2\rightarrow {\mathbb {R}}^3$ and $D\subset {\mathbb {R}}^3$ be given by $g(x):=(x_1^2,x_2^2,x_1x_2)$, $x\in {\mathbb {R}}^2$, and $D:=\{0\}$. We consider the point ${\bar{x}}:=0$. As $\nabla g({\bar{x}})$ vanishes while we have ${\mathcal {T}}_D(g({\bar{x}}))=\{0\}$, each direction $u\in {\mathbb {S}}_{{\mathbb {R}}^2}$ satisfies $\nabla g({\bar{x}})u\in {\mathcal {T}}_D(g({\bar{x}}))$, and we pick any such *u*. Due to Remark 3.5, (3.12) and (3.15) reduce to

$$\begin{aligned} y_1^*(2u_1,0)+y_2^*(0,2u_2)+y_3^*(u_2,u_1)=(0,0) \quad \Longrightarrow \quad y^*=0, \end{aligned}$$

and since three vectors in ${\mathbb {R}}^2$ are always linearly dependent, this condition is trivially violated. On the other hand, (3.13) and (3.16) can be stated as

$$\begin{aligned} \left. \begin{aligned}&y_1^*(2u_1,0)+y_2^*(0,2u_2)+y_3^*(u_2,u_1)=(0,0),\\&(u_1^2,u_2^2,u_1u_2)=(0,0,0) \end{aligned} \right\} \quad \Longrightarrow \quad y^*=0, \end{aligned}$$

and this condition holds as the premise regarding *u* cannot be satisfied by any $u\in {\mathbb {S}}_{{\mathbb {R}}^2}$.

We close this subsection with some more general remarks about (directional) 2-regularity and Gfrerer’s sufficient condition for metric pseudo-(sub)regularity from [37, Theorem 2].

In this subsection, for simplicity, we restricted ourselves to the convex polyhedral case, but neither our approach nor the other results are limited to this case. The original definition of directional 2-regularity in [9] is stated for merely convex sets *D* (no polyhedrality is assumed in the latter paper), but involves the radial cone to *D* which is not necessarily closed for curved sets *D*. Interestingly, [37, Example 2], already mentioned in Remark 3.5, provides a mapping which is metrically pseudo-regular of order 2 in every direction (*u*, 0) with $u \ne 0$, particularly metrically pseudo-subregular of order 2 in every unit direction, but the 2-regularity condition is violated for every direction; the chosen set *D* in this example is the Euclidean unit ball in ${\mathbb {R}}^2$ which is not polyhedral.

Let us mention that [37, Theorem 2] is stated in the general polyhedral case (no convexity is assumed), and it yields the existence of several elements $s \in {\mathbb {X}}$ corresponding to the active components of the set *D*. Looking into the proof of Theorem 3.2, it seems like we could get a similar result with only minor adjustments, but since we do not need such a result here, we did not develop this approach for the purpose of brevity.

Let us also note that the conditions from statements (a) and (b) of Corollary 3.1 are not covered by [9] (since *D* does not need to be convex for our findings) or by [37, Theorem 2] (since *D* does not need to be polyhedral).

Finally, let us point out that the concept of 2-regularity is useful for the design and the convergence analysis of Newton-type methods, aiming to solve smooth and nonsmooth equations, see e.g. [33, 49] and the references therein.

## Directional asymptotic stationarity in nonsmooth optimization

This section is devoted to directional asymptotic stationarity conditions and related results. It contains the foundation of our research, Theorem 4.1, which also motivates our considerations in Sect. 5.

For a locally Lipschitz continuous function $\varphi :{\mathbb {X}}\rightarrow {\mathbb {R}}$, a set-valued mapping $\varPhi :{\mathbb {X}}\rightrightarrows {\mathbb {Y}}$ with a closed graph, and ${\bar{y}}\in {\text {Im}}\varPhi$, we investigate the rather abstract optimization problem

P $$\begin{aligned} \min \limits _x\{\varphi (x)\,|\,{\bar{y}}\in \varPhi (x)\}. \end{aligned}$$

Throughout the section, the feasible set of (P) will be denoted by ${\mathcal {F}}\subset {\mathbb {X}}$. Clearly, we have ${\mathcal {F}}\ne \emptyset$ from ${\bar{y}}\in {\text {Im}}\varPhi$. Note that the model (P) covers numerous classes of optimization problems from the literature including standard nonlinear problems, problems with geometric (particularly, disjunctive or conic) constraints, problems with (quasi) variational inequality constraints, and bilevel optimization problems. Furthermore, we would like to mention that choosing ${\bar{y}}:=0$ would not be restrictive since one could simply consider ${\widetilde{\varPhi }}:{\mathbb {X}}\rightrightarrows {\mathbb {Y}}$ given by $\widetilde{\varPhi }(x):=\varPhi (x)-{\bar{y}}$, $x\in {\mathbb {X}}$, in the case where ${\bar{y}}$ does not vanish. Optimality conditions and constraint qualifications for problems of this type can be found, e.g., in [36, 63, 65, 76]. A standard notion of stationarity, which applies to (P) and is based on the tools of limiting variational analysis, is the one of M-stationarity.

### Definition 4.1

A feasible point ${\bar{x}}\in {\mathcal {F}}$ of (P) is called *M-stationary* whenever there is a multiplier $\lambda \in {\mathbb {Y}}$ such that

$$\begin{aligned} 0\in \partial \varphi ({\bar{x}})+D^*\varPhi ({\bar{x}},{\bar{y}})(\lambda ). \end{aligned}$$

Later in Corollary 4.3, we will show that directional metric subregularity of $\varPhi$ serves as a constraint qualification for M-stationarity. In the following lemma, whose proof is analogous to the one of [14, Lemma 3.1], we point out that directional metric subregularity of $\varPhi$ implies that penalizing the constraint in (P) with the aid of the distance function yields a *directionally* exact penalty function.

### Lemma 4.1

Let ${\bar{x}}\in {\mathcal {F}}$ be a local minimizer of (P), and assume that $\varPhi$ is metrically subregular at $({\bar{x}},{\bar{y}})$ in direction $u\in {\mathbb {S}}_{{\mathbb {X}}}$. Then there are constants $\varepsilon >0$, $\delta >0$, and $C>0$ such that ${\bar{x}}$ is a global minimizer of

4.1 $$\begin{aligned} \min \limits _x\{ \varphi (x)+C{\text {dist}}({\bar{y}},\varPhi (x)) \,|\, x\in {\bar{x}}+{\mathbb {B}}_{\varepsilon ,\delta }(u) \}. \end{aligned}$$

Let us note that this result refines well-known findings about classical exact penalization in the presence of metric subregularity, see e.g. [27, 28, 56].

### Approaching mixed-order stationarity conditions

To start, let us introduce a quite general notion of critical directions associated with (P).

#### Definition 4.2

For some feasible point ${\bar{x}}\in {\mathcal {F}}$ and a pair $(\gamma _0,\gamma )\in {\mathbb {R}}\times {\mathbb {R}}$ such that $\gamma _0\ge 1$ as well as $\gamma \ge 1$, a direction $u\in {\mathbb {X}}$ is called *critical of order* $(\gamma _0,\gamma )$ for (P) at ${\bar{x}}$ whenever there are sequences $\{u_k\}_{k\in {\mathbb {N}}}\subset {\mathbb {X}}$, $\{\alpha _k\}_{k\in {\mathbb {N}}} \subset {\mathbb {R}}$, $\{v_k\}_{k\in {\mathbb {N}}}\subset {\mathbb {Y}}$, and $\{t_k\}_{k\in {\mathbb {N}}}\subset {\mathbb {R}}_+$ satisfying $u_k\rightarrow u$, $t_k\downarrow 0$, $\alpha _k \rightarrow 0$, $v_k\rightarrow 0$, and, for all $k\in {\mathbb {N}}$,

4.2 $$\begin{aligned} ({\bar{x}}+t_ku_k,\varphi ({\bar{x}}) + (t_k \left\| u_k\right\| )^{\gamma _0} \alpha _k) \in {\text {epi}}\varphi , \quad ({\bar{x}}+t_ku_k,{\bar{y}}+(t_k \left\| u_k\right\| )^{\gamma } v_k)\in {\text {gph}}\varPhi .\nonumber \\ \end{aligned}$$

If $(\gamma _0,\gamma ):=(1,1)$, we simply call *u* a *critical direction* for (P) at ${\bar{x}}$.

Clearly, $u:=0$ is critical of every order. Moreover, the set of all critical directions of any fixed order is a cone. The most standard case $(\gamma _0,\gamma ):=(1,1)$ corresponds to [36, Definition 5]. If $\varphi$ is directionally differentiable at ${\bar{x}}$, it is easily seen that $u\in {\mathbb {X}}$ is critical for (P) at ${\bar{x}}$ if and only if $\varphi '({\bar{x}};u)\le 0$ and $u\in \ker D\varPhi ({\bar{x}},{\bar{y}})$, see [72, Proposition 3.5] as well. Let us note that whenever ${\bar{x}}\in {\mathcal {F}}$ is a feasible point of (P) such that no critical direction for (P) at ${\bar{x}}$ exists, then ${\bar{x}}$ is a strict local minimizer of (P). Conversely, there may exist strict local minimizers of (P) such that a critical direction for (P) at this point exists.

While in this paper, we will not go beyond the case $\gamma _0:= 1$ (the case $\gamma _0:=2$ is briefly mentioned in Lemma 4.3), the situation $\gamma > 1$ (particularly $\gamma := 2$) will be very important. For $\gamma _0:= 1$ and arbitrary $\gamma \ge 1$, a critical direction $u \in {\mathbb {X}}$ still satisfies $\textrm{d}\varphi ({\bar{x}})(u) \le 0$ and $u\in \ker D_{\gamma }\varPhi ({\bar{x}},{\bar{y}})$, and the converse is valid whenever $\varphi$ is continuously differentiable at ${\bar{x}}$. In the next lemma, we show that if $\varPhi$ is metrically pseudo-subregular of order $\gamma$ at $({\bar{x}},{\bar{y}})$, then *u* is actually critical of order $(1,\gamma ')$ for each $\gamma ' \ge 1$.

#### Lemma 4.2

Fix a feasible point ${\bar{x}}\in {\mathcal {F}}$ of (P), $\gamma \ge 1$, and a critical direction $u \in {\mathbb {X}}$ of order $(1,\gamma )$ for (P) at ${\bar{x}}$. If $\varPhi$ is metrically pseudo-subregular of order $\gamma$ in direction *u* at $({\bar{x}},{\bar{y}})$, then *u* is critical of order $(1,\gamma ')$ for (P) at ${\bar{x}}$ for each $\gamma ' \ge 1$.

#### Proof

Let $\{u_k\}_{k\in {\mathbb {N}}}\subset {\mathbb {X}}$, $\{\alpha _k\}_{k\in {\mathbb {N}}} \subset {\mathbb {R}}$, $\{v_k\}_{k\in {\mathbb {N}}}\subset {\mathbb {Y}}$, and $\{t_k\}_{k\in {\mathbb {N}}}\subset {\mathbb {R}}_+$ be sequences satisfying $u_k\rightarrow u$, $t_k\downarrow 0$, $\alpha _k \rightarrow 0$, $v_k\rightarrow 0$, as well as (4.2) for all $k\in {\mathbb {N}}$. By metric pseudo-subregularity of order $\gamma$ of $\varPhi$ at $({\bar{x}},{\bar{y}})$, there is a constant $\kappa >0$ such that, for sufficiently large $k\in {\mathbb {N}}$, we get the existence of ${{\tilde{x}}}_k \in \varPhi ^{-1}({\bar{y}})$ with

$$\begin{aligned} \left\| {{\tilde{x}}}_k - ({\bar{x}}+t_ku_k)\right\| \le \kappa \frac{{\text {dist}}({\bar{y}},\varPhi ({\bar{x}}+t_ku_k))}{(t_k \left\| u_k\right\| )^{\gamma - 1}} \le \kappa \frac{(t_k \left\| u_k\right\| )^{\gamma } \left\| v_k\right\| }{(t_k \left\| u_k\right\| )^{\gamma - 1}} = \kappa t_k \left\| u_k\right\| \left\| v_k\right\| . \end{aligned}$$

Particularly, we find $\Vert ({{\tilde{x}}}_k - {\bar{x}})/t_k - u_k\Vert \rightarrow 0$ from $u_k\rightarrow u$ and $v_k \rightarrow 0$. Moreover, Lipschitzianity of $\varphi$ yields

$$\begin{aligned} \varphi ({{\tilde{x}}}_k)&\le \varphi ({{\tilde{x}}}_k) - \varphi ({\bar{x}} + t_k u_k) + \varphi ({\bar{x}}) + t_k \left\| u_k\right\| \alpha _k \\&\le L\kappa t_k \left\| u_k\right\| \left\| v_k\right\| + \varphi ({\bar{x}}) + t_k \left\| u_k\right\| \alpha _k = \varphi ({\bar{x}}) + t_k \left\| u_k\right\| (L\kappa \left\| v_k\right\| + \alpha _k) \end{aligned}$$

for some constant $L > 0$ and sufficiently large $k \in {\mathbb {N}}$. Thus, setting ${{\tilde{u}}}_k:= ({{\tilde{x}}}_k - {\bar{x}})/t_k$, ${\tilde{\alpha }}_k:= L\kappa \left\| v_k\right\| + \alpha _k$, and ${{\tilde{t}}}_k:= t_k$ for large enough $k \in {\mathbb {N}}$ yields ${{\tilde{u}}}_k\rightarrow u$, ${{\tilde{t}}}_k\downarrow 0$, ${\tilde{\alpha }}_k\rightarrow 0$, as well as

$$\begin{aligned} ({\bar{x}}+{{\tilde{t}}}_k{{\tilde{u}}}_k,\varphi ({\bar{x}}) + {{\tilde{t}}}_k \Vert {{\tilde{u}}}_k\Vert {\tilde{\alpha }}_k) \in {\text {epi}}\varphi , \qquad ({\bar{x}}+{{\tilde{t}}}_k{{\tilde{u}}}_k,{\bar{y}})\in {\text {gph}}\varPhi \end{aligned}$$

for large enough $k\in {\mathbb {N}}$, and so *u* is critical of order $(1,\gamma ')$ for (P) at ${\bar{x}}$ for each $\gamma '\ge 1$. $\square$

The following result, inspired by and based on [37, Proposition 2], provides an important interpretation of the notion from Definition 4.2 in terms of the so-called *epigraphical* map $M_0:{\mathbb {X}} \rightrightarrows {\mathbb {R}}$ associated with $\varphi$ and given by $M_0(x):= \varphi (x) + {\mathbb {R}}_+$, $x\in {\mathbb {X}}$. The proof follows simply from the fact that ${\text {gph}}M_0 = {\text {epi}}\varphi$ together with Remark 2.1.

#### Proposition 4.1

Given a feasible point ${\bar{x}}\in {\mathcal {F}}$ and a pair $(\gamma _0,\gamma )\in {\mathbb {R}}\times {\mathbb {R}}$ such that $\gamma _0\ge 1$ as well as $\gamma \ge 1$, a direction $u\in {\mathbb {S}}_{{\mathbb {X}}}$ is critical of order $(\gamma _0,\gamma )$ for (P) at ${\bar{x}}$ if and only if there exist sequences $\{u_k\}_{k\in {\mathbb {N}}}\subset {\mathbb {X}}$ and $\{t_k\}_{k\in {\mathbb {N}}}\subset {\mathbb {R}}_+$ such that $u_k \rightarrow u$, $t_k\downarrow 0$, and

4.3 $$\begin{aligned} \frac{{\text {dist}}(\varphi ({\bar{x}}),M_0({\bar{x}} + t_k u_k))}{(t_k \left\| u_k\right\| )^{\gamma _0}} \rightarrow 0, \qquad \frac{{\text {dist}}({\bar{y}},\varPhi ({\bar{x}} + t_k u_k))}{(t_k \left\| u_k\right\| )^\gamma } \rightarrow 0. \end{aligned}$$

Moreover, if $\gamma _0 = \gamma$, this is further equivalent to the condition

$$\begin{aligned} u\in \ker D_{\gamma }M({\bar{x}}, (\varphi ({\bar{x}}), {\bar{y}})) \end{aligned}$$

for the mapping $M:{\mathbb {X}} \rightrightarrows {\mathbb {R}}\times {\mathbb {Y}}$ given by $M(x):=M_0(x)\times \varPhi (x)$, $x\in {\mathbb {X}}$.

Interestingly, Gfrerer used the conditions (4.3) as a basis of his optimality conditions in [37, Proposition 2], but he did not notice, or at least did not mention, that these conditions actually provide a natural extension of his own notion of a critical direction from [36, Definition 5]. This observation enables us to formulate an extension of the common pattern “for every critical direction there is a multiplier satisfying an FJ-type optimality condition” in Corollary 4.1 below.

#### Remark 4.1

Gfrerer recognized the importance of considering Cartesian product mappings $M:{\mathbb {X}}\rightrightarrows {\mathbb {Y}}_0\times {\mathbb {Y}}_1\times \ldots \times {\mathbb {Y}}_s$, given by

$$\begin{aligned} \forall x\in {\mathbb {X}}:\quad M(x):=M_0(x)\times M_1(x)\times \ldots \times M_s(x) \end{aligned}$$

for the component maps $M_i:{\mathbb {X}}\rightrightarrows {\mathbb {Y}}_i$, $i=0,1,\ldots ,s$, and Euclidean spaces ${\mathbb {Y}}_0,{\mathbb {Y}}_1,\ldots ,{\mathbb {Y}}_s$, and to allow different orders $\gamma _i\ge 1$ of pseudo-(sub)regularity of these component mappings, see [37, Definition 1]. In the same manner, he defined his pseudo-coderivative [37, Definition 2]. This was essential for his approach to optimality conditions. For brevity of presentation, we avoid these definitions and bypass explicitly using these notions by applying [37, Proposition 2] in combination with the sufficient conditions for pseudo-subregularity from [37, Theorem 1(2)] to prove Corollary 4.1.

#### Corollary 4.1

Let ${\bar{x}}\in {\mathcal {F}}$ be a local minimizer of (P) and let $u\in {\mathbb {S}}_{{\mathbb {X}}}$ be a critical direction of order $(1,\gamma )$ for (P) at ${\bar{x}}$ with $\gamma \ge 1$. Then there exist multipliers $(0,0) \ne (\alpha ^*, \lambda ) \in {\mathbb {R}}_+ \times {\mathbb {Y}}$ satisfying

$$\begin{aligned} 0 \in \alpha ^* \partial \varphi ({\bar{x}};u) + D^*_{\gamma }\varPhi (({\bar{x}}, {\bar{y}});(u,0))(\lambda ). \end{aligned}$$

If the sufficient condition (2.12) for metric pseudo-subregularity of order $\gamma$ of $\varPhi$ in direction *u* at $({\bar{x}}, {\bar{y}})$ holds, then the above condition holds with $\alpha ^*:= 1$.

#### Proof

Applying Proposition 4.1 and then [37, Proposition 2 and Theorem 1(2)] yields an element $z^* \in {\mathbb {X}}$ and sequences $\{t_k\}_{k\in {\mathbb {N}}}\subset {\mathbb {R}}_+$, $\{u_k\}_{k\in {\mathbb {N}}},\{x_k^*\}_{k\in {\mathbb {N}}}\subset {\mathbb {X}}$, $\{\alpha _k\}_{k\in {\mathbb {N}}},\{\alpha _k^*\}_{k\in {\mathbb {N}}}\subset {\mathbb {R}}$, and $\{v_k\}_{k\in {\mathbb {N}}},\{\lambda _k\}_{k\in {\mathbb {N}}}\subset {\mathbb {Y}}$ satisfying (among other things) $t_k\downarrow 0$, $u_k\rightarrow u$, $\alpha _k\rightarrow 0$, $t_k^{1-\gamma }v_k\rightarrow 0$, as well as $x_k^*\rightarrow 0$, such that, for each $k\in {\mathbb {N}}$, $\left\| (\alpha _k^*,\lambda _k)\right\| =1$ and

4.4 $$\begin{aligned} (x_k^*,-\alpha _k^*, -\lambda _k/(t_k\left\| u_k\right\| )^{\gamma -1}) \in \widehat{{\mathcal {N}}}_{{\text {gph}}M^{z^*}}({\bar{x}} +t_ku_k,\varphi ({\bar{x}}) + t_k \alpha _k,{\bar{y}} + t_k^{\gamma } {{\tilde{v}}}_k),\qquad \end{aligned}$$

where we used ${{\tilde{v}}}_k:=t_k^{1-\gamma }v_k$ as well as the mapping $M^{z^*}:{\mathbb {X}} \rightrightarrows {\mathbb {R}}\times {\mathbb {Y}}$ defined by $M^{z^*}(x):=M^{z^*}_0(x)\times \varPhi (x)$, $x\in {\mathbb {X}}$, with the perturbed epigraphical mapping $M^{z^*}_0:{\mathbb {X}}\rightrightarrows {\mathbb {R}}$ given by

$$\begin{aligned} \forall x\in {\mathbb {X}}:\quad M^{z^*}_0(x):= \varphi (x) + \vert \langle z^*, x - {\bar{x}}\rangle \vert ^{3} + {\mathbb {R}}_+. \end{aligned}$$

Note that we have ${\text {gph}}M^{z^*} = \big ( {\text {gph}}M^{z^*}_0 \times {\mathbb {Y}} \big ) \cap {\mathcal {P}}({\text {gph}}\varPhi \times {\mathbb {R}})$, where the permutation mapping ${\mathcal {P}}:{\mathbb {X}} \times {\mathbb {Y}} \times {\mathbb {R}}\rightarrow {\mathbb {X}} \times {\mathbb {R}}\times {\mathbb {Y}}$ just swaps the last two components. After replacing the regular by the larger limiting normal cone in (4.4) and noting that $x\mapsto \varphi (x)+|\langle z^*, x-{\bar{x}}\rangle |^3$ is locally Lipschitzian, we can apply the intersection rule for limiting normals from [71, Theorem 6.42]. The latter yields, for each $k\in {\mathbb {N}}$, $x_{k,1}^*,x_{k,2}^*\in {\mathbb {X}}$ with $x_k^* = x_{k,1}^* + x_{k,2}^*$ and

$$\begin{aligned} (x_{k,1}^*,-\alpha _k^*)&\in {\mathcal {N}}_{{\text {gph}}M^{z^*}_0 }({\bar{x}} +t_ku_k,\varphi ({\bar{x}}) + t_k \alpha _k),\\ (x_{k,2}^*,-\lambda _k/(t_k\left\| u_k\right\| )^{\gamma -1})&\in {\mathcal {N}}_{{\text {gph}}\varPhi }({\bar{x}} +t_ku_k,{\bar{y}} + t_k^{\gamma } {{\tilde{v}}}_k). \end{aligned}$$

Now, local Lipschitzness of $x\mapsto \varphi (x)+|\langle z^*, x-{\bar{x}}\rangle |^3$ together with boundedness of $\{\alpha _k^*\}_{k\in {\mathbb {N}}}$ implies boundedness of $\{x_{k,1}^*\}_{k\in {\mathbb {N}}}$. This, in turn, gives boundedness of $\{x_{k,2}^*\}_{k\in {\mathbb {N}}}$. Since $\{\lambda _k\}_{k\in {\mathbb {N}}}$ is also bounded, taking the limit along a suitable subsequence yields some $x^*\in {\mathbb {X}}$, $\alpha ^*\in {\mathbb {R}}$, and $\lambda \in {\mathbb {Y}}$ satisfying $(\alpha ^*,\lambda )\ne (0,0)$ as well as

$$\begin{aligned} x^* \in D^* M^{z^*}_0 (({\bar{x}}, \varphi ({\bar{x}}));(u,0))(\alpha ^*), \quad -x^* \in D^*_{\gamma }\varPhi (({\bar{x}}, {\bar{y}});(u,0))(\lambda ). \end{aligned}$$

Here, we used the robustness of the directional limiting coderivative, see Lemma 2.1, as well as Lemma 2.8. Taking into account that $x\mapsto \vert \langle z^*, x - {\bar{x}}\rangle \vert ^{3}$ is smooth with its gradient vanishing at ${\bar{x}}$ and using Lemma 3.1 (c) as well as [18, Proposition 5.1], we get $\alpha ^* \ge 0$ and $D^* M^{z^*}_0 (({\bar{x}}, \varphi ({\bar{x}}));(u,0))(\alpha ^*) \subset \alpha ^* \partial \varphi ({\bar{x}};u)$. This proves the first statement.

Finally, (2.12) clearly implies $\alpha ^* > 0$, and by rescaling, we can set $\alpha ^*:= 1$. $\square$

### Mixed-order and asymptotic stationarity conditions

The following result provides asymptotic necessary optimality conditions for (P) which hold in the absence of constraint qualifications. The derived conditions depend on a certain *order* $\gamma \ge 1$. Furthermore, our result specifies how the asymptotic case (d) can be ruled out by metric pseudo-subregularity of $\varPhi$ of order $\gamma$ at the reference point.

#### Theorem 4.1

Let ${\bar{x}}\in {\mathcal {F}}$ be a local minimizer of (P) and consider $\gamma \ge 1$. Then one of the following conditions holds.

- The point ${\bar{x}}$ is M-stationary for (P).
- There exists a critical direction $u\in {\mathbb {S}}_{{\mathbb {X}}}$ for (P) at ${\bar{x}}$ such that
  4.5 $$\begin{aligned} 0\in \partial \varphi ({\bar{x}};u)+{\widetilde{D}}^*_\gamma \varPhi (({\bar{x}},{\bar{y}});(u,0))(0). \end{aligned}$$
- There exist a critical direction $u\in {\mathbb {S}}_{{\mathbb {X}}}$ for (P) at ${\bar{x}}$, a nonvanishing multiplier $\lambda \in {\mathbb {Y}}$, and $\alpha \ge 0$ such that, for $v:= \alpha \lambda$, we have
  4.6 $$\begin{aligned} 0 \in \partial \varphi ({\bar{x}};u) + D^*_{\gamma } \varPhi (({\bar{x}},{\bar{y}});(u,v))(\lambda ). \end{aligned}$$
- There exist a critical direction $u\in {\mathbb {S}}_{{\mathbb {X}}}$ of order $(\gamma _0,\gamma )$ for (P) at ${\bar{x}}$ for each $\gamma _0\ge 1$, some $y^*\in {\mathbb {Y}}$, and sequences $\{x_k\}_{k\in {\mathbb {N}}},\{\eta _k\}_{k\in {\mathbb {N}}}\subset {\mathbb {X}}$ as well as $\{y_k\}_{k\in {\mathbb {N}}}\subset {\mathbb {Y}}$ such that $x_k\notin \varPhi ^{-1}({\bar{y}})$ and $y_k\ne {\bar{y}}$ for all $k\in {\mathbb {N}}$, satisfying the convergence properties
  $$\begin{aligned} \begin{aligned} x_k&\rightarrow {\bar{x}},&\qquad y_k&\rightarrow {\bar{y}},&\qquad \eta _k&\rightarrow 0,&\qquad \frac{x_k-{\bar{x}}}{\Vert x_k-{\bar{x}}\Vert }&\rightarrow u,&\\ v_k^\gamma&\rightarrow 0,&\qquad \Vert \lambda _k^\gamma \Vert&\rightarrow \infty ,&\qquad \Vert v_k^\gamma \Vert \lambda _k^\gamma&\rightarrow y^*,{} & {} {}&\end{aligned} \end{aligned}$$
  where we used
  4.7 $$\begin{aligned} \forall k\in {\mathbb {N}}:\quad v^\gamma _k:= \frac{y_k-{\bar{y}}}{\left\| x_k-{\bar{x}}\right\| ^\gamma },\qquad \lambda _k^\gamma := k\Vert x_k-{\bar{x}}\Vert ^{\gamma -1}(y_k-{\bar{y}}), \end{aligned}$$
  as well as
  4.8 $$\begin{aligned} \forall k\in {\mathbb {N}}:\quad \eta _k\in \partial \varphi (x_k)+D^*\varPhi (x_k,y_k)\left( \frac{\lambda _k^\gamma }{\left\| x_k - {\bar{x}}\right\| ^{\gamma -1}}\right) . \end{aligned}$$

Moreover, if $\varPhi$ is metrically pseudo-subregular of order $\gamma$ at $({\bar{x}}, {\bar{y}})$ in each direction $u\in \ker D\varPhi ({\bar{x}},{\bar{y}})\cap {\mathbb {S}}_{{\mathbb {X}}}$, ${\bar{x}}$ satisfies one of the alternatives (a), (b), or (c).

#### Proof

Let $\varepsilon >0$ be chosen such that $\varphi (x)\ge \varphi ({\bar{x}})$ holds for all $x\in {\mathcal {F}}\cap {\mathbb {B}}_\varepsilon ({\bar{x}})$ and, for each $k\in {\mathbb {N}}$, consider the optimization problem 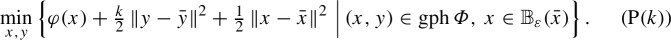 For each $k\in {\mathbb {N}}$, the objective function of (P(*k*)) is bounded from below, continuous on the closed feasible set of this problem, and coercive in the variable *y*, so (P(*k*)) possesses a global minimizer $(x_k,y_k)\in {\mathbb {X}}\times {\mathbb {Y}}$. By feasibility of $({\bar{x}},{\bar{y}})$ for (P(*k*)), we find

4.9 $$\begin{aligned} \forall k\in {\mathbb {N}}:\quad \varphi (x_k)+\tfrac{k}{2}\left\| y_k-{\bar{y}}\right\| ^2+\tfrac{1}{2}\left\| x_k-{\bar{x}}\right\| ^2\le \varphi ({\bar{x}}). \end{aligned}$$

By boundedness of $\{x_k\}_{k\in {\mathbb {N}}}\subset {\mathbb {B}}_\varepsilon ({\bar{x}})$, we may assume $x_k\rightarrow {{\tilde{x}}}$ for some ${{\tilde{x}}}\in {\mathbb {B}}_\varepsilon ({\bar{x}})$. Observing that $\{\varphi (x_k)\}_{k\in {\mathbb {N}}}$ is bounded by continuity of $\varphi$, $y_k\rightarrow {\bar{y}}$ easily follows from (4.9). Furthermore, the closedness of ${\text {gph}}\varPhi$ guarantees $({{\tilde{x}}},{\bar{y}})\in {\text {gph}}\varPhi$, i.e., ${{\tilde{x}}}\in {\mathcal {F}}\cap {\mathbb {B}}_\varepsilon ({\bar{x}})$ leading to $\varphi ({\bar{x}})\le \varphi ({{\tilde{x}}})$. From (4.9), we find

$$\begin{aligned} \varphi ({\bar{x}}) \le \varphi ({{\tilde{x}}}) \le \varphi ({{\tilde{x}}})+\tfrac{1}{2}\left\| {{\tilde{x}}}-{\bar{x}}\right\| ^2 = \lim \limits _{k\rightarrow \infty }\left( \varphi (x_k)+\tfrac{1}{2}\left\| x_k-{\bar{x}}\right\| ^2\right) \le \varphi ({\bar{x}}), \end{aligned}$$

and ${{\tilde{x}}}={\bar{x}}$ follows. Thus, we have $x_k\rightarrow {\bar{x}}$.

Let us assume that there is some $k_0\in {\mathbb {N}}$ such that $x_{k_0}$ is feasible to (P). By (4.9), we find

$$\begin{aligned}&\varphi ({\bar{x}}) +\tfrac{k_0}{2}\Vert {y_{k_0}-{\bar{y}}}\Vert ^2+\tfrac{1}{2}\Vert {x_{k_0}-{\bar{x}}}\Vert ^2 \\&\qquad \le \varphi (x_{k_0})+\tfrac{k_0}{2}\Vert {y_{k_0}-{\bar{y}}}\Vert ^2+\tfrac{1}{2}\Vert {x_{k_0}-{\bar{x}}}\Vert ^2 \le \varphi ({\bar{x}}), \end{aligned}$$

i.e., $x_{k_0}={\bar{x}}$ and $y_{k_0}={\bar{y}}$. Applying [66, Theorem 6.1], the subdifferential sum rule [66, Theorem 2.19], and the definition of the limiting coderivative to find stationarity conditions of (P(*k*)) at $({\bar{x}},{\bar{y}})$ yields $0\in \partial \varphi ({\bar{x}})+D^*\varPhi ({\bar{x}},{\bar{y}})(0)$, which is covered by (a).

Thus, we may assume that $x_k\notin {\mathcal {F}}=\varPhi ^{-1}({\bar{y}})$ holds for all $k\in {\mathbb {N}}$. Particularly, $x_k\ne {\bar{x}}$ and $y_k\ne {\bar{y}}$ is valid for all $k\in {\mathbb {N}}$ in this situation. Assume without loss of generality that $\{x_k\}_{k\in {\mathbb {N}}}$ belongs to the interior of ${\mathbb {B}}_\varepsilon ({\bar{x}})$.

We can apply Fermat’s rule, see [66, Proposition 1.30 (i)], the semi-Lipschitzian sum rule for limiting subgradients from [66, Corollary 2.20], and the definition of the limiting coderivative in order to find

4.10 $$\begin{aligned} \forall k\in {\mathbb {N}}:\quad {\bar{x}}-x_k\in \partial \varphi (x_k)+D^*\varPhi (x_k,y_k)(k(y_k-{\bar{y}})). \end{aligned}$$

Setting $\eta _k:={\bar{x}}-x_k$ for each $k\in {\mathbb {N}}$, we find $\eta _k\rightarrow 0$. Since $\{(x_k - {\bar{x}})/\Vert x_k - {\bar{x}}\Vert \}_{k\in {\mathbb {N}}}\subset {\mathbb {S}}_{{\mathbb {X}}}$, we may assume $(x_k - {\bar{x}})/\Vert x_k - {\bar{x}}\Vert \rightarrow u$ for some $u\in {\mathbb {S}}_{{\mathbb {X}}}$.

Next, we claim that $\{y_k^*\}_{k\in {\mathbb {N}}}\subset {\mathbb {Y}}$, given by $y_k^*:= k(y_k-{\bar{y}})\left\| y_k-{\bar{y}}\right\| /\left\| x_k - {\bar{x}}\right\|$ for each $k\in {\mathbb {N}}$, is bounded. Rearranging (4.9), leaving a nonnegative term away, and division by $\Vert x_k-{\bar{x}}\Vert$ give us

4.11 $$\begin{aligned} \forall k\in {\mathbb {N}}:\quad \frac{\varphi (x_k)-\varphi ({\bar{x}})}{\Vert x_k-{\bar{x}}\Vert } + \frac{k}{2}\frac{\Vert y_k-{\bar{y}}\Vert ^2}{\Vert x_k-{\bar{x}}\Vert } \le 0. \end{aligned}$$

Lipschitzianity of $\varphi$ yields boundedness of the first fraction, so that the sequence $\{k\Vert y_k-{\bar{y}}\Vert ^2/\Vert x_k-{\bar{x}}\Vert \}_{k\in {\mathbb {N}}}$ must be bounded and, consequently, $\{y_k^*\}_{k\in {\mathbb {N}}}$ as well. Thus, we may assume $y_k^*\rightarrow y^*$ for some $y^*\in {\mathbb {Y}}$.

Suppose that $\{(y_k-{\bar{y}})/\Vert x_k-{\bar{x}}\Vert \}_{k\in {\mathbb {N}}}$ does not converge to zero. This, along a subsequence (without relabeling), yields boundedness of the sequence $\{k(y_k-{\bar{y}})\}_{k\in {\mathbb {N}}}$, and taking the limit $k\rightarrow \infty$ in (4.10) along yet another subsequence while respecting robustness of the limiting subdifferential and the limiting coderivative yields (a).

Thus, we may assume $(y_k-{\bar{y}})/\left\| x_k-{\bar{x}}\right\| \rightarrow 0$ for the remainder of the proof. Observe that we have

$$\begin{aligned} ({\bar{x}}+ \left\| x_k - {\bar{x}}\right\| (x_k - {\bar{x}})/\left\| x_k - {\bar{x}}\right\| ,{\bar{y}}+\left\| x_k - {\bar{x}}\right\| (y_k - {\bar{y}})/\left\| x_k - {\bar{x}}\right\| ) \in {\text {gph}}\varPhi \end{aligned}$$

for all $k\in {\mathbb {N}}$. Additionally, (4.11) yields

$$\begin{aligned} \varphi ({\bar{x}}+ \left\| x_k - {\bar{x}}\right\| (x_k - {\bar{x}})/\left\| x_k - {\bar{x}}\right\| )-\varphi ({\bar{x}}) \le 0, \end{aligned}$$

so *u* is a critical direction of order $(\gamma _0, 1)$ for each $\gamma _0 \ge 1$ for (P) at ${\bar{x}}$.

In the remainder of the proof, we are going to exploit the sequences $\{v_k^\gamma \}_{k\in {\mathbb {N}}},\{\lambda _k^\gamma \}_{k\in {\mathbb {N}}} \subset {\mathbb {Y}}$ given as in (4.7). Observe that $y_k^* = \Vert v^\gamma _k\Vert \lambda _k^\gamma$, i.e., $\lambda _k^\gamma = y_k^*\left\| x_k - {\bar{x}}\right\| ^\gamma /\left\| y_k - {\bar{y}}\right\|$ is valid for each $k\in {\mathbb {N}}$. Note that the optimality condition (4.10) can be rewritten as

4.12 $$\begin{aligned} \forall k\in {\mathbb {N}}:\quad \eta _k \in \partial \varphi (x_k)+D^*\varPhi (x_k,{\bar{y}}+ \left\| x_k - {\bar{x}}\right\| ^\gamma v^\gamma _k)\left( \frac{\lambda _k^\gamma }{\left\| x_k - {\bar{x}}\right\| ^{\gamma -1}}\right) . \nonumber \\ \end{aligned}$$

Now, we need to distinguish three options.

Let us assume that $\lambda _k^\gamma \rightarrow 0$. Using $t_k:=\left\| x_k-{\bar{x}}\right\|$, we can reformulate (4.12) as

$$\begin{aligned} \forall k\in {\mathbb {N}}:\quad \eta _k \in \partial \varphi (x_k) + D^*\varPhi \left( {\bar{x}}+t_k\frac{x_k-{\bar{x}}}{\left\| x_k-{\bar{x}}\right\| },{\bar{y}} +t_k\frac{y_k-{\bar{y}}}{\left\| x_k-{\bar{x}}\right\| }\right) \left( \frac{\lambda _k^\gamma }{t_k^{\gamma -1}}\right) . \end{aligned}$$

Taking the limit $k\rightarrow \infty$ while respecting robustness of the directional limiting subdifferential as well as Lemma 2.8 yields (b) since $(y_k-{\bar{y}})/\left\| x_k-{\bar{x}}\right\| \rightarrow 0$ and *u* has already been shown to be critical for (P) at ${\bar{x}}$.

If $\{\lambda _k^\gamma \}_{k\in {\mathbb {N}}}$ remains bounded but, along a subsequence (without relabeling), stays away from zero, we also get boundedness of $\{v_k^{\gamma }\}_{k\in {\mathbb {N}}}$ from boundedness of $\{y_k^*\}_{k\in {\mathbb {N}}}$, and taking the limit along a convergent subsequence (without relabeling) in (4.12) while respecting robustness of the directional limiting subdifferential and Lemma 2.8 yields precisely (4.6), where $\lambda , v \in {\mathbb {Y}}$ with $\lambda \ne 0$ satisfy $\lambda _k^{\gamma }\rightarrow \lambda$ and $v_k^{\gamma }\rightarrow v$, respectively, and using $\alpha _k:=(k \left\| x_k - {\bar{x}}\right\| ^{2\gamma -1})^{-1}$ for all $k\in {\mathbb {N}}$ as well as (4.7), we find $v_k^{\gamma } = \alpha _k \lambda _k^{\gamma }$ for all $k\in {\mathbb {N}}$, $\alpha _k\rightarrow \left\| v\right\| /\left\| \lambda \right\| =:\alpha$, and $v=\alpha \lambda$. Criticality of *u* for (P) at ${\bar{x}}$ has been shown above. Thus, situation (c) has been verified.

If $\{\lambda _k^\gamma \}_{k\in {\mathbb {N}}}$ is not bounded, we pass to a subsequence (without relabeling) such that $\Vert \lambda _k^{\gamma }\Vert \rightarrow \infty$ and so we also get $v_k^{\gamma } \rightarrow 0$ along this subsequence by boundedness of $\{y_k^*\}_{k\in {\mathbb {N}}}$. This means that *u* is actually critical of order $(\gamma _0,\gamma )$ for (P) at ${\bar{x}}$ and so all conditions stated in (d) have been verified since (4.8) follows from (4.12).

Finally, let us argue that option (d) can be avoided, i.e., that the sequence $\{\lambda _k^\gamma \}_{k\in {\mathbb {N}}}$ from above remains bounded if we assume that $\varPhi$ is metrically pseudo-subregular of order $\gamma$ in direction *u* at $({\bar{x}},{\bar{y}})$. By boundedness of $\{y_k^*\}_{k\in {\mathbb {N}}}$, we immediately obtain the boundedness of $\{\lambda _k^\gamma \}_{k\in {\mathbb {N}}}$ unless we have $v_k^\gamma \rightarrow 0$. Thus, let us assume the latter. By metric pseudo-subregularity of $\varPhi$, there is a constant $\kappa >0$ such that, for sufficiently large $k\in {\mathbb {N}}$, we get the existence of ${{\tilde{x}}}_k \in \varPhi ^{-1}({\bar{y}})$ with

4.13 $$\begin{aligned} \left\| x_k - {{\tilde{x}}}_k\right\| \le \kappa \frac{{\text {dist}}({\bar{y}},\varPhi (x_k))}{\left\| x_k-{\bar{x}}\right\| ^{\gamma - 1}} \le \kappa \frac{\left\| y_k - {\bar{y}}\right\| }{\left\| x_k-{\bar{x}}\right\| ^{\gamma - 1}} = \kappa \left\| x_k-{\bar{x}}\right\| \Vert v_k^\gamma \Vert . \end{aligned}$$

Particularly, we find $\Vert x_k-{{\tilde{x}}}_k\Vert \rightarrow 0$ from $v_k^\gamma \rightarrow 0$, and ${{\tilde{x}}}_k\rightarrow {\bar{x}}$ follows. Since $(x_k,y_k)$ is a global minimizer of (P(*k*)), we get

$$\begin{aligned} \frac{\varphi (x_k)-\varphi ({{\tilde{x}}}_k)}{\left\| x_k-{\bar{x}}\right\| } + \frac{k}{2}\frac{\left\| y_k-{\bar{y}}\right\| ^2}{\left\| x_k-{\bar{x}}\right\| } + \frac{1}{2}\frac{\left\| x_k-{\bar{x}}\right\| ^2 - \left\| {{\tilde{x}}}_k-{\bar{x}}\right\| ^2}{\left\| x_k-{\bar{x}}\right\| } \le 0 \end{aligned}$$

for all sufficiently large $k\in {\mathbb {N}}$. Due to $v_k^\gamma \ne 0$ for all $k \in {\mathbb {N}}$, rearranging the above estimate and using (4.7) as well as (4.13) yield

$$\begin{aligned} \Vert \lambda _k^{\gamma }\Vert = \frac{k \left\| y_k-{\bar{y}}\right\| ^2}{\left\| x_k-{\bar{x}}\right\| \Vert v_k^{\gamma }\Vert } \le \frac{2\kappa \vert \varphi (x_k)-\varphi ({{\tilde{x}}}_k) \vert }{\left\| x_k - {{\tilde{x}}}_k\right\| } + \frac{\kappa \big \vert \left\| x_k-{\bar{x}}\right\| ^2 - \left\| {{\tilde{x}}}_k-{\bar{x}}\right\| ^2\big \vert }{\left\| x_k - {{\tilde{x}}}_k\right\| }. \end{aligned}$$

Boundedness of $\{\lambda _k^\gamma \}_{k\in {\mathbb {N}}}$ thus follows from Lipschitzianity of $\varphi$ and the estimate

$$\begin{aligned} \big \vert \left\| x_k-{\bar{x}}\right\| ^2 - \left\| {{\tilde{x}}}_k-{\bar{x}}\right\| ^2\big \vert \le \left\| x_k - {{\tilde{x}}}_k\right\| \big (\left\| {{\tilde{x}}}_k-{\bar{x}}\right\| + \left\| x_k-{\bar{x}}\right\| \big ). \end{aligned}$$

This completes the proof. $\square$

Let us note that for the price of some more technicalities in the proof, involving the fuzzy sum rule for the regular subdifferential, see e.g. [66, Exercise 2.26], it is possible to formulate statement (d) in terms of the regular tools of variational analysis, see [21, Theorem 4.3] which is a preprint version of this paper. This more involved approach then allows for an easier comparison to available results in the literature which are partially stated in infinite dimensions, see e.g. [37], where the limiting tools are of limited use and sequential characterizations in terms of the regular tools are, thus, preferred. However, for our purposes, the way Theorem 4.1 has been formulated will be enough to proceed.

In the rest of this subsection, we discuss some applications of Theorem 4.1, which are then further developed in the rest of the paper. First, we focus on mixed-order stationarity conditions, involving first-order generalized derivatives of the objective function and pseudo-coderivatives of order $\gamma$, and enhance the result of Corollary 4.1 as follows.

#### Corollary 4.2

Let ${\bar{x}}\in {\mathcal {F}}$ be a local minimizer of (P) and consider $\gamma \ge 1$. Then the following assertions hold.

- If $\varPhi$ is metrically pseudo-subregular of order $\gamma$ at $({\bar{x}},{\bar{y}})$ in each unit direction, then one of the alternatives (a), (b), or (c) of Theorem 4.1 holds.
- If there are no critical directions of order $(1,\gamma )$ for (P) at ${\bar{x}}$, then one of the alternatives (a), (b), or (c) from Theorem 4.1 is valid. If there exists a critical directions $u\in {\mathbb {S}}_{{\mathbb {X}}}$ of order $(1,\gamma )$ for (P) at ${\bar{x}}$ satisfying (2.12), then even
  4.14 $$\begin{aligned} 0 \in \partial \varphi ({\bar{x}};u) + D^*_{\gamma } \varPhi (({\bar{x}},{\bar{y}});(u,0))(\lambda ) \end{aligned}$$
  holds for some $\lambda \in {\mathbb {Y}}$.

#### Proof

The first assertion follows directly from Theorem 4.1. Let us now prove the second assertion. Theorem 4.1 says that either one of the alternatives (a), (b), or (c) holds, or there exists a critical direction $u\in {\mathbb {S}}_{{\mathbb {X}}}$ of order $(1,\gamma )$ for (P) at ${\bar{x}}$ (with certain properties). If among these critical directions, there is one that satisfies (2.12), Corollary 4.1 yields (4.14). $\square$

We conjecture that the sufficient condition (2.12) can be weakened to just pseudo-subregularity of $\varPhi$ in Corollary 4.2 (b). However, it would require a different proof to show this, so we will not explore this option. For $\gamma :=1$, such a result is known to hold, see [36, Theorem 7].

Note that (4.14) is covered by the alternative (b) (if $\lambda = 0$, see (2.7)) or (c) (if $\lambda \ne 0$) of Theorem 4.1. Hence, the optimality conditions from Corollary 4.2 give either M-stationarity of the underlying local minimizer or validity of alternative (b) or (c) of Theorem 4.1 for some critical direction (of order (1, 1) or $(1,\gamma )$).

#### Remark 4.2

Corollary 4.2 offers two distinct paths to an optimality condition of type “M-stationarity or (4.14)”, both with some advantages and disadvantages.

- Assuming pseudo-subregularity in *each* unit direction yields this type of condition by ruling out the alternative (d) of Theorem 4.1. However, this can sometimes be an undesirable type of assumption as pointed out in Remark 3.5.
- The refined assumptions in Corollary 4.2 (b) are clearly milder, but they depend on a critical direction (of order $(1,\gamma )$), which in turn depends also on the objective function, not just on $\varPhi$. These assumptions do not rule out the alternative (d). Instead, they just secure that (a), (b), or (c) from Theorem 4.1 holds.

These two types of assumptions will be prevalent throughout this section.

Recall that all the assumptions in Corollary 4.2 become less restrictive as $\gamma$ increases, see Sect. 2.3.4 as well. On the contrary, with increasing $\gamma$, the involved pseudo-coderivatives become more difficult to handle which, exemplary, can be seen for constraint mappings when comparing the cases $\gamma :=1$ and $\gamma :=2$ from Sect. 3. In this regard, in Corollary 4.2, $\gamma$ should be chosen as small as possible such that the exploited qualification condition is valid.

In Sect. 4.3, we work out the conditions from Corollary 4.2 for $\gamma :=2$ in the setting where $\varPhi$ is a constraint mapping as the appearing pseudo-coderivatives actually can be computed, see Sect. 3, and, hence, we obtain conditions in terms of initial problem data. In Sect. 4.4, we further apply these results to two specific problem classes and compare them with similar results based on 2-regularity.

Theorem 4.1 also opens a way to the identification of new conditions which guarantee that local minimizers of (P) are M-stationary. One of the most prominent conditions that implies this is metric subregularity, and the corresponding result, which we state next, can be obtained simply by setting $\gamma := 1$ in Corollary 4.2, taking also into account [36, Theorem 7]. For us, this result serves as a basis for comparison. Later on, we will derive new conditions, which are independent of (directional) metric subregularity, but which are milder than various known sufficient conditions for metric subregularity.

#### Corollary 4.3

A local minimizer ${\bar{x}}\in {\mathcal {F}}$ of (P) is M-stationary if one of the following conditions holds.

- The mapping $\varPhi$ is metrically subregular at $({\bar{x}},{\bar{y}})$ in each unit direction.
- There are no critical directions for (P) at ${\bar{x}}$, or there is a critical direction $u\in {\mathbb {S}}_{{\mathbb {X}}}$ for (P) at ${\bar{x}}$ and $\varPhi$ is metrically subregular at $({\bar{x}},{\bar{y}})$ in direction *u*, in which case there is a multiplier $\lambda \in {\mathbb {Y}}$ such that
  $$\begin{aligned} 0\in \partial \varphi ({\bar{x}};u)+D^*\varPhi (({\bar{x}},{\bar{y}});(u,0))(\lambda ). \end{aligned}$$

Let us now discuss two novel approaches to M-stationarity. The first approach corresponds to using Corollary 4.2 with $\gamma > 1$ and then making sure that the derived mixed-order conditions in terms of pseudo-coderivatives actually yield M-stationarity. To formalize the idea, we introduce the following assumption.

#### Assumption 4.1

Given $u \in {\mathbb {S}}_{{\mathbb {X}}}$ and $\gamma \ge 1$, we say that $A^\gamma (u)$ holds if (2.12) is satisfied and

4.15 $$\begin{aligned} {\widetilde{D}}^*_\gamma \varPhi (({\bar{x}},{\bar{y}});(u,0))(0) \cup \bigcup _{w\in {\mathbb {S}}_{{\mathbb {Y}}}} D^*_\gamma \varPhi (({\bar{x}},{\bar{y}});(u,\alpha w))(\beta w) \subset {\text {Im}}D^*\varPhi ({\bar{x}},{\bar{y}})\nonumber \\ \end{aligned}$$

is valid for all $\alpha ,\beta \ge 0$.

Let us mention that

4.16 $$\begin{aligned} {\text {Im}}{{\widetilde{D}}}^*_\gamma \varPhi (({\bar{x}},{\bar{y}});(u,0)) \subset {\text {Im}}D^*\varPhi ({\bar{x}},{\bar{y}}) \end{aligned}$$

is a sufficient condition for (4.15) due to (2.7). Assumption $A^\gamma (u)$ leads to the problem of how to compute or estimate the appearing pseudo-coderivatives. As mentioned above, for $\gamma :=2$ and in the setting where $\varPhi$ is a constraint mapping, these objects can be computed and assumption $A^\gamma (u)$ can be made explicit. We discuss this case in detail in Sect. 5.3.2, where we show that $A^2(u)$ is (strictly) weaker than FOSCMS(*u*) as well as its refinement SOSCMS(*u*) in the polyhedral case. Here, we just explain how $A^\gamma (u)$ can be used to secure M-stationarity and how to compare it with sufficient conditions for metric subregularity.

To proceed, let ${\bar{x}}\in {\mathcal {F}}$ be a local minimizer of (P) and consider $\gamma \ge 1$. Assuming that $A^\gamma (u)$ holds in every unit direction *u* implies that options (b) or (c) from Theorem 4.1 yield M-stationarity of ${\bar{x}}$, and that option (d) cannot occur. Thus, we end up with ${\bar{x}}$ being M-stationary. Similarly, if $A^\gamma (u)$ holds in a critical direction $u\in {\mathbb {S}}_{{\mathbb {X}}}$ of order $(1,\gamma )$ for (P) at ${\bar{x}}$, (4.14) is satisfied. Due to (4.15), this also shows M-stationarity of ${\bar{x}}$. Thus, we obtain the following from Corollary 4.2.

#### Corollary 4.4

Let ${\bar{x}}\in {\mathcal {F}}$ be a local minimizer of (P) and consider $\gamma \ge 1$. Then each of the following conditions implies that ${\bar{x}}$ is M-stationary.

- Condition $A^\gamma (u)$ holds in each unit direction *u*.
- There are no critical directions for (P) at ${\bar{x}}$, or there is a critical direction $u\in {\mathbb {S}}_{{\mathbb {X}}}$ of order $(1,\gamma )$ for (P) at ${\bar{x}}$ such that $A^\gamma (u)$ holds.

In the following remark, we compare our approach from Corollary 4.4 with the results from Corollary 4.3 in the presence of any sufficient condition for directional metric subregularity.

#### Remark 4.3

Due to Corollary 4.3, directional metric subregularity serves as a constraint qualification guaranteeing M-stationarity of local minimizers. However, given $u\in {\mathbb {S}}_{{\mathbb {X}}}$, metric subregularity in direction *u* is difficult to verify, so it is often replaced by some stronger condition which is easier to check - exemplary, FOSCMS(*u*). Let us label such a sufficient condition as SCMS(*u*). Clearly, Corollary 4.3 can be restated in terms of SCMS(*u*). Suppose that we can show that $A^\gamma (u)$ is milder than SCMS(*u*) for every $u\in {\mathbb {S}}_{{\mathbb {X}}}$ (even strictly milder for some *u*). Naturally, option (a) from Corollary 4.4 then provides a (strictly) milder assumption than requiring SCMS(*u*) to hold for all unit directions. However, does an analogous relationship hold for the more complicated option (b) from Corollary 4.4? Both approaches yield M-stationarity if there are no critical directions. If there is a critical direction $u\in {\mathbb {S}}_{{\mathbb {X}}}$ such that SCMS(*u*) holds, then Lemma 4.2 yields that *u* is actually critical of order $(1,\gamma )$ and, thus, the milder assumption $A^\gamma (u)$ from the case (b) of Corollary 4.4 can be applied. This means that our approach via Corollary 4.4 is indeed better than an approach via any sufficient condition for metric subregularity in direction *u* which is stronger that $A^\gamma (u)$.

The second approach to M-stationarity can be called “asymptotic” and is based on the following result, a generalization of [63, Theorem 3.9], which reinspects Theorem 4.1 in the situation $\gamma :=1$. Particularly, we exploit that, in this case, both notions of a pseudo-coderivative from Definition 2.3 coincide with the directional limiting coderivative.

#### Corollary 4.5

Let ${\bar{x}}\in {\mathcal {F}}$ be a local minimizer of (P). Then ${\bar{x}}$ is M-stationary or there exist a critical direction $u\in {\mathbb {S}}_{{\mathbb {X}}}$ for (P) at ${\bar{x}}$, some $y^* \in {\mathbb {Y}}$, and sequences $\{x_k\}_{k\in {\mathbb {N}}},\{\eta _k\}_{k\in {\mathbb {N}}}\subset {\mathbb {X}}$ as well as $\{y_k\}_{k\in {\mathbb {N}}}\subset {\mathbb {Y}}$ such that $x_k\notin \varPhi ^{-1}({\bar{y}})$ and $y_k\ne {\bar{y}}$ for all $k\in {\mathbb {N}}$, satisfying the convergence properties

4.17a $$\begin{aligned} x_k&\rightarrow {\bar{x}},&\qquad y_k&\rightarrow {\bar{y}},&\qquad \eta _k&\rightarrow 0,&\end{aligned}$$

4.17b $$\begin{aligned} \frac{x_k-{\bar{x}}}{\Vert x_k-{\bar{x}}\Vert }&\rightarrow u,&\qquad \frac{y_k-{\bar{y}}}{\Vert x_k-{\bar{x}}\Vert }&\rightarrow 0,&\qquad{} & {} \end{aligned}$$

4.17c $$\begin{aligned} k\frac{\Vert y_k-{\bar{y}}\Vert }{\Vert x_k-{\bar{x}}\Vert }(y_k-{\bar{y}})&\rightarrow y^*,&\qquad k\Vert y_k-{\bar{y}}\Vert&\rightarrow \infty ,&\qquad{} & {} \end{aligned}$$

and

4.18 $$\begin{aligned} \forall k\in {\mathbb {N}}:\quad \eta _k\in \partial \varphi (x_k)+D^*\varPhi (x_k,y_k)\left( k(y_k-{\bar{y}})\right) . \end{aligned}$$

The above result shows that each local minimizer of (P) either is M-stationary or satisfies asymptotic stationarity conditions w.r.t. a certain critical direction and an unbounded sequence of multiplier estimates $\{\lambda _k\}_{k\in {\mathbb {N}}}$ given by

4.19 $$\begin{aligned} \forall k\in {\mathbb {N}}:\quad \lambda _k:=k(y_k-{\bar{y}}). \end{aligned}$$

Note that in the case where $\{\lambda _k\}_{k\in {\mathbb {N}}}$ would be bounded, one could simply take the limit in (4.18) along a suitable subsequence and, respecting the convergences from (4.17a), would end up with M-stationarity again taking into account robustness of the limiting subdifferential and coderivative. Thus, divergence of the multiplier estimates is natural since not all local minimizers of (P) are M-stationary in general, see [63, Lemma 3.4] as well. Related results in nondirectional form can be found in [58, 63]. The story of asymptotic stationarity conditions in variational analysis, however, can be traced back to [57, 59]. This concept has been rediscovered as a valuable tool for the analysis of convergence properties for solution algorithms associated with standard nonlinear optimization problems in [3, 7], and extensions were made to disjunctive, conic, and even infinite-dimensional optimization, see e.g. [2, 4, 26, 70] and the references therein.

The sequential information from (4.17) describes in great detail what must “go wrong” if M-stationarity fails. We will refer to (4.17a), (4.17b), and (4.17c) as basic, directional, and multiplier (sequential) information, respectively. Clearly, one can secure M-stationarity of a local minimizer by ruling out the second alternative in Corollary 4.5 and, as we will show, various known constraint qualifications for M-stationarity indeed do precisely that. Let us mention here two such conditions. Rescaling (4.18) by $\left\| \lambda _k\right\|$, for $\{\lambda _k\}_{k\in {\mathbb {N}}}$ as given in (4.19), and taking the limit $k\rightarrow \infty$ leads to a contradiction with the Mordukhovich criterion (2.10a), i.e., metric regularity of $\varPhi$ at $({\bar{x}},{\bar{y}})$. Respecting also the directional information (4.17b) yields a contradiction with FOSCMS(*u*) at $({\bar{x}},{\bar{y}})$.

In both cases, we have essentially discarded the multiplier information (4.17c) which deserves some remarks. We have used $\left\| \lambda _k\right\| \rightarrow \infty$, but this information is not really very important since, as we already explained, if the multipliers remain bounded, we end up with M-stationarity anyway. The fact that $\{\lambda _k \left\| y_k-{\bar{y}}\right\| /\left\| x_k-{\bar{x}}\right\| \}_{k\in {\mathbb {N}}}$ converges tells us how fast the multipliers $\{\lambda _k\}_{k\in {\mathbb {N}}}$ blow up. We note that the concept of super-coderivatives from Definition 2.4 collects this information, and we will come back to it in Sect. 5.3, where it is used to design constraint qualifications for M-stationarity. As we will show in Sect. 5.3, this approach is closely related to the hypothesis $A^\gamma (u)$ which we formulated in Assumption 4.1, and its role as a constraint qualification has already been illustrated in Corollary 4.4.

Finally, note that $(y_k-{\bar{y}})/\Vert y_k-{\bar{y}}\Vert = \lambda _k/\Vert \lambda _k\Vert$ means that the multipliers precisely capture the direction from which $\{y_k\}_{k\in {\mathbb {N}}}$ converges to ${\bar{y}}$. Particularly, we find $\langle \lambda _k/\Vert \lambda _k\Vert , (y_k-{\bar{y}})/\Vert y_k-{\bar{y}}\Vert \rangle = 1$, which is clearly more restrictive than the condition $\langle \lambda _k/\Vert \lambda _k\Vert , (y_k-{\bar{y}})/\Vert y_k-{\bar{y}}\Vert \rangle \rightarrow 1$. The latter convergence, which is used in the sufficient condition for metric subregularity in [37, Corollary 1], can be recast as $(y_k-{\bar{y}})/\Vert y_k-{\bar{y}}\Vert - \lambda _k/\Vert \lambda _k\Vert \rightarrow 0$. This information is respected by the new constraint qualifications which we are going to suggest in Sect. 5.

### Mixed-order necessary optimality conditions for optimization problems with geometric constraints in the case $\gamma :=2$

In this part, we apply Corollary 4.2 with $\gamma := 2$ to the case where $\varPhi :{\mathbb {X}}\rightrightarrows {\mathbb {Y}}$ is given in the form of a constraint mapping, i.e., $\varPhi (x):= g(x) - D$, $x\in {\mathbb {X}}$, holds where $g:{\mathbb {X}}\rightarrow {\mathbb {Y}}$ is twice continuously differentiable and $D \subset {\mathbb {Y}}$ is a closed set. Since, in Sect. 3, we computed the pseudo-coderivative and the graphical pseudo-derivative of order 2 of $\varPhi$, we are able to derive explicit conditions in terms of initial problem data. For that purpose, we assume ${\bar{y}}:=0$ in (P) throughout the section which can be done without loss of generality.

We start with a description of critical directions of order (1, 2) and (2, 2).

#### Lemma 4.3

Fix ${\bar{x}}\in {\mathcal {F}}$ and let $u \in {\mathbb {S}}_{{\mathbb {X}}}$ be a critical direction of order (1, 2) of (P) at ${\bar{x}}$. Suppose that ${\mathbb {Y}}:={\mathbb {R}}^m$ and *D* is locally polyhedral around $g({\bar{x}})$. Then

$$\begin{aligned} u \in {\mathcal {C}}^{1,2}({\bar{x}}):= \{ u \in {\mathbb {X}} \,|\, \textrm{d}\varphi ({\bar{x}})(u) \le 0,\, \exists s \in {\mathbb {X}}:\, w_s(u,0) \in {\textbf{T}}(u) \}, \end{aligned}$$

where $w_s(u,0)$ and ${\textbf{T}}(u)$ are defined in (3.7). If $\varphi$ is continuously differentiable, ${\mathcal {C}}^{1,2}({\bar{x}})$ corresponds precisely to the set of critical directions of order (1, 2) of (P) at ${\bar{x}}$. Moreover, if $\varphi$ is even twice continuously differentiable at ${\bar{x}}$, the set of all critical directions of order (2, 2) of (P) at ${\bar{x}}$ equals

4.20 $$\begin{aligned} \{ u \in {\mathbb {X}} \,|\, \exists s \in {\mathbb {X}}:\, \nabla \varphi ({\bar{x}}) s + 1/2 \nabla ^2 \varphi ({\bar{x}})[u,u] \in {\mathcal {T}}_{{\mathbb {R}}_-}(\nabla \varphi ({\bar{x}}) u), w_s(u,0) \in {\textbf{T}}(u) \}.\nonumber \\ \end{aligned}$$

#### Proof

A critical direction *u* of order (1, 2) of (P) at ${\bar{x}}$ satisfies $\textrm{d}\varphi ({\bar{x}})(u) \le 0$ and $0 \in D_2 \varPhi ({\bar{x}},0)(u)$, with equivalence being valid if $\varphi$ is continuously differentiable at ${\bar{x}}$. Hence, the first statement follows from Theorem 3.2.

By Proposition 4.1, a direction *u* is critical of order (2, 2) of (P) at ${\bar{x}}$ if and only if $u \in \ker D_2 M({\bar{x}},(\varphi ({\bar{x}}),0))$ for $M:{\mathbb {X}}\rightrightarrows {\mathbb {R}}\times {\mathbb {R}}^m$ given by $M(x):=(\varphi (x),g(x)) - ({\mathbb {R}}_- \times D)$, $x\in {\mathbb {X}}$. Hence, Theorem 3.2 can be applied again, yielding the second statement. $\square$

#### Remark 4.4

Note that Lemma 4.3 shows that the set of directions $C_2({\bar{x}})$ from [13, Theorem 3] and its extension labeled *second-order tightened critical cone* in [9, Theorem 3] actually correspond to ${\mathcal {C}}^{1,2}({\bar{x}})$, while the set of directions used in [37, Theorem 3(2)] corresponds to the one in (4.20). We believe that interpreting these directions as critical (of some order) is very natural. Moreover, our approach justifies the name. Indeed, as already mentioned, our definition of criticality is an extension of the one stated in [36, Definition 5]. More importantly, we have shown in Corollary 4.2 that in the absence of nonzero critical directions (of order $(1,\gamma )$ for some $\gamma \ge 1$), the corresponding mixed-order optimality conditions (involving a pseudo-coderivative of order $\gamma$) are satisfied without any additional assumptions.

Based on Theorems 3.1 and 3.2 as well as Corollary 4.2, we obtain the following result.

#### Proposition 4.2

Let ${\bar{x}}\in {\mathcal {F}}$ be a local minimizer of (P).

- If (3.9), as well as (3.10) or, in the case $\nabla g({\bar{x}})u\ne 0$, (3.11) hold for every unit direction, then ${\bar{x}}$ is M-stationary or there exist a critical direction $u \in {\mathbb {S}}_{{\mathbb {X}}}$ and
  4.21 $$\begin{aligned} y^* \in {\mathcal {N}}_{D}(g({\bar{x}});\nabla g({\bar{x}})u) \cap \ker \nabla g({\bar{x}})^*, \qquad z^* \in D{\mathcal {N}}_{D}(g({\bar{x}}),y^*)(\nabla g({\bar{x}})u) \nonumber \\ \end{aligned}$$
  such that
  4.22 $$\begin{aligned} 0 \in \partial \varphi ({\bar{x}};u) + \nabla ^2\langle y^*, g\rangle ({\bar{x}})(u) + \nabla g({\bar{x}})^* z^*. \end{aligned}$$
  If there exists a critical direction $u \in {\mathbb {S}}_{{\mathbb {X}}}$ of order (1, 2) of (P) at ${\bar{x}}$ satisfying (3.9), as well as (3.10) or, in the case $\nabla g({\bar{x}})u\ne 0$, (3.11), then there exist $y^*,z^*\in {\mathbb {Y}}$ satisfying (4.21) and (4.22) for this *u*.
- Let ${\mathbb {Y}}:={\mathbb {R}}^m$ and *D* be locally polyhedral around $g({\bar{x}})$. If either ${\mathcal {C}}^{1,2}({\bar{x}}) = \{0\}$ or if (3.13) holds for every unit direction, then ${\bar{x}}$ is M-stationary or there exist a critical direction $u \in {\mathbb {S}}_{{\mathbb {X}}}$, $s\in {\mathbb {X}}$, $y^*, z_i^* \in {\mathbb {R}}^m$ for $i=1,2$, and $\alpha \ge 0$, satisfying $\nabla g({\bar{x}})^* y^* =0$,
  $$\begin{aligned} y^*, z_1^* \in {\mathcal {N}}_{{\textbf{T}}(u)}(w_s(u,v)), \qquad z_2^* \in {\mathcal {T}}_{{\mathcal {N}}_{{\textbf{T}}(u)}(w_s(u,v))}(y^*), \end{aligned}$$
  and (4.22) (with $z^* = z_i^*, i=1,2$), where $v:= \alpha y^*$, and $w_s(u,v)$ and ${\textbf{T}}(u)$ have been defined in (3.7). If there exists $u \in {\mathcal {C}}^{1,2}({\bar{x}}) \cap {\mathbb {S}}_{{\mathbb {X}}}$ satisfying (3.13), then there exist $s\in {\mathbb {X}}$ and
  $$\begin{aligned} y^*, z_1^* \in {\mathcal {N}}_{{\textbf{T}}(u)}(w_s(u,0)), \qquad z_2^* \in {\mathcal {T}}_{{\mathcal {N}}_{{\textbf{T}}(u)}(w_s(u,0))}(y^*) \end{aligned}$$
  satisfying (4.22) (with $z^* = z_i^*, i=1,2$) as well as $\nabla g({\bar{x}})^* y^* =0$.

#### Proof

For the proof of (a), in the first alternative, we apply Corollary 3.1 in order to verify that (2.12) holds for every unit direction. Corollary 4.2 in turn yields that ${\bar{x}}$ is M-stationary or one of the cases (b) and (c) from Theorem 4.1 holds. In the case of Theorem 4.1 (b), however, from Theorem 3.1 (a) we get $0 \in \partial \varphi ({\bar{x}};u) + \nabla g({\bar{x}})^* z^*$ with

$$\begin{aligned} z^* \in D{\mathcal {N}}_{D}(g({\bar{x}}),0)(\nabla g({\bar{x}})u) \subset {\mathcal {N}}_D(g({\bar{x}});\nabla g({\bar{x}}) u) \subset {\mathcal {N}}_D(g({\bar{x}})), \end{aligned}$$

see Lemma 2.5, and M-stationarity of ${\bar{x}}$ follows. In the case of Theorem 4.1 (c), from (2.7) and Theorem 3.1 (a) we precisely obtain $y^*$ and $z^*$ as stated. Similarly, the second alternative follows from successively applying Corollaries 3.1 and  4.2, (2.7), and Theorem 3.1 (a).

For the proof of (b), we first would like to hint to Lemma 4.3. In the first alternative, taking into account Corollary 3.1, Corollary 4.2 yields that ${\bar{x}}$ is M-stationary or one of the cases (b) and (c) from Theorem 4.1 holds. As before, in the case of Theorem 4.1 (b), from Theorem 3.1 (b) we get $0 \in \partial \varphi ({\bar{x}};u) + \nabla g({\bar{x}})^* z^*$ with $z^* \in {\mathcal {N}}_{{\mathcal {T}}_D(g({\bar{x}}))}(\nabla g({\bar{x}}) u) \subset {\mathcal {N}}_D(g({\bar{x}}))$, see Lemma 2.2, and M-stationarity of ${\bar{x}}$ follows. In the case of Theorem 4.1 (c), from Theorem 3.2 we precisely obtain $y^*, z_1^*$, and $z_2^*$ as stated. The second alternative follows from Corollaries 3.1 and 4.2 as well as Theorem 3.2. $\square$

Similar optimality conditions involving a mixture of first- and second-order derivatives were proposed e.g. in [9, 11–13, 37]. Let us now explain that in the convex polyhedral case, where ${\mathbb {Y}}:={\mathbb {R}}^m$ holds while *D* is convex and polyhedral, all these optimality conditions are the same and can be stated simply as follows: If there exists $u \in {\mathcal {C}}^{1,2}({\bar{x}}) \cap {\mathbb {S}}_{{\mathbb {X}}}$ satisfying (3.17), then there are $y^*, z^* \in {\mathbb {R}}^m$ satisfying

4.23 $$\begin{aligned} \nabla \varphi ({\bar{x}}) + \nabla ^2\langle y^*,g\rangle ({\bar{x}})(u)+\nabla g({\bar{x}})^*z^*=0,\, \nabla g({\bar{x}})^* y^*=0,\, y^*, z^* \in {\mathcal {N}}_D(g({\bar{x}}))\nonumber \\ \end{aligned}$$

(for a fair comparison, we assume that $\varphi$ is continuously differentiable).

In Examples 3.1 and 3.2, we have shown that the 2-regularity assumption (3.17) used in [9] is, in general, strictly stronger than our condition (3.12), which is, in turn, strictly stronger than the mutually equivalent conditions (3.16) from [37] and (3.13) from Corollary 3.1. However, as shown in Corollary 3.2, all these assumptions are equivalent if applied to a critical direction *u* of order (1, 2), i.e, $u \in {\mathcal {C}}^{1,2}({\bar{x}})$, as this yields the existence of $s \in {\mathbb {X}}$ with $w_s(u,0) \in {\textbf{T}}(u)$.

Clearly, although the aforementioned qualification conditions are equivalent, the optimality conditions may differ due to the additional information regarding the multipliers. However, this is also not the case, and it can be shown following the proof of Proposition 3.1 (c). First, as mentioned above, we automatically have $s \in {\mathbb {X}}$ with $w_s(u,0) \in {\textbf{T}}(u)$ from $u \in {\mathcal {C}}^{1,2}({\bar{x}})$, which can be added to (4.23). Now, we are in the same situation as when proving Proposition 3.1 (c), but we have to work with (4.23) instead of (C$(u, y^*)$). From $u \in {\mathcal {C}}^{1,2}({\bar{x}})$ we also get $\nabla \varphi ({\bar{x}}) u \le 0$, while $w_s(u,0) \in {\textbf{T}}(u)$ and Lemma 3.2 yield $\nabla ^2 \langle y^*, g\rangle ({\bar{x}})[u,u]\le 0$, and $\langle z^*, \nabla g({\bar{x}}) u\rangle \le 0$ follows from $z^* \in {\mathcal {N}}_D(g({\bar{x}}))$ and $\nabla g({\bar{x}}) u \in {\mathcal {T}}_D(g({\bar{x}}))$, which is implicitly required due to $w_s(u,0)\in {\textbf{T}}(u)$. Thus, multiplying the essential equation of (4.23) by *u*, the three nonpositive terms sum up to zero, so they all must vanish. Hence, the arguments which we used to prove Proposition 3.1 (c) also work with (C$(u, y^*)$) replaced by (4.23).

### Applications

In this subsection, we highlight some aspects of our results from Sect. 4.3 in two popular settings of optimization theory. More precisely, we focus on the feasible regions of complementarity-constrained and nonlinear semidefinite problems. As mentioned at the end of Sect. 4.3, we do not obtain any new insights for standard nonlinear programs as these can be reformulated with the aid of a constraint mapping where the involved set is convex and polyhedral. Hence, we do not specify our findings for this elementary setting for brevity of presentation but refer the interested reader to [12, 13] where the associated mixed-order optimality conditions and constraint qualifications are worked out.

#### Mathematical programs with complementarity constraints

Let us introduce

$$\begin{aligned} {\mathcal {C}}:=({\mathbb {R}}_+\times \{0\})\cup (\{0\}\times {\mathbb {R}}_+), \end{aligned}$$

the so-called complementarity angle. For twice continuously differentiable functions $G,H:{\mathbb {X}}\rightarrow {\mathbb {R}}^m$ with components $G_1,\ldots ,G_m:{\mathbb {X}}\rightarrow {\mathbb {R}}$ and $H_1,\ldots ,H_m:{\mathbb {X}}\rightarrow {\mathbb {R}}$, we address the constraint region given by

MPCC $$\begin{aligned} (G_i(x),H_i(x))\in {\mathcal {C}}\quad i\in I \end{aligned}$$

where $I:=\{1,\ldots ,m\}$. The latter is distinctive for so called *mathematical programs with complementarity constraints* which have been studied intensively throughout the last decades, see e.g. [62, 68] for some classical references. We observe that (MPCC) can be formulated via a constraint map using $D:={\mathcal {C}}^m$. Note that standard inequality and equality constraints can be added without any difficulties due to Lemmas 2.3 and 2.7 when taking the findings from [12, 13] into account. Here, we omit them for brevity of presentation.

Fix some feasible point ${\bar{x}}\in {\mathbb {X}}$ of (MPCC). A critical direction $u\in {\mathbb {S}}_{{\mathbb {X}}}$ of the associated problem (P) necessarily needs to satisfy

4.24 $$\begin{aligned} \begin{aligned} \nabla G_i({\bar{x}}) u&=0\quad{} & {} i\in I^{0+}({\bar{x}}),&\\ \nabla H_i({\bar{x}}) u&=0\quad{} & {} i\in I^{+0}({\bar{x}}),&\\ (\nabla G_i({\bar{x}}) u,\nabla H_i({\bar{x}}) u)&\in {\mathcal {C}}{} & {} i\in I^{00}({\bar{x}}),&\end{aligned} \end{aligned}$$

where we used the well-known index sets

$$\begin{aligned} I^{0+}({\bar{x}}):= & {} \{i\in I\,|\,G_i({\bar{x}})=0,\,H_i({\bar{x}})>0\},\\ I^{+0}({\bar{x}}):= & {} \{i\in I\,|\,G_i({\bar{x}})>0,\,H_i({\bar{x}})=0\},\\ I^{00}({\bar{x}}):= & {} \{i\in I\,|\,G_i({\bar{x}})=0,\,H_i({\bar{x}})=0\}. \end{aligned}$$

We start with an illustration of Proposition 4.2 (a). Thanks to Remark 3.4, we need to check the constraint qualifications (3.9) and (3.14), and these can be specified to the present setting with the aid of Lemmas 2.2, 2.3 and 2.7. For brevity of presentation, we abstain from a discussion of the case where critical directions of order (1, 2) are involved. Based on the representation

$$\begin{aligned} {\text {gph}}{\mathcal {N}}_{{\mathcal {C}}} = ({\mathbb {R}}_+\times \{0\}\times \{0\}\times {\mathbb {R}})\cup (\{0\}\times {\mathbb {R}}_+\times {\mathbb {R}}\times \{0\}) \cup (\{0\}\times \{0\}\times {\mathbb {R}}_-\times {\mathbb {R}}_-), \end{aligned}$$

some elementary calculations show

4.25 $$\begin{aligned} D{\mathcal {N}}_{{\mathcal {C}}}((a,b),(\mu ,\nu ))(v) = {\left\{ \begin{array}{ll} \{0\}\times {\mathbb {R}}&{} a>0,\,b=\mu =0,\,v_2=0,\\ {\mathbb {R}}\times \{0\} &{} a=\nu =0,\,b>0,\,v_1=0,\\ {\mathbb {R}}^2 &{} a=b=0,\,\mu ,\nu<0,\,v=0,\\ \{0\}\times {\mathbb {R}}&{} a=b=\mu =0,\,\nu<0,\,v_1>0,\,v_2=0,\\ {\mathbb {R}}_-\times {\mathbb {R}}&{} a=b=\mu =0,\,\nu<0,\,v=0,\\ {\mathbb {R}}\times \{0\} &{} a=b=\nu =0,\,\mu<0,\,v_1=0,\,v_2>0,\\ {\mathbb {R}}\times {\mathbb {R}}_- &{} a=b=\nu =0,\,\mu <0,\,v=0,\\ \{0\}\times {\mathbb {R}}&{} a=b=\mu =0,\,\nu>0,\,v_1\ge 0,\,v_2=0,\\ {\mathbb {R}}\times \{0\} &{} a=b=\nu =0,\,\mu>0,\,v_1=0,\,v_2\ge 0,\\ \{0\}\times {\mathbb {R}}&{} a=b=\mu =\nu =0,\,v_1>0,\,v_2=0,\\ {\mathbb {R}}\times \{0\} &{} a=b=\mu =\nu =0,\,v_1=0,\,v_2>0,\\ {\mathcal {N}}_{{\mathcal {C}}}(0) &{} a=b=\mu =\nu =0,\,v=0,\\ \emptyset &{} \text {otherwise} \end{array}\right. }\nonumber \\ \end{aligned}$$

for arbitrary $((a,b),(\mu ,\nu ))\in {\text {gph}}{\mathcal {N}}_{{\mathcal {C}}}$ and $v\in {\mathbb {R}}^2$. Consequently, for $u\in {\mathbb {S}}_{{\mathbb {X}}}$ satisfying (4.24), (3.9) reduces to

4.26 $$\begin{aligned} \left. \begin{aligned}&\nabla G({\bar{x}})^*\mu +\nabla H({\bar{x}})^*\nu =0, \\&\sum \nolimits _{i=1}^m\bigl (\mu _i\nabla ^2G_i({\bar{x}})+\nu _i\nabla ^2H_i({\bar{x}})\bigr )u \\&\qquad +\nabla G({\bar{x}})^*{\tilde{\mu }}+\nabla H({\bar{x}})^*{\tilde{\nu }}=0, \\&\forall i\in I^{+0}({\bar{x}})\cup I^{00}_{+0}({\bar{x}},u):\,\mu _i=0, \\&\forall i\in I^{0+}({\bar{x}})\cup I^{00}_{0+}({\bar{x}},u):\,\nu _i=0, \\&\forall i\in I^{00}_{00}({\bar{x}},u):\,\mu _i,\nu _i\le 0\,\text { or }\,\mu _i\nu _i=0, \\&\forall i\in I:\,({\tilde{\mu }}_i,{\tilde{\nu }}_i) \in D{\mathcal {N}}_{{\mathcal {C}}} (({\bar{G}}_i,{\bar{H}}_i),(\mu _i,\nu _i))(\nabla {\bar{G}}_iu,\nabla {\bar{H}}_iu) \end{aligned} \right\} \quad \Longrightarrow \quad \mu =\nu =0,\nonumber \\ \end{aligned}$$

while (3.14) reads as

4.27 $$\begin{aligned} \left. \begin{aligned}&\nabla G({\bar{x}})^*\mu +\nabla H({\bar{x}})^*\nu =0,\, \nabla G({\bar{x}})^*{\tilde{\mu }}+\nabla H({\bar{x}})^*{\tilde{\nu }}=0,\\&\forall i\in I^{+0}({\bar{x}})\cup I^{00}_{+0}({\bar{x}},u):\,\mu _i=0,\\&\forall i\in I^{0+}({\bar{x}})\cup I^{00}_{0+}({\bar{x}},u):\,\nu _i=0,\\&\forall i\in I^{00}_{00}({\bar{x}},u):\,\mu _i,\nu _i\le 0\,\text { or }\,\mu _i\nu _i=0,\\&\forall i\in I:\,({\tilde{\mu }}_i,{\tilde{\nu }}_i) \in D{\mathcal {N}}_{{\mathcal {C}}} (({\bar{G}}_i,{\bar{H}}_i),(\mu _i,\nu _i))(\nabla {\bar{G}}_iu,\nabla {\bar{H}}_iu) \end{aligned} \right\} \quad \Longrightarrow \quad {\tilde{\mu }}={\tilde{\nu }}=0.\nonumber \\ \end{aligned}$$

Above, for each $i\in I$, we used ${\bar{G}}_i:=G_i({\bar{x}})$, ${\bar{H}}_i:=H_i({\bar{x}})$, $\nabla {\bar{G}}_iu:=\nabla G_i({\bar{x}}) u$, and $\nabla {\bar{H}}_iu:=\nabla H_i({\bar{x}}) u$ for brevity as well as the index sets

$$\begin{aligned} I^{00}_{0+}({\bar{x}},u)&:=\{i\in I^{00}({\bar{x}})\,|\,\nabla {\bar{G}}_iu=0,\,\nabla {\bar{H}}_iu>0\},\\ I^{00}_{+0}({\bar{x}},u)&:=\{i\in I^{00}({\bar{x}})\,|\,\nabla {\bar{G}}_iu>0,\,\nabla {\bar{H}}_iu=0\},\\ I^{00}_{00}({\bar{x}},u)&:=\{i\in I^{00}({\bar{x}})\,|\,\nabla {\bar{G}}_iu=0,\,\nabla {\bar{H}}_iu=0\}. \end{aligned}$$

The first assertion of Proposition 4.2 (a) now yields that whenever ${\bar{x}}$ is a local minimizer for the associated problem (P) and for each $u\in {\mathbb {S}}_{{\mathbb {X}}}$ satisfying (4.24), (4.26) and (4.27) hold, then ${\bar{x}}$ is either M-stationary, i.e., there are multipliers $\mu ,\nu \in {\mathbb {R}}^m$ satisfying

$$\begin{aligned}&0\in \partial \varphi ({\bar{x}})+\nabla G({\bar{x}})^*\mu +\nabla H({\bar{x}})^*\nu ,\\&\forall i\in I^{+0}({\bar{x}}):\,\mu _i=0,\\&\forall i\in I^{0+}({\bar{x}}):\,\nu _i=0,\\&\forall i\in I^{00}({\bar{x}}):\,\mu _i,\nu _i\le 0\,\text { or }\,\mu _i\nu _i=0, \end{aligned}$$

or we find $u\in {\mathbb {S}}_{{\mathbb {X}}}$ satisfying (4.24) and $\mathrm d\varphi ({\bar{x}})(u)\le 0$ as well as multipliers $\mu ,\nu ,{\tilde{\mu }},{\tilde{\nu }}\in {\mathbb {R}}^m$ such that

4.28 $$\begin{aligned} \begin{aligned}&0\in \partial \varphi ({\bar{x}};u) +\sum \limits _{i=1}^m\bigl (\mu _i\nabla ^2G_i({\bar{x}})+\nu _i\nabla ^2H_i({\bar{x}})\bigr )u +\nabla G({\bar{x}})^*{\tilde{\mu }}+\nabla H({\bar{x}})^*{\tilde{\nu }},\\&0=\nabla G({\bar{x}})^*\mu +\nabla H({\bar{x}})^*\nu ,\\&\forall i\in I^{+0}({\bar{x}})\cup I^{00}_{+0}({\bar{x}},u):\,\mu _i=0,\\&\forall i\in I^{0+}({\bar{x}})\cup I^{00}_{0+}({\bar{x}},u):\,\nu _i=0,\\&\forall i\in I^{00}_{00}({\bar{x}},u):\,\mu _i,\nu _i\le 0\,\text { or }\,\mu _i\nu _i=0,\\&\forall i\in I:\,({\tilde{\mu }}_i,{\tilde{\nu }}_i) \in D{\mathcal {N}}_{{\mathcal {C}}} (({\bar{G}}_i,{\bar{H}}_i),(\mu _i,\nu _i))(\nabla {\bar{G}}_iu,\nabla {\bar{H}}_iu). \end{aligned} \end{aligned}$$

For brevity, we present the results from Proposition 4.2 (b) only in simplified form, where $w_s(u,v)$ is replaced by 0, see Remark 3.3 as well, and we do not comment on the cases where critical directions of order (1, 2) are involved, but this would clearly yield further refinements.

In order to characterize (3.13), we observe that

$$\begin{aligned} {\mathcal {N}}_{{\mathcal {T}}_{{\mathcal {C}}}({\bar{G}}_i,{\bar{H}}_i)}(\nabla {\bar{G}}_iu,\nabla {\bar{H}}_iu) = {\left\{ \begin{array}{ll} \{0\}\times {\mathbb {R}}&{}i\in I^{+0}({\bar{x}})\cup I^{00}_{+0}({\bar{x}},u),\\ {\mathbb {R}}\times \{0\} &{}i\in I^{0+}({\bar{x}})\cup I^{00}_{0+}({\bar{x}},u),\\ {\mathcal {N}}_{{\mathcal {C}}}(0) &{}i\in I^{00}_{00}({\bar{x}},u) \end{array}\right. } \end{aligned}$$

is valid for each $i\in I$. For each pair $(\mu _i,\nu _i)\in {\mathcal {N}}_{{\mathcal {T}}_{{\mathcal {C}}}({\bar{G}}_i, {\bar{H}}_i)}(\nabla {\bar{G}}_iu,\nabla {\bar{H}}_iu)$, elementary calculations and a comparison with (4.25) show

$$\begin{aligned} {\mathcal {T}}_{{\mathcal {N}}_{{\mathcal {T}}_{{\mathcal {C}}}({\bar{G}}_i,{\bar{H}}_i)} (\nabla {\bar{G}}_iu,\nabla {\bar{H}}_iu)}(\mu _i,\nu _i)&= {\left\{ \begin{array}{ll} \{0\}\times {\mathbb {R}}&{}i\in I^{+0}({\bar{x}})\cup I^{00}_{+0}({\bar{x}},u),\\ {\mathbb {R}}\times \{0\} &{}i\in I^{0+}({\bar{x}})\cup I^{00}_{0+}({\bar{x}},u),\\ {\mathbb {R}}^2 &{}i\in I^{00}_{00}({\bar{x}},u),\,\mu _i<0,\,\nu _i<0,\\ {\mathbb {R}}_-\times {\mathbb {R}}&{}i\in I^{00}_{00}({\bar{x}},u),\,\mu _i=0,\,\nu _i<0,\\ {\mathbb {R}}\times {\mathbb {R}}_- &{}i\in I^{00}_{00}({\bar{x}},u),\,\mu _i<0,\,\nu _i=0,\\ \{0\}\times {\mathbb {R}}&{}i\in I^{00}_{00}({\bar{x}},u),\,\mu _i=0,\,\nu _i>0,\\ {\mathbb {R}}\times \{0\} &{}i\in I^{00}_{00}({\bar{x}},u),\,\mu _i>0,\,\nu _i=0,\\ {\mathcal {N}}_{{\mathcal {C}}}(0) &{}i\in I^{00}_{00}({\bar{x}},u),\,\mu _i=\nu _i=0 \end{array}\right. } \\&= D{\mathcal {N}}_{{\mathcal {C}}}(({\bar{G}}_i,{\bar{H}}_i),(\mu _i,\nu _i))(\nabla {\bar{G}}_i,\nabla {\bar{H}}_i). \end{aligned}$$

Thus, validity of (4.26) for each $u\in {\mathbb {S}}_{{\mathbb {X}}}$ satisfying (4.24) is already enough to infer that whenever ${\bar{x}}$ is a local minimizer, then it is either M-stationary or there are $u\in {\mathbb {S}}_{{\mathbb {X}}}$ satisfying (4.24) as well as $\mathrm d\varphi ({\bar{x}})(u)\le 0$ and multipliers $\mu ,\nu ,{\tilde{\mu }},{\tilde{\nu }}\in {\mathbb {R}}^m$ solving the stationarity conditions (4.28).

Let us further note that Proposition 4.2 (b) also allows for the consideration of a qualification and stationarity condition where simply $({\tilde{\mu }}_i,{\tilde{\nu }}_i)\in {\mathcal {N}}_{{\mathcal {T}}_{{\mathcal {C}}}({\bar{G}}_i,{\bar{H}}_i)}(\nabla {\bar{G}}_iu,\nabla {\bar{H}}_iu)$ has to hold for all $i\in I$, see Remark 3.3 again. One can easily check that there is no general inclusion between ${\mathcal {N}}_{{\mathcal {T}}_{{\mathcal {C}}}({\bar{G}}_i,{\bar{H}}_i)}(\nabla {\bar{G}}_iu,\nabla {\bar{H}}_iu)$ and ${\mathcal {T}}_{{\mathcal {N}}_{{\mathcal {T}}_{{\mathcal {C}}}({\bar{G}}_i,{\bar{H}}_i)}(\nabla {\bar{G}}_iu,\nabla {\bar{H}}_iu)}(\mu _i,\nu _i)$, i.e., this procedure leads to conditions not related to (4.26) and (4.28) which are, however, easier to evaluate.

The following example illustrates a situation where (4.26) is valid while (4.27) is violated, i.e., where Proposition 4.2 (b) is applicable while Proposition 4.2 (a) is not. This provides yet another justification of a separate consideration of the polyhedral situation.

##### Example 4.1

Let us consider (MPCC) with ${\mathbb {X}}:={\mathbb {R}}$, $m:=1$, and $G(x):=x$ as well as $H(x):=x^2$ for all $x\in {\mathbb {R}}$. We are interested in the unique feasible point ${\bar{x}}:=0$ of this system. The only direction from the unit sphere that satisfies (4.24) is $u:=1$. Hence, (4.26) reduces to

$$\begin{aligned} \left. \begin{aligned} \mu =0,\,2\nu +{\tilde{\mu }}=0,&\\ ({\tilde{\mu }},{\tilde{\nu }}) \in D{\mathcal {N}}_{{\mathcal {C}}} ((0,0),(\mu ,\nu ))(1,0)&\end{aligned} \right\} \quad \Longrightarrow \quad \mu =\nu =0. \end{aligned}$$

Let the premise be valid and assume $\nu \ne 0$. This gives $D{\mathcal {N}}_{{\mathcal {C}}}((0,0),(0,\nu ))(1,0)=\{0\}\times {\mathbb {R}}$ due to (4.25), i.e., ${\tilde{\mu }}=0$, and, thus, $\nu =0$ which yields a contradiction. Hence, this constraint qualification holds. However, (4.27) is given by

$$\begin{aligned} \left. \begin{aligned} \mu =0,\,{\tilde{\mu }}=0,&\\ ({\tilde{\mu }},{\tilde{\nu }}) \in D{\mathcal {N}}_{{\mathcal {C}}} ((0,0),(\mu ,\nu ))(1,0)&\end{aligned} \right\} \quad \Longrightarrow \quad {\tilde{\mu }}={\tilde{\nu }}=0, \end{aligned}$$

and one can easily check with the aid of (4.25) that the premise holds for $(\mu ,\nu ):=({\tilde{\mu }},{\tilde{\nu }}):=(0,1)$, i.e., this condition is violated.

Finally, we would like to refer the interested reader to [51, Section 6] and [50] where the theory of 2–regularity is first extended to mappings which are once but not twice differentiable and then applied to a suitable reformulation of complementarity constraints as a system of once but not twice differentiable equations. We abstain from a detailed comparison of our findings with the ones from [50, 51] for the following reasons. First, in these papers, a different way of stating the system of complementarity constraints is used, and it would be laborious to transfer the results to the formulation (MPCC). Second, at least in [51], some additional assumptions are used to simplify the calculations while we do not need to assume anything artificial to make the calculus accessible. Third, the final characterization of 2-regularity obtained in these papers does not comprise any second-order derivatives of the involved data functions and, thus, is anyhow clearly different from (4.26). Let us, however, close with the remark that the system of necessary optimality conditions derived in [50, Theorem 4.2] is closely related to (4.28).

#### Semidefinite programming

Let us consider the Hilbert space ${\mathcal {S}}_m$ of all real symmetric matrices equipped with the standard (Frobenius) inner product. We denote by ${\mathcal {S}}_m^+$ and ${\mathcal {S}}_m^-$ the cone of all positive and negative semidefinite matrices in ${\mathcal {S}}_m$, respectively. The foundations of variational analysis in this space can be found, e.g., in [25, Section 5.3]. For some twice continuously differentiable mapping $g:{\mathbb {X}}\rightarrow {\mathcal {S}}_m$, we investigate the constraint system

NLSD $$\begin{aligned} g(x)\in {\mathcal {S}}_m^+. \end{aligned}$$

It is well known that the closed, convex cone ${\mathcal {S}}_m^+$ is not polyhedral. Nevertheless, the constraint (NLSD), associated with so-called *nonlinear semidefinite programming*, can be encoded via a constraint map. Subsequently, we merely illustrate the first assertion of Proposition 4.2 (a). As ${\mathcal {S}}_m^+$ is not polyhedral, Lemma 4.3 cannot be used for a characterization of critical directions of order (1, 2).

Let ${\bar{x}}\in {\mathbb {X}}$ be feasible to (NLSD) and, for some $u\in {\mathbb {S}}_{{\mathbb {X}}}$, fix $\varOmega \in {\mathcal {N}}_{{\mathcal {S}}_m^+}(g({\bar{x}});\nabla g({\bar{x}})u)$. For later use, fix an orthogonal matrix ${\textbf{P}}\in {\mathbb {R}}^{m\times m}$ and a diagonal matrix $\mathbf \varLambda \in {\mathbb {R}}^{m\times m}$ whose diagonal elements $\lambda _1,\ldots ,\lambda _m$ are ordered nonincreasingly such that $g({\bar{x}})+\varOmega ={\textbf{P}}\mathbf \varLambda {\textbf{P}}^\top$. The index sets corresponding to the positive, zero, and negative entries on the main diagonal of $\mathbf \varLambda$ are denoted by $\alpha$, $\beta$, and $\gamma$, respectively. We emphasize that, here and throughout the subsection, $\alpha$ is a constant index set while $\beta$ and $\gamma$ depend on the precise choice of $\varOmega$. Subsequently, we use ${\textbf{Q}}^{{\textbf{P}}}:={\textbf{P}}^\top {\textbf{Q}}{\textbf{P}}$ and ${\textbf{Q}}^{{\textbf{P}}}_{IJ}:=({\textbf{Q}}^{{\textbf{P}}})_{IJ}$ for each matrix ${\textbf{Q}}\in {\mathcal {S}}_m$ and index sets $I,J\subset \{1,\ldots ,m\}$ where ${\textbf{M}}_{IJ}$ is the submatrix of ${\textbf{M}}\in {\mathcal {S}}_m$ which possesses only those rows and columns of ${\textbf{M}}$ whose indices can be found in *I* and *J*, respectively.

The above constructions yield

$$\begin{aligned} g({\bar{x}})={\textbf{P}}\max (\mathbf \varLambda ,{\textbf{O}}){\textbf{P}}^\top ,\qquad \varOmega ={\textbf{P}}\min (\mathbf \varLambda ,{\textbf{O}}){\textbf{P}}^\top \end{aligned}$$

where $\max$ and $\min$ have to be understood in entrywise fashion and ${\textbf{O}}$ is an all-zero matrix of appropriate dimensions. Due to

$$\begin{aligned} \nabla g({\bar{x}})u\in {\mathcal {T}}_{{\mathcal {S}}_m^+}(g({\bar{x}})) = \left\{ {\textbf{Q}}\in {\mathcal {S}}_m\,\bigg |\, {\textbf{Q}}^{{\textbf{P}}}_{\beta \cup \gamma ,\beta \cup \gamma }\in {\mathcal {S}}_{|\beta \cup \gamma |}^+ \right\} , \end{aligned}$$

we find

$$\begin{aligned} 0&= \langle \varOmega , \nabla g({\bar{x}})u\rangle = {\text {trace}}(\varOmega \,\nabla g({\bar{x}})u) = {\text {trace}}({\textbf{P}}\min (\mathbf \varLambda ,{\textbf{O}}){\textbf{P}}^\top {\textbf{P}}[\nabla g({\bar{x}})u]^{{\textbf{P}}}{\textbf{P}}^\top ) \\&= {\text {trace}}(\min (\mathbf \varLambda ,{\textbf{O}})[\nabla g({\bar{x}})u]^{{\textbf{P}}}) = \sum _{i\in \gamma }\underbrace{\lambda _i}_{<0}\,\underbrace{[\nabla g({\bar{x}})u]^{{\textbf{P}}}_{i,i}}_{\ge 0} \end{aligned}$$

which directly gives us $[\nabla g({\bar{x}})u]^{\textbf{P}}_{\beta \gamma }={\textbf{O}}$, $[\nabla g({\bar{x}})u]^{\textbf{P}}_{\gamma \gamma }={\textbf{O}}$, and $[\nabla g({\bar{x}})u]^{\textbf{P}}_{\beta \beta }\in {\mathcal {S}}_{|\beta |}^+$. Furthermore, we note

$$\begin{aligned} {\mathcal {N}}_{{\mathcal {S}}^+_m}(g({\bar{x}})) = \left\{ {\tilde{\varOmega }}\in {\mathcal {S}}_m\,\bigg |\, {\tilde{\varOmega }}^{\textbf{P}}_{\alpha \alpha }={\textbf{O}},\,{\tilde{\varOmega }}^{\textbf{P}}_{\alpha \beta } ={\textbf{O}},\,{\tilde{\varOmega }}^{{\textbf{P}}}_{\alpha \gamma }={\textbf{O}},\, {\tilde{\varOmega }}^{\textbf{P}}_{\beta \cup \gamma ,\beta \cup \gamma }\in {\mathcal {S}}^-_{|\beta \cup \gamma |} \right\} . \end{aligned}$$

Finally, let $\varXi _{\alpha \gamma }\in {\mathbb {R}}^{|\alpha |\times |\gamma |}$ be the matrix given by

$$\begin{aligned} \forall i\in \alpha \,\forall j\in \gamma :\quad [\varXi _{\alpha \gamma }]_{ij}:=-\frac{\lambda _j}{\lambda _i}. \end{aligned}$$

It is well known that the projection onto ${\mathcal {S}}_m^+$ is directionally differentiable. With the aid of Lemma 2.4 and [75, Corollary 3.1], we find

$$\begin{aligned}&D{\mathcal {N}}_{{\mathcal {S}}_m^+}(g({\bar{x}}),\varOmega )(\nabla g({\bar{x}})u)\\&\quad = \left\{ {\tilde{\varOmega }}\in {\mathcal {S}}_m\,\left| \, \begin{aligned}&{\tilde{\varOmega }}^{\textbf{P}}_{\alpha \alpha }={\textbf{O}},\,{\tilde{\varOmega }}^{\textbf{P}}_{\alpha \beta }={\textbf{O}},\, {\tilde{\varOmega }}^{\textbf{P}}_{\alpha \gamma }=\varXi _{\alpha \gamma } \bullet [\nabla g({\bar{x}})u]^{\textbf{P}}_{\alpha \gamma },\\&{\tilde{\varOmega }}^{\textbf{P}}_{\beta \beta }\in {\mathcal {S}}_{|\beta |}^-,\, \langle {\tilde{\varOmega }}^{\textbf{P}}_{\beta \beta }, [\nabla g({\bar{x}})u]^{\textbf{P}}_{\beta \beta }\rangle =0 \end{aligned}\right. \right\} , \end{aligned}$$

and if $\nabla g({\bar{x}})u\ne {\textbf{O}}$, we obtain

$$\begin{aligned}&D_sub {\mathcal {N}}_{{\mathcal {S}}_m^+}(g({\bar{x}}),\varOmega ) \left( \frac{\nabla g({\bar{x}})u}{\left\| \nabla g({\bar{x}})u\right\| }\right) \\&\qquad \subset \left\{ {\tilde{\varOmega }}\in {\mathcal {S}}_m\,\bigg |\, \begin{aligned}&{\tilde{\varOmega }}^{\textbf{P}}_{\alpha \alpha }={\textbf{O}},\,{\tilde{\varOmega }}^{\textbf{P}}_{\alpha \beta }={\textbf{O}},\, {\tilde{\varOmega }}^{\textbf{P}}_{\alpha \gamma }={\textbf{O}},\\&{\tilde{\varOmega }}^{\textbf{P}}_{\beta \beta }\in {\mathcal {S}}_{|\beta |}^-,\, \langle {\tilde{\varOmega }}^{\textbf{P}}_{\beta \beta }, [\nabla g({\bar{x}})u]^{\textbf{P}}_{\beta \beta }\rangle =0 \end{aligned} \right\} . \end{aligned}$$

Above, $\bullet$ represents the *Hadamard*, i.e., entrywise product. Note that validity of the final orthogonality condition in the estimate for the graphical subderivative follows from Lemma 2.4 since ${\tilde{\varOmega }}\in D_sub {\mathcal {N}}_{{\mathcal {S}}_m^+} (g({\bar{x}}),\varOmega )(\nabla g({\bar{x}})u/\left\| \nabla g({\bar{x}})u\right\| )$ and $\left\| \nabla g({\bar{x}})u\right\| >0$ yield

$$\begin{aligned} 0&\le \langle {\tilde{\varOmega }}, \nabla g({\bar{x}})u\rangle = {\text {trace}}\bigl ({\tilde{\varOmega }}\,\nabla g({\bar{x}})u\bigr ) = {\text {trace}}\bigl ({\textbf{P}}{\tilde{\varOmega }}^{{\textbf{P}}}{\textbf{P}}^\top {\textbf{P}}[\nabla g({\bar{x}})u]^{\textbf{P}}{\textbf{P}}^\top \bigr ) \\&= {\text {trace}}\bigl ({\tilde{\varOmega }}^{\textbf{P}}[\nabla g({\bar{x}})u]^{\textbf{P}}\bigr ) = {\text {trace}}\bigl ({\tilde{\varOmega }}^{\textbf{P}}_{\beta \beta }[\nabla g({\bar{x}})u]^{\textbf{P}}_{\beta \beta }\bigr ) \le 0 \end{aligned}$$

due to ${\tilde{\varOmega }}^{\textbf{P}}_{\alpha \alpha }={\textbf{O}}$, ${\tilde{\varOmega }}^{\textbf{P}}_{\alpha \beta }={\textbf{O}}$, ${\tilde{\varOmega }}^{\textbf{P}}_{\alpha \gamma }={\textbf{O}}$, ${\tilde{\varOmega }}^{\textbf{P}}_{\beta \beta }\in {\mathcal {S}}_{|\beta |}^-$, $[\nabla g({\bar{x}})u]^{\textbf{P}}_{\beta \gamma }={\textbf{O}}$, $[\nabla g({\bar{x}})u]^{\textbf{P}}_{\gamma \gamma }={\textbf{O}}$, and $[\nabla g({\bar{x}})u]^{\textbf{P}}_{\beta \beta }\in {\mathcal {S}}_{|\beta |}^+$. Thus, for each $u\in {\mathbb {S}}_{{\mathbb {X}}}$, (3.9) takes the form

$$\begin{aligned} \left. \begin{aligned}&\nabla g({\bar{x}})^*\varOmega =0,\, \nabla ^2\langle \varOmega , g\rangle ({\bar{x}})(u)+\nabla g({\bar{x}})^*{\tilde{\varOmega }}=0,\\&\varOmega ^{\textbf{P}}_{\alpha \alpha }={\textbf{O}},\,\varOmega ^{\textbf{P}}_{\alpha \beta }={\textbf{O}},\, \varOmega ^{{\textbf{P}}}_{\alpha \gamma }={\textbf{O}},\, \varOmega ^{\textbf{P}}_{\beta \cup \gamma ,\beta \cup \gamma }\in {\mathcal {S}}_{|\beta \cup \gamma |}^-,\\&[\nabla g({\bar{x}})u]^{\textbf{P}}_{\beta \gamma }={\textbf{O}},\, [\nabla g({\bar{x}})u]^{\textbf{P}}_{\gamma \gamma }={\textbf{O}},\, [\nabla g({\bar{x}})u]^{\textbf{P}}_{\beta \beta }\in {\mathcal {S}}_{|\beta |}^+,\\&{\tilde{\varOmega }}^{\textbf{P}}_{\alpha \alpha }={\textbf{O}},\,{\tilde{\varOmega }}^{\textbf{P}}_{\alpha \beta }={\textbf{O}},\, {\tilde{\varOmega }}^{\textbf{P}}_{\alpha \gamma } = \varXi _{\alpha \gamma }\bullet [\nabla g({\bar{x}})u]^{\textbf{P}}_{\alpha \gamma },\\&{\tilde{\varOmega }}^{\textbf{P}}_{\beta \beta }\in {\mathcal {S}}_{|\beta |}^-,\, \langle {\tilde{\varOmega }}^{\textbf{P}}_{\beta \beta }, [\nabla g({\bar{x}})u]^{\textbf{P}}_{\beta \beta }\rangle =0 \end{aligned} \right\} \quad \Longrightarrow \quad \varOmega ={\textbf{O}}, \end{aligned}$$

while (3.10) and (3.11) (the latter in the case $\nabla g({\bar{x}})u\ne {\textbf{O}}$) are both implied by

$$\begin{aligned} \left. \begin{aligned}&\nabla g({\bar{x}})^*\varOmega =0,\,\nabla g({\bar{x}})^*{\tilde{\varOmega }}=0,\\&\varOmega ^{\textbf{P}}_{\alpha \alpha }={\textbf{O}},\,\varOmega ^{\textbf{P}}_{\alpha \beta }={\textbf{O}},\, \varOmega ^{{\textbf{P}}}_{\alpha \gamma }={\textbf{O}},\, \varOmega ^{\textbf{P}}_{\beta \cup \gamma ,\beta \cup \gamma }\in {\mathcal {S}}_{|\beta \cup \gamma |}^-,\\&[\nabla g({\bar{x}})u]^{\textbf{P}}_{\beta \gamma }={\textbf{O}},\, [\nabla g({\bar{x}})u]^{\textbf{P}}_{\gamma \gamma }={\textbf{O}},\, [\nabla g({\bar{x}})u]^{\textbf{P}}_{\beta \beta }\in {\mathcal {S}}_{|\beta |}^+,\\&{\tilde{\varOmega }}^{\textbf{P}}_{\alpha \alpha }={\textbf{O}},\,{\tilde{\varOmega }}^{\textbf{P}}_{\alpha \beta }={\textbf{O}},\, {\tilde{\varOmega }}^{\textbf{P}}_{\alpha \gamma }={\textbf{O}},\\&{\tilde{\varOmega }}^{\textbf{P}}_{\beta \beta }\in {\mathcal {S}}_{|\beta |}^-,\, \langle {\tilde{\varOmega }}^{\textbf{P}}_{\beta \beta }, [\nabla g({\bar{x}})u]^{\textbf{P}}_{\beta \beta }\rangle =0 \end{aligned} \right\} \quad \Longrightarrow \quad {\tilde{\varOmega }}={\textbf{O}}. \end{aligned}$$

In the case where ${\bar{x}}$ is a local minimizer of the associated problem (P), validity of these conditions for each $u\in {\mathbb {S}}_{{\mathbb {X}}}$ guarantees that ${\bar{x}}$ is either M-stationary (we omit stating this well-known system here) or we find $u\in {\mathbb {S}}_{{\mathbb {X}}}$ and $\varOmega ,{\tilde{\varOmega }}\in {\mathcal {S}}_m$ such that

$$\begin{aligned}&0\in \partial \varphi ({\bar{x}};u)+\nabla ^2\langle \varOmega , g\rangle ({\bar{x}})(u) +\nabla g({\bar{x}})^*{\tilde{\varOmega }},\,\nabla g({\bar{x}})^*\varOmega =0,\\&\varOmega ^{\textbf{P}}_{\alpha \alpha }={\textbf{O}},\,\varOmega ^{\textbf{P}}_{\alpha \beta }={\textbf{O}},\, \varOmega ^{{\textbf{P}}}_{\alpha \gamma }={\textbf{O}},\, \varOmega ^{\textbf{P}}_{\beta \cup \gamma ,\beta \cup \gamma }\in {\mathcal {S}}_{|\beta \cup \gamma |}^-,\\&{\mathrm d\varphi ({\bar{x}})(u)\le 0},\, [\nabla g({\bar{x}})u]^{\textbf{P}}_{\beta \gamma }={\textbf{O}},\, [\nabla g({\bar{x}})u]^{\textbf{P}}_{\gamma \gamma }={\textbf{O}},\, [\nabla g({\bar{x}})u]^{\textbf{P}}_{\beta \beta }\in {\mathcal {S}}_{|\beta |}^+,\\&{\tilde{\varOmega }}^{\textbf{P}}_{\alpha \alpha }={\textbf{O}},\, {\tilde{\varOmega }}^{\textbf{P}}_{\alpha \beta }={\textbf{O}},\, {\tilde{\varOmega }}^{\textbf{P}}_{\alpha \gamma } =\varXi _{\alpha \gamma }\bullet [\nabla g({\bar{x}})u]^{\textbf{P}}_{\alpha \gamma },\\&{\tilde{\varOmega }}^{\textbf{P}}_{\beta \beta }\in {\mathcal {S}}_{|\beta |}^+,\, \langle {\tilde{\varOmega }}^{\textbf{P}}_{\beta \beta }, [\nabla g({\bar{x}})u]^{\textbf{P}}_{\beta \beta }\rangle =0. \end{aligned}$$

## Directional asymptotic regularity in nonsmooth optimization

In this section, we focus on (directional) asymptotic regularity conditions, which essentially correspond to conditions ensuring that (directional) asymptotic stationarity from Corollary 4.5, which serves as a necessary optimality condition for (P) even in the absence of constraint qualifications, translates into M-stationarity. We provide a comprehensive comparison of (directional) asymptotic regularity with various known constraint qualifications. Throughout the section, we consider a set-valued mapping $\varPhi :{\mathbb {X}}\rightrightarrows {\mathbb {Y}}$ with a closed graph.

### On the concept of directional asymptotic regularity

Based on Corollary 4.5, the following definition introduces concepts which may serve as (directional) qualification conditions for the model problem (P).

#### Definition 5.1

Let $({\bar{x}},{\bar{y}})\in {\text {gph}}\varPhi$ be fixed.

- The map $\varPhi$ is said to be *asymptotically regular at* $({\bar{x}},{\bar{y}})$ whenever the following condition holds: for every sequences $\{(x_k,y_k)\}_{k\in {\mathbb {N}}}\subset {\text {gph}}\varPhi$, $\{x_k^*\}_{k\in {\mathbb {N}}}\subset {\mathbb {X}}$, and $\{\lambda _k\}_{k\in {\mathbb {N}}}\subset {\mathbb {Y}}$ as well as $x^*\in {\mathbb {X}}$ satisfying $x_k\rightarrow {\bar{x}}$, $y_k\rightarrow {\bar{y}}$, $x_k^*\rightarrow x^*$, and $x_k^*\in {\widehat{D}}^*\varPhi (x_k,y_k)(\lambda _k)$ for all $k\in {\mathbb {N}}$, we find $x^*\in {\text {Im}}D^*\varPhi ({\bar{x}},{\bar{y}})$.
- For the fixed direction $u\in {\mathbb {S}}_{{\mathbb {X}}}$, $\varPhi$ is said to be *asymptotically regular at* $({\bar{x}},{\bar{y}})$ *in direction* *u* whenever the following condition holds: for every sequences $\{(x_k,y_k)\}_{k\in {\mathbb {N}}}\subset {\text {gph}}\varPhi$, $\{x_k^*\}_{k\in {\mathbb {N}}}\subset {\mathbb {X}}$, and $\{\lambda _k\}_{k\in {\mathbb {N}}}\subset {\mathbb {Y}}$ as well as $x^*\in {\mathbb {X}}$ and $y^*\in {\mathbb {Y}}$ satisfying $x_k\notin \varPhi ^{-1}({\bar{y}})$, $y_k\ne {\bar{y}}$, and $x_k^*\in {\widehat{D}}^*\varPhi (x_k,y_k)(\lambda _k)$ for each $k\in {\mathbb {N}}$ as well as the convergences
  5.1 $$\begin{aligned} \begin{aligned} x_k&\rightarrow {\bar{x}},&\qquad y_k&\rightarrow {\bar{y}},&\qquad x_k^*&\rightarrow x^*,&\\ \frac{x_k-{\bar{x}}}{\left\| x_k-{\bar{x}}\right\| }&\rightarrow u,&\qquad \frac{y_k-{\bar{y}}}{\left\| x_k-{\bar{x}}\right\| }&\rightarrow 0,&\qquad{} & {} \\ \frac{\left\| y_k-{\bar{y}}\right\| }{\left\| x_k-{\bar{x}}\right\| }\lambda _k&\rightarrow y^*,&\qquad \left\| \lambda _k\right\|&\rightarrow \infty ,&\qquad \frac{y_k-{\bar{y}}}{\left\| y_k-{\bar{y}}\right\| }-\frac{\lambda _k}{\left\| \lambda _k\right\| }&\rightarrow 0,&\end{aligned} \end{aligned}$$
  we find $$x^*\in {\text {Im}}D^*\varPhi ({\bar{x}},{\bar{y}})$$.
- For the fixed direction $u\in {\mathbb {S}}_{{\mathbb {X}}}$, $\varPhi$ is said to be *strongly asymptotically regular at* $({\bar{x}},{\bar{y}})$ *in direction* *u* whenever the following condition holds: for every sequences $\{(x_k,y_k)\}_{k\in {\mathbb {N}}}\subset {\text {gph}}\varPhi$, $\{x_k^*\}_{k\in {\mathbb {N}}}\subset {\mathbb {X}}$, and $\{\lambda _k\}_{k\in {\mathbb {N}}}\subset {\mathbb {Y}}$ as well as $x^*\in {\mathbb {X}}$ and $y^*\in {\mathbb {Y}}$ satisfying $x_k\notin \varPhi ^{-1}({\bar{y}})$, $y_k\ne {\bar{y}}$, and $x_k^*\in {\widehat{D}}^*\varPhi (x_k,y_k)(\lambda _k)$ for each $k\in {\mathbb {N}}$ as well as the convergences (5.1), we have $x^*\in {\text {Im}}D^*\varPhi (({\bar{x}},{\bar{y}});(u,0))$.

Before commenting in detail on these conditions, we would like to emphasize that they can be equivalently formulated in terms of limiting coderivatives completely. The mainly technical proof of this result can be found in Appendix A.

#### Proposition 5.1

Definition 5.1 can equivalently be formulated in terms of limiting normals.

Having Proposition 5.1 available, let us briefly note that asymptotic regularity of a set-valued mapping $\varPhi :{\mathbb {X}}\rightrightarrows {\mathbb {Y}}$ at some point $({\bar{x}},0)\in {\text {gph}}\varPhi$ in the sense of Definition 5.1 equals AM-regularity of the set $\varPhi ^{-1}(0)$ at ${\bar{x}}$ mentioned in [63, Remark 3.17]. The concepts of directional asymptotic regularity from Definition 5.1 (c) and (c) are new.

In the subsequent remark, we summarize some obvious relations between the different concepts from Definition 5.1.

#### Remark 5.1

Let $({\bar{x}},{\bar{y}})\in {\text {gph}}\varPhi$ be fixed. Then the following assertions hold.

- Let $u\in {\mathbb {S}}_{{\mathbb {X}}}$ be arbitrarily chosen. If $\varPhi$ is strongly asymptotically regular at $({\bar{x}},{\bar{y}})$ in direction *u*, it is asymptotically regular at $({\bar{x}},{\bar{y}})$ in direction *u*.
- If $\varPhi$ is asymptotically regular at $({\bar{x}},{\bar{y}})$, then it is asymptotically regular at $({\bar{x}},{\bar{y}})$ in each direction from ${\mathbb {S}}_{{\mathbb {X}}}$.

We note that strong asymptotic regularity in each unit direction is indeed not related to asymptotic regularity. On the one hand, the subsequently stated example, taken from [63, Example 3.15], shows that asymptotic regularity does not imply strong asymptotic regularity in each unit direction. On the other hand, Example 5.2 from below illustrates that strong asymptotic regularity in each unit direction does not yield asymptotic regularity.

#### Example 5.1

We consider $\varPhi :{\mathbb {R}}\rightrightarrows {\mathbb {R}}$ given by

$$\begin{aligned} \forall x\in {\mathbb {R}}:\quad \varPhi (x) {:}{=} {\left\{ \begin{array}{ll} {\mathbb {R}}&{}\text {if }x\le 0,\\ {[}x^2,\infty ) &{}\text {if }x>0 \end{array}\right. } \end{aligned}$$

at $({\bar{x}},{\bar{y}}):=(0,0)$. It is demonstrated in [63, Example 3.15] that $\varPhi$ is asymptotically regular at $({\bar{x}},{\bar{y}})$. We find ${\mathcal {T}}_{{\text {gph}}\varPhi }({\bar{x}},{\bar{y}})=\{(u,v)\in {\mathbb {R}}^2\,|\,u\le 0\,\text { or }\,v\ge 0\}$ so $(\pm 1,0)\in {\mathcal {T}}_{{\text {gph}}\varPhi }({\bar{x}},{\bar{y}})$. Let us consider $u:=1$. Then we find ${\text {Im}}D^*\varPhi (({\bar{x}},{\bar{y}});(u,0))=\{0\}$. Taking $x^*:=1$, $y^*:=1/2$, as well as

$$\begin{aligned} \forall k\in {\mathbb {N}}:\quad x_k:=\frac{1}{k}, \qquad y_k:=\frac{1}{k^2}, \qquad x_k^*:=1, \qquad \lambda _k:=\frac{k}{2}, \end{aligned}$$

we have $x_k^*\in {{\widehat{D}}}^*\varPhi (x_k,y_k)(\lambda _k)$ for all $k\in {\mathbb {N}}$ as well as the convergences (5.1). However, due to $x_k^*\rightarrow x^*\notin {\text {Im}}D^*\varPhi (({\bar{x}},{\bar{y}});(u,0))$, $\varPhi$ is not strongly asymptotically regular at $({\bar{x}},{\bar{y}})$ in direction *u*.

Combining Corollary 4.5 with the concepts from Definition 5.1, we immediately obtain the following result which motivates our interest in directional asymptotic regularity.

#### Corollary 5.1

Let ${\bar{x}}\in {\mathcal {F}}$ be a local minimizer of (P) such that, for each critical direction $u\in {\mathbb {S}}_{{\mathbb {X}}}$ for (P) at ${\bar{x}}$, $\varPhi$ is asymptotically regular at $({\bar{x}},{\bar{y}})$ in direction *u*. Then ${\bar{x}}$ is M-stationary.

#### Proof

Due to Corollary 4.5, it suffices to consider the situation where there are a critical direction $u\in {\mathbb {S}}_{{\mathbb {X}}}$ for (P) at ${\bar{x}}$ and $y^*\in {\mathbb {Y}}$ as well as sequences $\{x_k\}_{k\in {\mathbb {N}}},\{\eta _k\}_{k\in {\mathbb {N}}},\{x_k^*\}_{k\in {\mathbb {N}}}\subset {\mathbb {X}}$ and $\{y_k\}_{k\in {\mathbb {N}}}\subset {\mathbb {Y}}$ such that $x_k\notin \varPhi ^{-1}({\bar{y}})$, $y_k\ne {\bar{y}}$, $x_k^*\in \partial \varphi (x_k)$, and

$$\begin{aligned} \eta _k-x_k^*\in D^*\varPhi (x_k,y_k)\left( k(y_k-{\bar{y}})\right) \end{aligned}$$

for all $k\in {\mathbb {N}}$ as well as the convergences (4.17) are valid.

Since $\varphi$ is a locally Lipschitz continuous function, $\{x_k^*\}_{k\in {\mathbb {N}}}$ is bounded, see e.g. [66, Theorem 1.22], and, thus, converges (along a subsequence), to some point $x^*\in {\mathbb {X}}$ which belongs to $\partial \varphi ({\bar{x}})$ by robustness of the limiting subdifferential.

We can set $\lambda _k:=k(y_k-{\bar{y}})$ for each $k\in {\mathbb {N}}$ and obtain $\lambda _k\Vert y_k-{\bar{y}}\Vert /\Vert x_k-{\bar{x}}\Vert \rightarrow y^*$ and $\Vert \lambda _k\Vert \rightarrow \infty$ from (4.17c) as well as $(y_k-{\bar{y}})/\Vert y_k-{\bar{y}}\Vert =\lambda _k/\Vert \lambda _k\Vert$ for each $k\in {\mathbb {N}}$ by construction. Additionally, $\eta _k-x_k^*\in D^*\varPhi (x_k,y_k)(\lambda _k)$ is valid for each $k\in {\mathbb {N}}$.

Now, asymptotic regularity of $\varPhi$ at $({\bar{x}},{\bar{y}})$ in direction *u*, Proposition 5.1, and the remaining convergences from (4.17) yield $-x^*\in {\text {Im}}D^*\varPhi ({\bar{x}},{\bar{y}})$, i.e., there exists $\lambda \in {\mathbb {Y}}$ such that $-x^*\in D^*\varPhi ({\bar{x}},{\bar{y}})(\lambda )$. Recalling $x^*\in \partial \varphi ({\bar{x}})$ shows the claim. $\square$

In the light of Remark 5.1 (b), our result from Corollary 5.1 improves [63, Theorem 3.9] by a directional refinement of the constraint qualification since it suffices to check asymptotic regularity w.r.t. particular directions.

We point out that, unlike typical constraint qualifications, (directional) asymptotic regularity allows the existence of sequences satisfying (5.1) as long as the limit $x^*$ is included in ${\text {Im}}D^*\varPhi ({\bar{x}},{\bar{y}})$ which is enough for M-stationarity.

#### Remark 5.2

Corollary 5.1 requires asymptotic regularity in every (critical) unit direction. Taking into account Remark 4.2, we could also consider an alternative approach to secure M-stationarity, demanding either that there does not exist a critical direction together with the sequences from Definition 5.1 (c), or, in the case of existence, that $\varPhi$ is asymptotically regular at least in one of these critical directions. For brevity of presentation, we abstain from developing this approach further.

Since (directional) asymptotic regularity (w.r.t. all critical unit directions) yields M-stationarity of a local minimizer by Corollary 5.1, in the remaining part of the paper, we put it into context of other common assumptions that work as a constraint qualification for M-stationarity associated with problem (P). Let us clarify here some rather simple or known connections.

- A polyhedral mapping is asymptotically regular at each point of its graph.
- Metric regularity implies asymptotic regularity.
- Strong metric subregularity implies asymptotic regularity.
- FOSCMS does not imply asymptotic regularity, but it implies strong asymptotic regularity in each unit direction.
- Metric subregularity does not imply asymptotic regularity in each unit direction. However, if the map of interest is metrically subregular at every point of its graph near the reference point with a *uniform* constant, then strong asymptotic regularity in each unit direction follows.
- Neither asymptotic regularity nor strong directional asymptotic regularity yields the directional exact penalty property of Lemma 4.1.

Statements (a) and (b) were shown in [63, Theorems 3.10 and 3.12]. Let us now argue that strong metric subregularity (the “inverse” property associated with isolated calmness), see [31], also implies asymptotic regularity at the point. This follows easily from the discussion above [20, Corollary 4.6], which yields that the domain of the limiting coderivative, at the point where the mapping is isolatedly calm, is the whole space. Equivalently, the range of the limiting coderivative, at the point where the mapping is strongly metrically subregular, is the whole space and asymptotic regularity thus follows trivially. Thus, statement (c) follows.

Regarding (d), the fact that FOSCMS implies strong asymptotic regularity in each unit direction easily follows by similar arguments that show that metric regularity implies asymptotic regularity, see [63, Lemma 3.11, Theorem 3.12]. Actually, it can be proved that validity of FOSCMS(*u*) for some unit direction *u* implies strong asymptotic regularity in direction *u*. For constraint mappings, this also follows from Corollary 5.3 from below.

The following example shows that FOSCMS does not imply asymptotic regularity.

#### Example 5.2

Let $\varPhi :{\mathbb {R}}\rightrightarrows {\mathbb {R}}$ be given by

$$\begin{aligned} \forall x\in {\mathbb {R}}:\quad \varPhi (x):= {\left\{ \begin{array}{ll} [x,\infty ) &{} \text {if } x \le 0, \\ \left[ \frac{1}{k} - \frac{1}{k}\left( x - \frac{1}{k}\right) ,\infty \right) &{} \text {if } x \in \left( \frac{1}{k+1},\frac{1}{k}\right] \text { for some }k\in {\mathbb {N}},\\ \emptyset &{}\text {otherwise.} \end{array}\right. } \end{aligned}$$

Then $\{(1/k,1/k)\}_{k\in {\mathbb {N}}}\subset {\text {gph}}\varPhi$ converges to $({\bar{x}},{\bar{y}}):=(0,0)$ and

$$\begin{aligned} {\mathcal {N}}_{{\text {gph}}\varPhi }(1/k,1/k) = \{(x^*,y^*)\in {\mathbb {R}}^2 \,\vert \, y^* \le 0, y^* \le k x^*\} \end{aligned}$$

is valid showing that ${\text {Im}}D^*\varPhi (1/k,1/k) = {\mathbb {R}}$ holds for all $k\in {\mathbb {N}}$. On the other hand, we have

$$\begin{aligned} {\mathcal {N}}_{{\text {gph}}\varPhi }(0,0) = \{(x^*,y^*)\in {\mathbb {R}}^2 \,\vert \, x^* \ge 0, y^* \le 0\}, \end{aligned}$$

and, thus, ${\text {Im}}D^*\varPhi (0,0) = {\mathbb {R}}_+$. This means that $\varPhi$ is not asymptotically regular at $({\bar{x}},{\bar{y}})$.

On the other hand, we find

$$\begin{aligned} {\mathcal {T}}_{{\text {gph}}\varPhi }({\bar{x}},{\bar{y}})=\{(u,v)\in {\mathbb {R}}^2\,|\,u\le v\}. \end{aligned}$$

Each pair $(u,0)\in {\mathcal {T}}_{{\text {gph}}\varPhi }({\bar{x}},{\bar{y}})$ with $u\ne 0$ satisfies $u<0$, i.e., the direction (*u*, 0) points into the interior of ${\text {gph}}\varPhi$. Thus, we have ${\mathcal {N}}_{{\text {gph}}\varPhi }(({\bar{x}},{\bar{y}}),(u,0))=\{(0,0)\}$ which shows that FOSCMS is valid.

Regarding (e), let us fix $({\bar{x}},{\bar{y}})\in {\text {gph}}\varPhi$ and note that metric subregularity of $\varPhi$ on a neighborhood of $({\bar{x}},{\bar{y}})$ (restricted to ${\text {gph}}\varPhi$) with a uniform constant $\kappa >0$ is clearly milder than metric regularity at $({\bar{x}},{\bar{y}})$ since it is automatically satisfied, e.g., for polyhedral mappings. To see that it implies asymptotic regularity, consider sequences $\{(x_k,y_k)\}_{k\in {\mathbb {N}}}\subset {\text {gph}}\varPhi$, $\{x_k^*\}_{k\in {\mathbb {N}}}\subset {\mathbb {X}}$, and $\{\lambda _k\}_{k\in {\mathbb {N}}}\subset {\mathbb {Y}}$ as well as $x^*\in {\mathbb {X}}$ and $y^*\in {\mathbb {Y}}$ satisfying $x_k^*\in {\widehat{D}}^*\varPhi (x_k,y_k)(\lambda _k)$ for each $k\in {\mathbb {N}}$ and the convergences (5.1) for some unit direction $u\in {\mathbb {S}}_{{\mathbb {X}}}$. Due to [20, Theorem 3.2] and $-x_k^*\in {\text {dom}}{\widehat{D}}^*\varPhi ^{-1}(y_k,x_k)$, we find $x_k^*\in \widehat{{\mathcal {N}}}_{\varPhi ^{-1}(y_k)}(x_k) \subset {\mathcal {N}}_{\varPhi ^{-1}(y_k)}(x_k)$ for each $k\in {\mathbb {N}}$. Furthermore, [20, Theorem 3.2] also gives the existence of ${\tilde{\lambda }}_k\in {\mathbb {Y}}$ with $\Vert {\tilde{\lambda }}_k\Vert \le \kappa \Vert {x_k^*}\Vert$ and $x_k^*\in D^*\varPhi (x_k,y_k)({\tilde{\lambda }}_k)$. Noting that $\{x_k^*\}_{k\in {\mathbb {N}}}$ converges, this shows that there is an accumulation point $\lambda \in {\mathbb {Y}}$ of $\{{\tilde{\lambda }}_k\}_{k\in {\mathbb {N}}}$ which satisfies $x^*\in D^*\varPhi (({\bar{x}},{\bar{y}});(u,0))(\lambda )$ by robustness of the directional limiting coderivative, see Lemma 2.1. Hence, $\varPhi$ is strongly asymptotically regular at $({\bar{x}},{\bar{y}})$ in direction *u*. Note that for the above arguments to work, we only need uniform metric subregularity along all sequences $\{(x_k,y_k)\}_{k\in {\mathbb {N}}}\subset {\text {gph}}\varPhi$ converging to $({\bar{x}}, {\bar{y}})$ from direction (*u*, 0).

The following example shows that metric subregularity in the neighborhood of the point of interest does not imply asymptotic regularity in each unit direction.

#### Example 5.3

We consider the mapping $\varPhi :{\mathbb {R}}\rightrightarrows {\mathbb {R}}$ given by

$$\begin{aligned} \forall x\in {\mathbb {R}}:\quad \varPhi (x):=\{0,x^2\}. \end{aligned}$$

Due to $\varPhi ^{-1}(0)={\mathbb {R}}$, $\varPhi$ is metrically subregular at all points (*x*, 0) where $x\in {\mathbb {R}}$ is arbitrary. Furthermore, at all points $(x,x^2)$ where $x\ne 0$ holds, the Mordukhovich criterion (2.10a) shows that $\varPhi$ is metrically regular. Thus, $\varPhi$ is metrically subregular at each point of its graph. Note that the moduli of metric subregularity tend to $\infty$ along the points $(x,x^2)$ as $x\downarrow 0$ or $x\uparrow 0$.

Let us consider the point $({\bar{x}},{\bar{y}}):=(0,0)$ where we have ${\mathcal {N}}_{{\text {gph}}\varPhi }({\bar{x}},{\bar{y}})=\{0\}\times {\mathbb {R}}$ and, thus, ${\text {Im}}D^*\varPhi ({\bar{x}},{\bar{y}})=\{0\}$. Choosing $x^*:=1$, $y^*:=1/2$, as well as

$$\begin{aligned} \forall k\in {\mathbb {N}}:\quad x_k:=\frac{1}{k},\qquad y_k:=\frac{1}{k^2},\qquad x_k^*:=1,\qquad \lambda _k:=\frac{k}{2}, \end{aligned}$$

we have $x_k^*\in {{\widehat{D}}}^*\varPhi (x_k,y_k)(\lambda _k)$ for all $k\in {\mathbb {N}}$ as well as the convergences (5.1) for $u:=1$. Due to $x_k^*\rightarrow x^*\notin {\text {Im}}D^*\varPhi ({\bar{x}},{\bar{y}})$, $\varPhi$ is not asymptotically regular at $({\bar{x}},{\bar{y}})$ in direction *u*.

Finally, let us address item (f) with the aid of an example.

#### Example 5.4

Let us define $\varphi :{\mathbb {R}}\rightarrow {\mathbb {R}}$ and $\varPhi :{\mathbb {R}}\rightrightarrows {\mathbb {R}}$ by means of

$$\begin{aligned} \forall x\in {\mathbb {R}}:\quad \varphi (x):=-x, \qquad \varPhi (x):= {\left\{ \begin{array}{ll} {\mathbb {R}}&{}\text {if }x\le 0,\\ {[}x^2,\infty ) &{}\text {if }x=\frac{1}{k}\text { for some }k\in {\mathbb {N}},\\ \emptyset &{}\text {otherwise.} \end{array}\right. } \end{aligned}$$

Furthermore, we fix ${\bar{y}}:=0$. One can easily check that ${\bar{x}}:=0$ is the uniquely determined global minimizer of the associated problem (P). Furthermore, we have ${\text {Im}}D^*\varPhi ({\bar{x}},{\bar{y}})={\text {Im}}D^*\varPhi (({\bar{x}},{\bar{y}});(1,0))={\mathbb {R}}$ which shows that $\varPhi$ is asymptotically regular at $({\bar{x}},{\bar{y}})$ as well as strongly asymptotically regular at $({\bar{x}},{\bar{y}})$ in direction 1. Furthermore, it is obvious that $\varPhi$ is strongly asymptotically regular at $({\bar{x}},{\bar{y}})$ in direction $-1$. Finally, let us mention that $\varPhi$ fails to be metrically subregular at $({\bar{x}},{\bar{y}})$ in direction 1.

Now, define $x_k:=1/k$ for each $k\in {\mathbb {N}}$ and observe that for each constant $C>0$ and sufficiently large $k\in {\mathbb {N}}$, we have $\varphi (x_k)+C\,{\text {dist}}({\bar{y}},\varPhi (x_k))=-1/k+C/k^2<0=\varphi ({\bar{x}})$, i.e., ${\bar{x}}$ is not a minimizer of (4.1) for any choice of $C>0$, $\varepsilon >0$, $\delta >0$, and $u:=1$.

### Directional pseudo- and quasi-normality

In this section, we connect asymptotic regularity with the notions of pseudo- and quasi-normality. Note that the latter concepts have been introduced for standard nonlinear programs in [24, 46], and extensions to more general geometric constraints have been established in [43]. Furthermore, problem-tailored notions of these conditions have been coined e.g. for so-called cardinality-, complementarity-, and switching-constrained optimization problems, see [52, 54, 61]. Let us point out that these conditions are comparatively mild constraint qualifications and sufficient for the presence of metric subregularity of the associated constraint mapping, see e.g. [43, Theorem 5.2]. Here, we extend pseudo- and quasi-normality from the common setting of geometric constraint systems to arbitrary set-valued mappings and comment on the qualitative properties of these conditions. Naturally, we aim for directional versions of these concepts, which, in the setting of geometric constraints, were recently introduced in [15] and further explored in [16].

#### On the general concept of directional pseudo- and quasi-normality

The definition below introduces the notions of our interest.

##### Definition 5.2

Fix $({\bar{x}},{\bar{y}})\in {\text {gph}}\varPhi$ and a direction $u\in {\mathbb {S}}_{{\mathbb {X}}}$.

- We say that *pseudo-normality in direction* *u* holds at $({\bar{x}},{\bar{y}})$ if there does not exist a nonzero vector $\lambda \in \ker D^*\varPhi (({\bar{x}},{\bar{y}});(u,0))$ satisfying the following condition: there are sequences $\{(x_k,y_k)\}_{k\in {\mathbb {N}}}\subset {\text {gph}}\varPhi$ with $x_k\ne {\bar{x}}$ for all $k\in {\mathbb {N}}$ and $\{\lambda _k\}_{k\in {\mathbb {N}}}\subset {\mathbb {Y}}$, $\{\eta _k\}_{k\in {\mathbb {N}}}\subset {\mathbb {X}}$, such that
  5.2 $$\begin{aligned} \begin{aligned} x_k&\rightarrow {\bar{x}},&\qquad y_k&\rightarrow {\bar{y}},&\qquad \lambda _k&\rightarrow \lambda ,&\\ \eta _k&\rightarrow 0,&\qquad \frac{x_k-{\bar{x}}}{\left\| x_k-{\bar{x}}\right\| }&\rightarrow u,&\qquad \frac{y_k-{\bar{y}}}{\left\| x_k-{\bar{x}}\right\| }&\rightarrow 0,&\end{aligned} \end{aligned}$$
  and $\eta _k\in {\widehat{D}}^*\varPhi (x_k,y_k)(\lambda _k)$ as well as $\langle \lambda , y_k-{\bar{y}}\rangle >0$ for all $k\in {\mathbb {N}}$.
- Let ${\mathcal {E}}:=\{e_1,\ldots ,e_m\}\subset {\mathbb {Y}}$ be an orthonormal basis of ${\mathbb {Y}}$. We say that *quasi-normality in direction u* holds at $({\bar{x}},{\bar{y}})$ w.r.t. ${\mathcal {E}}$ if there does not exist a nonzero vector $\lambda \in \ker D^*\varPhi (({\bar{x}},{\bar{y}});(u,0))$ satisfying the following condition: there are sequences $\{(x_k,y_k)\}_{k\in {\mathbb {N}}}\subset {\text {gph}}\varPhi$ with $x_k\ne {\bar{x}}$ for all $k\in {\mathbb {N}}$ and $\{\lambda _k\}_{k\in {\mathbb {N}}}\subset {\mathbb {Y}}$, $\{\eta _k\}_{k\in {\mathbb {N}}}\subset {\mathbb {X}}$, such that we have the convergences from (5.2) and, for all $k\in {\mathbb {N}}$ and $i\in \{1,\ldots ,m\}$, $\eta _k\in {\widehat{D}}^*\varPhi (x_k,y_k)(\lambda _k)$ as well as $\langle \lambda , e_i\rangle \langle y_k-{\bar{y}}, e_i\rangle >0$ if $\langle \lambda , e_i\rangle \ne 0$.

In the case where the canonical basis is chosen in ${\mathbb {Y}}:={\mathbb {R}}^m$, the above concept of quasi-normality is a direct generalization of the original notion from [24] which was coined for standard nonlinear problems and neglected directional information. Let us just mention that a reasonable, basis-independent definition of quasi-normality would require that there exists some basis w.r.t. which the mapping of interest is quasi-normal, see also Theorem 5.1.

Note that the sequence $\{y_k\}_{k\in {\mathbb {N}}}$ in the definition of directional pseudo- and quasi-normality needs to satisfy $y_k\ne {\bar{y}}$ for all $k\in {\mathbb {N}}$. In the definition of directional pseudo-normality, this is clear from $\langle \lambda , y_k-{\bar{y}}\rangle >0$ for all $k\in {\mathbb {N}}$. Furthermore, in the definition of directional quasi-normality, observe that $\lambda \ne 0$ implies the existence of $j\in \{1,\ldots ,m\}$ such that $\langle \lambda , e_j\rangle \ne 0$ holds, so that $\langle y_k-{\bar{y}}, e_j\rangle \ne 0$ is necessary for each $k\in {\mathbb {N}}$.

In the following lemma, we show the precise relation between directional pseudo- and quasi-normality.

##### Lemma 5.1

Fix $({\bar{x}},{\bar{y}})\in {\text {gph}}\varPhi$ and some direction $u\in {\mathbb {S}}_{{\mathbb {X}}}$. Then $\varPhi$ is pseudo-normal at $({\bar{x}},{\bar{y}})$ in direction *u* if and only if $\varPhi$ is quasi-normal at $({\bar{x}},{\bar{y}})$ in direction *u* w.r.t. each orthonormal basis of ${\mathbb {Y}}$.

##### Proof

$[\Longrightarrow ]$ Let $\varPhi$ be pseudo-normal at $({\bar{x}},{\bar{y}})$ in direction *u*, let ${\mathcal {E}}:=\{e_1,\ldots ,e_m\}\subset {\mathbb {Y}}$ be an orthonormal basis of ${\mathbb {Y}}$, and pick $\lambda \in \ker D^*\varPhi (({\bar{x}},{\bar{y}});(u,0))$ as well as sequences $\{(x_k,y_k)\}_{k\in {\mathbb {N}}}\subset {\text {gph}}\varPhi$ with $x_k\ne {\bar{x}}$ for all $k\in {\mathbb {N}}$ and $\{\lambda _k\}_{k\in {\mathbb {N}}}\subset {\mathbb {Y}}$, $\{\eta _k\}_{k\in {\mathbb {N}}}\subset {\mathbb {X}}$, satisfying the convergences (5.2) and, for all $k\in {\mathbb {N}}$ and $i\in \{1,\ldots ,m\}$, $\eta _k\in {\widehat{D}}^*\varPhi (x_k,y_k)(\lambda _k)$ as well as $\langle \lambda , e_i\rangle \langle y_k-{\bar{y}}, e_i\rangle >0$ if $\langle \lambda , e_i\rangle \ne 0$. Observing that we have

$$\begin{aligned} \langle \lambda , y_k-{\bar{y}}\rangle&=\left\langle {\sum \limits _{i=1}^m\langle \lambda , e_i\rangle e_i}{\sum \limits _{j=1}^m\langle y_k-{\bar{y}}, e_j\rangle e_j}\right\rangle \\&= \sum \limits _{i=1}^m\sum \limits _{j=1}^m\langle \lambda , e_i\rangle \langle y_k-{\bar{y}}, e_j\rangle \langle e_i, e_j\rangle \\&= \sum \limits _{i=1}^m\langle \lambda , e_i\rangle \langle y_k-{\bar{y}}, e_i\rangle , \end{aligned}$$

validity of pseudo-normality at $({\bar{x}},{\bar{y}})$ in direction *u* gives $\lambda =0$, i.e., $\varPhi$ is quasi-normal at $({\bar{x}},{\bar{y}})$ in direction *u* w.r.t. ${\mathcal {E}}$.

$[\Longleftarrow ]$ Assume that $\varPhi$ is quasi-normal at $({\bar{x}},{\bar{y}})$ in direction *u* w.r.t. each orthonormal basis of ${\mathbb {Y}}$. Suppose that $\varPhi$ is not pseudo-normal at $({\bar{x}},{\bar{y}})$ in direction *u*. Then we find some nonzero $\lambda \in \ker D^*\varPhi (({\bar{x}},{\bar{y}});(u,0))$ as well as sequences $\{(x_k,y_k)\}_{k\in {\mathbb {N}}}\subset {\text {gph}}\varPhi$ with $x_k\ne {\bar{x}}$ for all $k\in {\mathbb {N}}$ and $\{\lambda _k\}_{k\in {\mathbb {N}}}\subset {\mathbb {Y}}$, $\{\eta _k\}_{k\in {\mathbb {N}}}\subset {\mathbb {X}}$, satisfying the convergences (5.2) and $\eta _k\in {\widehat{D}}^*\varPhi (x_k,y_k)(\lambda _k)$ as well as $\langle \lambda , y_k-{\bar{y}}\rangle >0$ for all $k\in {\mathbb {N}}$. Noting that $\lambda$ does not vanish, we can construct an orthonormal basis ${\mathcal {E}}_\lambda :=\{e_1^\lambda ,\ldots ,e_m^\lambda \}$ of ${\mathbb {Y}}$ with $e_1^\lambda :=\lambda /\left\| \lambda \right\|$. Note that, for $i\in \{1,\ldots ,m\}$, we have $\langle \lambda , e_i^\lambda \rangle \ne 0$ if and only if $i=1$ by construction of ${\mathcal {E}}_\lambda$. Furthermore, we find

$$\begin{aligned} \langle \lambda , e_1^\lambda \rangle \langle y_k-{\bar{y}}, e_1^\lambda \rangle = \left\| \lambda \right\| \langle \lambda /\left\| \lambda \right\| , y_k-{\bar{y}}\rangle = \langle \lambda , y_k-{\bar{y}}\rangle > 0. \end{aligned}$$

This, however, contradicts quasi-normality of $\varPhi$ at $({\bar{x}},{\bar{y}})$ in direction *u* w.r.t. ${\mathcal {E}}_{\lambda }$. $\square$

Let us note that [24, Example 1] shows in the nondirectional situation of standard nonlinear programming that pseudo-normality might be more restrictive than quasi-normality w.r.t. the canonical basis in ${\mathbb {R}}^m$. On the other hand, due to Lemma 5.1, there must exist another basis such that quasi-normality w.r.t. this basis fails since pseudo-normality fails. This depicts that validity of quasi-normality indeed may depend on the chosen basis. In [15], the authors define directional quasi-normality for geometric constraints in Euclidean spaces in componentwise fashion although this is somehow unclear in situations where the image space is different from ${\mathbb {R}}^m$. Exemplary, in the $\tfrac{1}{2}m(m+1)$-dimensional space ${\mathcal {S}}_m$ of all real symmetric $m\times m$-matrices, the canonical basis, which seems to be associated with a componentwise calculus, comprises precisely $\tfrac{1}{2}(m-1)m$ matrices with precisely two nonzero entries. Our definition of quasi-normality from Definition 5.2 gives some more freedom since the choice of the underlying basis allows to *rotate* the coordinate system.

Following the arguments in [16, Section 3.2], it also might be reasonable to define intermediate conditions bridging pseudo- and quasi-normality. In the light of this paper, however, the concepts from Definition 5.2 are sufficient for our purposes.

As the following theorem shows, directional quasi- and, thus, pseudo-normality also serve as sufficient conditions for strong directional asymptotic regularity and directional metric subregularity which explains our interest in these conditions. Both statements follow once we clarify that pseudo- and quasi-normality are in fact specifications of the multiplier sequential information in (5.1), namely the convergence $(y_k-{\bar{y}})/\Vert y_k-{\bar{y}}\Vert - \lambda _k/\Vert \lambda _k\Vert \rightarrow 0$.

##### Theorem 5.1

If $\varPhi :{\mathbb {X}} \rightrightarrows {\mathbb {Y}}$ is quasi-normal in direction $u \in {\mathbb {S}}_{{\mathbb {X}}}$ at $({\bar{x}}, {\bar{y}}) \in {\text {gph}}\varPhi$ w.r.t. some orthonormal basis ${\mathcal {E}}:=\{e_1,\ldots ,e_m\}\subset {\mathbb {Y}}$ of ${\mathbb {Y}}$, then it is also strongly asymptotically regular as well as metrically subregular in direction *u* at $({\bar{x}}, {\bar{y}})$.

##### Proof

Fix arbitrary sequences $\{(x_k,y_k)\}_{k\in {\mathbb {N}}}\subset {\text {gph}}\varPhi$, $\{x_k^*\}_{k\in {\mathbb {N}}}\subset {\mathbb {X}}$, and $\{\lambda _k\}_{k\in {\mathbb {N}}}\subset {\mathbb {Y}}$ as well as $x^*\in {\mathbb {X}}$ and $y^*\in {\mathbb {Y}}$ satisfying $x_k\notin \varPhi ^{-1}({\bar{y}})$, $y_k\ne {\bar{y}}$, and $x_k^*\in {\widehat{D}}^*\varPhi (x_k,y_k)(\lambda _k)$ for each $k\in {\mathbb {N}}$ as well as the convergences (5.1). Let us define $w_k:=(y_k-{\bar{y}})/\left\| y_k-{\bar{y}}\right\|$ and ${\tilde{\lambda }}_k:=\lambda _k/\left\| \lambda _k\right\|$ for each $k\in {\mathbb {N}}$. The requirements from (5.1) imply that $\{w_k\}_{k\in {\mathbb {N}}}$ and $\{{\tilde{\lambda }}_k\}_{k\in {\mathbb {N}}}$ converge, along a subsequence (without relabeling), to the same nonvanishing limit which we will call $\lambda \in {\mathbb {S}}_{{\mathbb {Y}}}$. Moreover, given $i\in \{1,\ldots ,m\}$ with $\langle \lambda , e_i\rangle \ne 0$, for sufficiently large $k\in {\mathbb {N}}$, we get $\langle w_{k}, e_i\rangle \ne 0$ and

$$\begin{aligned} 0 < \langle \lambda , e_i\rangle \langle w_{k}, e_i\rangle = \langle \lambda , e_i\rangle \langle y_k - {\bar{y}}, e_i\rangle /\left\| y_k - {\bar{y}}\right\| . \end{aligned}$$

Observing that we have $x_k^*/\left\| \lambda _k\right\| \rightarrow 0$ from (5.1), we find $\lambda \in \ker D^*\varPhi (({\bar{x}},{\bar{y}});(u,0))$ by definition of the directional limiting coderivative. This contradicts validity of quasi-normality of $\varPhi$ at $({\bar{x}},{\bar{y}})$ in direction *u* w.r.t. ${\mathcal {E}}$. Particularly, such sequences $\{(x_k,y_k)\}_{k\in {\mathbb {N}}}$, $\{x_k^*\}_{k\in {\mathbb {N}}}$, and $\{\lambda _k\}_{k\in {\mathbb {N}}}$ cannot exist which means that $\varPhi$ is strongly asymptotically regular in direction *u* at $({\bar{x}},{\bar{y}})$.

The claim about metric subregularity now follows from [37, Corollary 1], since the only difference from quasi-normality is the requirement

$$\begin{aligned} \langle \lambda _k/\Vert \lambda _k\Vert , (y_k-{\bar{y}})/\Vert y_k-{\bar{y}}\Vert \rangle \rightarrow 1 \end{aligned}$$

which is the same as $(y_k-{\bar{y}})/\Vert y_k-{\bar{y}}\Vert - \lambda _k/\Vert \lambda _k\Vert \rightarrow 0$ as mentioned in the comments after Corollary 4.5. $\square$

Relying on this result, [36, Theorem 7] yields that directional pseudo- and quasi-normality provide constraint qualifications for (P) which ensure validity of directional M-stationarity at local minimizers.

We would like to point the reader’s attention to the fact that nondirectional versions of pseudo- and quasi-normality are not comparable with the nondirectional version of asymptotic regularity. This has been observed in the context of standard nonlinear programming, see [5, Sections 4.3, 4.4]. The reason is that the standard version of asymptotic regularity makes no use of the multiplier information (4.17c).

In [22, Section 4.2], which is a preprint version of this paper, our new notions of directional pseudo- and quasi-normality from Definition 5.2 are worked out for so-called optimization problem with equilibrium constraints which cover models with variational inequality constraints, see e.g. [32, 62, 68], or bilevel optimization problems, see e.g. [29, 30].

#### Directional pseudo- and quasi-normality for geometric constraint systems

Let us now also justify the terminology by showing that the new notions from Definition 5.2 coincide with directional pseudo- and quasi-normality in the case of standard constraint mappings as studied in [16].

We start with a general result relying on calmness of the constraint function. Note that we consider ${\bar{y}}:=0$ for simplicity of notation. Furthermore, we only focus on the concept of directional quasi-normality in our subsequently stated analysis. Analogous results hold for directional pseudo-normality.

##### Proposition 5.2

A constraint mapping $\varPhi :{\mathbb {X}}\rightrightarrows {\mathbb {Y}}$ given by $\varPhi (x):= g(x) - D$, $x\in {\mathbb {X}}$, where $g:{\mathbb {X}} \rightarrow {\mathbb {Y}}$ is a continuous function which is calm in direction $u \in {\mathbb {S}}_{{\mathbb {X}}}$ at ${\bar{x}}\in {\mathbb {X}}$ such that $({\bar{x}}, 0) \in {\text {gph}}\varPhi$ and $D \subset {\mathbb {Y}}$ is closed, is quasi-normal in direction *u* at $({\bar{x}}, 0)$ w.r.t. some orthonormal basis ${\mathcal {E}}:=\{e_1,\ldots ,e_m\}\subset {\mathbb {Y}}$ of ${\mathbb {Y}}$ provided there do not exist a direction $v \in {\mathbb {Y}}$ and a nonzero vector $\lambda \in {\mathcal {N}}_D(g({\bar{x}});v)$ with $0 \in D^*g({\bar{x}};(u,v))(\lambda )$ satisfying the following condition: there are sequences $\{x_k\}_{k\in {\mathbb {N}}}\subset {\mathbb {X}}$ with $x_k\ne {\bar{x}}$ for all $k\in {\mathbb {N}}$, $\{z_k\}_{k\in {\mathbb {N}}}\subset D$, $\{\lambda _k\}_{k\in {\mathbb {N}}}\subset {\mathbb {Y}}$, and $\{\eta _k\}_{k\in {\mathbb {N}}}\subset {\mathbb {X}}$ satisfying $x_k\rightarrow {\bar{x}}$, $z_k\rightarrow g({\bar{x}})$, $\lambda _k\rightarrow \lambda$, $\eta _k\rightarrow 0$,

5.3 $$\begin{aligned} \frac{x_k-{\bar{x}}}{\left\| x_k-{\bar{x}}\right\| }\rightarrow u, \qquad \frac{z_k-g({\bar{x}})}{\left\| x_k-{\bar{x}}\right\| }\rightarrow v, \qquad \frac{g(x_k)-g({\bar{x}})}{\left\| x_k-{\bar{x}}\right\| }\rightarrow v, \end{aligned}$$

and, for all $k\in {\mathbb {N}}$ and $i\in \{1,\ldots ,m\}$, $\eta _k \in {\widehat{D}}^*g(x_k)(\lambda _k)$, $\lambda _k\in \widehat{{\mathcal {N}}}_D(z_k)$, as well as $\langle \lambda , e_i\rangle \langle g(x_k)-z_k, e_i\rangle >0$ if $\langle \lambda , e_i\rangle \ne 0$.

Moreover, if *g* is even calm near ${\bar{x}}$, the two conditions are equivalent.

##### Proof

$[\Longleftarrow ]$ Choose $\lambda \in \ker D^*\varPhi (({\bar{x}},0);(u,0))$ and sequences $\{(x_k,y_k)\}_{k\in {\mathbb {N}}}\subset {\text {gph}}\varPhi$ with $x_k\ne {\bar{x}}$ for all $k\in {\mathbb {N}}$ and $\{\lambda _k\}_{k\in {\mathbb {N}}}\subset {\mathbb {Y}}$, $\{\eta _k\}_{k\in {\mathbb {N}}}\subset {\mathbb {X}}$ satisfying (5.2) with ${\bar{y}}:=0$ and, for all $k\in {\mathbb {N}}$ and $i\in \{1,\ldots ,m\}$, $\eta _k\in {\widehat{D}}^*\varPhi (x_k,y_k)(\lambda _k)$ as well as $\langle \lambda , e_i\rangle \langle y_{k}, e_i\rangle >0$ if $\langle \lambda , e_i\rangle \ne 0$. Applying Lemma 3.1 (a) yields $\eta _k \in {\widehat{D}}^*g(x_k)(\lambda _k)$ and $\lambda _k\in \widehat{{\mathcal {N}}}_D(g(x_k) - y_k)$ for each $k\in {\mathbb {N}}$. The assumed calmness of *g* at ${\bar{x}}$ in direction *u* yields boundedness of the sequence $\{(g(x_k) - g({\bar{x}}))/\left\| x_k - {\bar{x}}\right\| \}_{k\in {\mathbb {N}}}$, i.e., along a subsequence (without relabeling) it converges to some $v\in {\mathbb {Y}}$. Note also that $(u,v)\in {\mathcal {T}}_{{\text {gph}}g}({\bar{x}},g({\bar{x}}))$, i.e., $v\in Dg({\bar{x}})(u)$, and that $\{(x_k,g(x_k))\}_{k\in {\mathbb {N}}}$ converges to $({\bar{x}},g({\bar{x}}))$ from direction (*u*, *v*). Setting $z_k:= g(x_k) - y_k$ for each $k\in {\mathbb {N}}$, we get $z_k \rightarrow g({\bar{x}})$ by continuity of *g* as well as $\lambda _k\in \widehat{{\mathcal {N}}}_D(z_k)$ and $\langle \lambda , e_i\rangle \langle g(x_k)-z_k, e_i\rangle >0$ if $\langle \lambda , e_i\rangle \ne 0$ for each $k\in {\mathbb {N}}$ and $i\in \{1,\ldots ,m\}$. Moreover, we have

$$\begin{aligned} \frac{z_k - g({\bar{x}})}{\left\| x_k-{\bar{x}}\right\| } = \frac{g(x_k)- g({\bar{x}})}{\left\| x_k-{\bar{x}}\right\| } - \frac{y_k}{\left\| x_k-{\bar{x}}\right\| } \rightarrow v - 0 = v \end{aligned}$$

and $v\in {\mathcal {T}}_D(g({\bar{x}}))$ follows as well. Finally, taking the limit yields $\lambda \in {\mathcal {N}}_D(g({\bar{x}});v)$ and $0 \in D^*g({\bar{x}};(u,v))(\lambda )$, so that the assumptions of the proposition imply $\lambda =0$. Consequently, $\varPhi$ is quasi-normal in direction *u* at $({\bar{x}},0)$ w.r.t. ${\mathcal {E}}$.

$[\Longrightarrow ]$ Assume that quasi-normality in direction *u* holds at $({\bar{x}},0)$ w.r.t. ${\mathcal {E}}$ and that *g* is calm around ${\bar{x}}$. Suppose that there are some $v \in {\mathbb {Y}}$, $\lambda \in {\mathcal {N}}_D(g({\bar{x}});v)$ with $0 \in D^*g({\bar{x}};(u,v))(\lambda )$, and sequences $\{x_k\}_{k\in {\mathbb {N}}}\subset {\mathbb {X}}$ with $x_k\ne {\bar{x}}$ for all $k\in {\mathbb {N}}$ and $\{z_k\}_{k\in {\mathbb {N}}}\subset D$, $\{\lambda _k\}_{k\in {\mathbb {N}}}\subset {\mathbb {Y}}$, $\{\eta _k\}_{k\in {\mathbb {N}}}\subset {\mathbb {X}}$ with $x_k\rightarrow {\bar{x}}$, $z_k\rightarrow g({\bar{x}})$, $\lambda _k\rightarrow \lambda$, $\eta _k \rightarrow 0$, (5.3), and, for all $k\in {\mathbb {N}}$ and $i\in \{1,\ldots ,m\}$, $\eta _k \in {\widehat{D}}^*g(x_k)(\lambda _k)$, $\lambda _k\in \widehat{{\mathcal {N}}}_D(z_k)$, as well as $\langle \lambda , e_i\rangle \langle g(x_k)-z_k, e_i\rangle >0$ as soon as $\langle \lambda , e_i\rangle \ne 0$. Set $y_k:=g(x_k)-z_k$ for each $k\in {\mathbb {N}}$. Then we have $y_k\rightarrow 0$,

$$\begin{aligned} \frac{y_k}{\left\| x_k-{\bar{x}}\right\| } = \frac{g(x_k)-z_k}{\left\| x_k-{\bar{x}}\right\| } = \frac{g(x_k)-g({\bar{x}})}{\left\| x_k-{\bar{x}}\right\| } -\frac{z_k-g({\bar{x}})}{\left\| x_k-{\bar{x}}\right\| } \rightarrow v - v = 0, \end{aligned}$$

and, for all $k\in {\mathbb {N}}$ and $i\in \{1,\ldots ,m\}$, $\lambda _k\in \widehat{{\mathcal {N}}}_D(g(x_k)-y_k)$ as well as $\langle \lambda , e_i\rangle \langle y_{k}, e_i\rangle >0$ if $\langle \lambda , e_i\rangle \ne 0$. Since $\eta _k \in {\widehat{D}}^*g(x_k)(\lambda _k)$, calmness of *g* at $x_k$ implies $\eta _k\in {\widehat{D}}^*\varPhi (x_k,y_k)(\lambda _k)$ due to Lemma 3.1 (a), and taking the limit yields $\lambda \in \ker D^*\varPhi (({\bar{x}},{\bar{y}});(u,0))$. Thus, the assumed quasi-normality of $\varPhi$ at $({\bar{x}},0)$ in direction *u* w.r.t. ${\mathcal {E}}$ yields $\lambda =0$ and the claim follows. $\square$

If *g* is continuously differentiable, the situation becomes a bit simpler and we precisely recover the notion of directional quasi-normality for geometric constraint systems as discussed in [16, Definition 3.4].

##### Corollary 5.2

A constraint mapping $\varPhi :{\mathbb {X}}\rightrightarrows {\mathbb {Y}}$ given by $\varPhi (x) = g(x) - D$, $x\in {\mathbb {X}}$, where $g:{\mathbb {X}} \rightarrow {\mathbb {Y}}$ is continuously differentiable and $D \subset {\mathbb {Y}}$ is closed, is quasi-normal in direction $u \in {\mathbb {S}}_{{\mathbb {X}}}$ at $({\bar{x}}, 0) \in {\text {gph}}\varPhi$ w.r.t. some orthonormal basis $\{e_1,\ldots ,e_m\}\subset {\mathbb {Y}}$ of ${\mathbb {Y}}$ if and only if there does not exist a nonzero vector $\lambda \in {\mathcal {N}}_D(g({\bar{x}});\nabla g({\bar{x}}) u)$ with $\nabla g({\bar{x}})^*\lambda =0$ satisfying the following condition: there are sequences $\{x_k\}_{k\in {\mathbb {N}}}\subset {\mathbb {X}}$ with $x_k\ne {\bar{x}}$ for all $k\in {\mathbb {N}}$, $\{z_k\}_{k\in {\mathbb {N}}}\subset D$, and $\{\lambda _k\}_{k\in {\mathbb {N}}}\subset {\mathbb {Y}}$ satisfying $x_k\rightarrow {\bar{x}}$, $z_k\rightarrow g({\bar{x}})$, $\lambda _k\rightarrow \lambda$,

5.4 $$\begin{aligned} \frac{x_k-{\bar{x}}}{\left\| x_k-{\bar{x}}\right\| }\rightarrow u, \qquad \frac{z_k-g({\bar{x}})}{\left\| x_k-{\bar{x}}\right\| }\rightarrow \nabla g({\bar{x}})u, \end{aligned}$$

and, for all $k\in {\mathbb {N}}$ and $i\in \{1,\ldots ,m\}$, $\lambda _k\in \widehat{{\mathcal {N}}}_D(z_k)$ and $\langle \lambda , e_i\rangle \langle g(x_k)-z_k, e_i\rangle >0$ if $\langle \lambda , e_i\rangle \ne 0$.

In [16, Section 3.3], it has been reported that under additional conditions on the set *D*, we can drop the sequences $\{z_k\}_{k\in {\mathbb {N}}}$ and $\{\lambda _k\}_{k\in {\mathbb {N}}}$ from the characterization of directional quasi-normality in Corollary 5.2. Particularly, this can be done for so-called *ortho-disjunctive* programs which cover, e.g., standard nonlinear, complementarity-, cardinality-, or switching-constrained optimization problems. In this regard, Corollary 5.2 reveals that some results from [24, 46, 52, 54, 61] are covered by our general concept from Definition 5.2.

Let us briefly compare our results with the approach from [15].

##### Remark 5.3

Let us consider the setting discussed in Proposition 5.2. The directional versions of pseudo- and quasi-normality from [15] operate with all nonzero pairs of directions (*u*, *v*), rather than just a fixed *u*. The advantage is that calmness of *g* plays no role. The reason is, however, that the authors in [15] only derive statements regarding metric subregularity, but not metric subregularity in some fixed direction. Calmness of *g* is needed precisely for preservation of directional information. We believe that it is useful to know how to verify if a mapping is metrically subregular in a specific direction since only some directions play a role in many situations. We could drop the calmness assumption from Proposition 5.2, but, similarly as in [18, Theorem 3.1], additional directions of the type (0, *v*) for a nonzero *v* would appear. Clearly, such directions are included among all nonzero pairs (*u*, *v*), but the connection to the original direction *u* would have been lost.

### Sufficient conditions for asymptotic regularity via pseudo-coderivatives

#### The role of super-coderivatives

We start this section by interrelating the concept of super-coderivatives from Definition 2.4 and asymptotic regularity. Fix $({\bar{x}},{\bar{y}})\in {\text {gph}}\varPhi$ and choose $\{(x_k,y_k)\}_{k\in {\mathbb {N}}}\subset {\text {gph}}\varPhi$, $\{x_k^*\}_{k\in {\mathbb {N}}}\subset {\mathbb {X}}$, and $\{\lambda _k\}_{k\in {\mathbb {N}}}\subset {\mathbb {Y}}$ as well as $x^*\in {\mathbb {X}}$ and $y^*\in {\mathbb {Y}}$ satisfying $x_k\notin \varPhi ^{-1}({\bar{y}})$, $y_k\ne {\bar{y}}$, and $x_k^*\in {\widehat{D}}^*\varPhi (x_k,y_k)(\lambda _k)$ for all $k\in {\mathbb {N}}$ as well as the convergences (5.1). For each $k\in {\mathbb {N}}$, we set $t_k:=\left\| x_k-{\bar{x}}\right\|$, $\tau _k:=\left\| y_k-{\bar{y}}\right\|$,

$$\begin{aligned} u_k:=\frac{x_k-{\bar{x}}}{\left\| x_k-{\bar{x}}\right\| },\qquad v_k:=\frac{y_k-{\bar{y}}}{\left\| y_k-{\bar{y}}\right\| },\qquad y_k^*:=\frac{\left\| y_k-{\bar{y}}\right\| }{\left\| x_k-{\bar{x}}\right\| }\lambda _k, \end{aligned}$$

and find $\tau _k/t_k\rightarrow 0$ as well as

$$\begin{aligned} \forall k\in {\mathbb {N}}:\quad x_k^*\in {\widehat{D}}^*\varPhi ({\bar{x}}+t_ku_k,{\bar{y}}+\tau _kv_k)((t_k/\tau _k)y_k^*). \end{aligned}$$

Along a subsequence (without relabeling), $v_k\rightarrow v$ holds for some $v\in {\mathbb {S}}_{{\mathbb {Y}}}$. Thus, taking the limit $k\rightarrow \infty$, we have $x^*\in D^*_sup \varPhi (({\bar{x}},{\bar{y}});(u,v))(y^*)$ by definition of the super-coderivative. Moreover, from (5.1) we also know that $y^* = \left\| y^*\right\| v$. Consequently, we come up with the following lemma.

##### Lemma 5.2

Let $({\bar{x}},{\bar{y}})\in {\text {gph}}\varPhi$ and $u\in {\mathbb {S}}_{{\mathbb {X}}}$ be fixed. If

$$\begin{aligned} \bigcup _{v\in {\mathbb {S}}_{{\mathbb {Y}}}} D^*_sup \varPhi (({\bar{x}},{\bar{y}});(u,v))(\beta v) \subset {\text {Im}}D^*\varPhi ({\bar{x}},{\bar{y}}) \end{aligned}$$

holds for all $\beta \ge 0$, then $\varPhi$ is asymptotically regular at $({\bar{x}},{\bar{y}})$ in direction *u*. If the above estimate holds for all $\beta \ge 0$ with ${\text {Im}}D^*\varPhi ({\bar{x}},{\bar{y}})$ replaced by ${\text {Im}}D^*\varPhi (({\bar{x}},{\bar{y}});(u,0))$, then $\varPhi$ is strongly asymptotically regular at $({\bar{x}},{\bar{y}})$ in direction *u*.

The next result, which is based on hypothesis $A^\gamma (u)$, see Assumption 4.1, follows as a corollary of Lemmas 2.9 and 5.2, and gives new sufficient conditions for directional asymptotic regularity. Note that strong directional asymptotic regularity can be handled analogously by employing an adjusted version of $A^\gamma (u)$ where ${\text {Im}}D^*\varPhi ({\bar{x}},{\bar{y}})$ in the right-hand side of (4.15) is replaced by ${\text {Im}}D^*\varPhi (({\bar{x}},{\bar{y}});(u,0))$.

##### Theorem 5.2

Let $({\bar{x}},{\bar{y}})\in {\text {gph}}\varPhi$, $u\in {\mathbb {S}}_{{\mathbb {X}}}$, and $\gamma >1$ be fixed. If $A^\gamma (u)$ holds, then $\varPhi$ is asymptotically regular at $({\bar{x}},{\bar{y}})$ in direction *u*.

In the case where the pseudo-coderivatives involved in the construction of $A^\gamma (u)$ can be computed or estimated from above, new applicable sufficient conditions for (strong) directional asymptotic regularity are provided by Theorem 5.2. Particularly, in situations where $\varPhi$ is given in form of a constraint mapping and $\gamma :=2$ is fixed, we can rely on the results obtained in Sect. 3 in order to make the findings of Theorem 5.2 more specific. This will be done in the next subsection.

#### The case of constraint mappings

Throughout the section, we assume that $\varPhi :{\mathbb {X}}\rightrightarrows {\mathbb {Y}}$ is given by $\varPhi (x):=g(x)-D$, $x\in {\mathbb {X}}$, where $g:{\mathbb {X}}\rightarrow {\mathbb {Y}}$ is a twice continuously differentiable function and $D\subset {\mathbb {Y}}$ is a closed set. Furthermore, for simplicity of notation, we fix ${\bar{y}}:=0$ which is not restrictive as already mentioned earlier.

We start with a general result which does not rely on any additional structure of the set *D*.

##### Theorem 5.3

Let $({\bar{x}},0)\in {\text {gph}}\varPhi$ as well as $u\in {\mathbb {S}}_{{\mathbb {X}}}$ be fixed. Assume that (3.9) holds, as well as (3.10) or, in the case $\nabla g({\bar{x}})u\ne 0$, (3.11). If, for each $x^*\in {\mathbb {X}}$ and $y^*,z^*\in {\mathbb {Y}}$ satisfying

5.5a $$\begin{aligned} x^*&= \nabla ^2\langle y^*, g\rangle ({\bar{x}})(u) + \nabla g({\bar{x}})^*z^*, \end{aligned}$$

5.5b $$\begin{aligned} y^*&\in {\mathcal {N}}_D(g({\bar{x}});\nabla g({\bar{x}})u) \cap \ker \nabla g({\bar{x}})^*, \end{aligned}$$

5.5c $$\begin{aligned} z^*&\in D{\mathcal {N}}_D(g({\bar{x}}),y^*)(\nabla g({\bar{x}})u), \end{aligned}$$

there is some $\lambda \in {\mathcal {N}}_D(g({\bar{x}}))$ such that $x^*=\nabla g({\bar{x}})^*\lambda$, then $\varPhi$ is asymptotically regular at $({\bar{x}},0)$ in direction *u*. Moreover, $\varPhi$ is even strongly asymptotically regular at $({\bar{x}},0)$ in direction *u* if $\lambda$ can be chosen from ${\mathcal {N}}_D(g({\bar{x}});\nabla g({\bar{x}})u)$.

##### Proof

Theorem 3.1 (a) implies $\ker {{\widetilde{D}}}^*_2\varPhi (({\bar{x}},0);(u,0))=\{0\}$ (and so, due to (2.7), also $\ker D^*_2\varPhi (({\bar{x}},0);(u,0))=\{0\}$) as well as that for $x^*\in {\text {Im}}{{\widetilde{D}}}^*_2\varPhi (({\bar{x}},0);(u,0))$, we find $y^*,z^*\in {\mathbb {Y}}$ satisfying (5.5). The assumptions guarantee that we can find $\lambda \in {\mathcal {N}}_D(g({\bar{x}}))$ such that $x^*=\nabla g({\bar{x}})^*\lambda \in {\text {Im}}D^*\varPhi ({\bar{x}},0)$ where we used Lemma 3.1 (b). It follows ${\text {Im}}{{\widetilde{D}}}^*_2\varPhi (({\bar{x}},0);(u,0))\subset {\text {Im}}D^*\varPhi ({\bar{x}},0)$. Thus, Theorem 5.2 shows that $\varPhi$ is asymptotically regular at $({\bar{x}},0)$ in direction *u*. The statement regarding strong asymptotic regularity follows in analogous way while respecting Lemma 3.1 (c). $\square$

We note that (3.10) is stronger than (3.11) when $\nabla g({\bar{x}})u\ne 0$ holds, see (2.3). Naturally, this means that it is sufficient to check (3.10) regardless whether $\nabla g({\bar{x}})u$ vanishes or not. In the case $\nabla g({\bar{x}})u\ne 0$, however, it is already sufficient to check the milder condition (3.11). This will be important later on, see Proposition 5.3 and Remark 5.4 below.

Note also that we implicitly relied on condition (4.16) (with ${\bar{y}}:=0$ and $\gamma :=2$) in the proof of Theorem 5.3, and not on the milder refined condition (4.15) (again with ${\bar{y}}:=0$ and $\gamma :=2$) which appears in the statement of $A^\gamma (u)$. This happened due to the generality of the setting in Theorem 5.3. In the polyhedral situation, (4.15) can be employed to obtain the following improved result.

##### Theorem 5.4

Let $({\bar{x}},0)\in {\text {gph}}\varPhi$ as well as $u\in {\mathbb {S}}_{{\mathbb {X}}}$ be fixed. Let ${\mathbb {Y}}:={\mathbb {R}}^m$ and let *D* be polyhedral locally around $g({\bar{x}})$. Assume that condition (3.13) holds for each $s\in {\mathbb {X}}$. If, for each $x^*,s\in {\mathbb {X}}$, $y^*,z^*\in {\mathbb {R}}^m$, and $\alpha \ge 0$ satisfying (5.5a) and

5.6 $$\begin{aligned} \begin{aligned} y^*&\in {\mathcal {N}}_{{\textbf{T}}(u)}(w_s(u,v)) \cap \ker \nabla g({\bar{x}})^*, \\ z^*&\in {\mathcal {N}}_{{\textbf{T}}(u)}(w_s(u,v)) \quad \big ( \text {or } \ z^* \, \in {\mathcal {T}}_{ {\mathcal {N}}_{{\textbf{T}}(u)}(w_s(u,v)) } (y^*) \big ), \end{aligned} \end{aligned}$$

where $v:=\alpha y^*$, and ${\textbf{T}}(u)$ as well as $w_s(u,v)$ have been defined in (3.7), there is some $\lambda \in {\mathcal {N}}_D(g({\bar{x}}))$ such that $x^*=\nabla g({\bar{x}})^*\lambda$, then $\varPhi$ is asymptotically regular at $({\bar{x}},0)$ in direction *u*. Moreover, $\varPhi$ is even strongly asymptotically regular at $({\bar{x}},0)$ in direction *u* if $\lambda$ can be chosen from ${\mathcal {N}}_{{\mathcal {T}}_D(g({\bar{x}}))}(\nabla g({\bar{x}})u)$.

##### Proof

Due to Theorem 3.2, (3.13) yields $\ker D^*_2\varPhi (({\bar{x}},0);(u,0))\subset \{0\}$ in the present situation. Now, fix $x^*\in {{\widetilde{D}}}^*_2\varPhi (({\bar{x}},0);(u,0))(0)$. Then Theorem 3.1 (b) shows the existence of $z^*\in {\mathcal {N}}_{{\mathcal {T}}_D(g({\bar{x}}))}(\nabla g({\bar{x}})u)$ such that $x^*=\nabla g({\bar{x}})^*z^*$. Let us now consider the case $x^*\in D^*_2\varPhi (({\bar{x}},0);(u,{\bar{\alpha }} w))({\bar{\beta }} w)$ for some $w\in {\mathbb {S}}_{{\mathbb {R}}^m}$ and ${\bar{\alpha }},{\bar{\beta }} \ge 0$. If ${\bar{\beta }}=0$ holds, we can employ (2.7) to find $x^*\in {{\widetilde{D}}}^*_2\varPhi (({\bar{x}},0);(u,0))(0)$ and, thus, the above argumentation applies. Thus, let us consider ${\bar{\beta }}>0$ and set $\alpha :={\bar{\alpha }}/{\bar{\beta }}$. Then we have $x^*\in D^*_2\varPhi (({\bar{x}},0);(u,v))(y^*)$ for $v=\alpha y^*$ with $v:={\bar{\alpha }} w$ and $y^*:={\bar{\beta }} w$. Theorem 3.2 implies the existence of $s\in {\mathbb {X}}$ such that (5.5a) and (5.6) hold with $v=\alpha y^*$. Now, the postulated assumptions guarantee the existence of $\lambda \in {\mathcal {N}}_{D}(g({\bar{x}}))$ such that $x^*=\nabla g({\bar{x}})^*\lambda$. Respecting Lemma 3.1 (b), this shows (4.15) with ${\bar{y}}:=0$ and $\gamma :=2$. Thus, Theorem 5.2 yields that $\varPhi$ is asymptotically regular at $({\bar{x}},0)$ in direction *u*. The statement regarding strong asymptotic regularity follows analogously. $\square$

Due to Corollary 5.1, Theorems 5.3 and 5.4 provide constraint qualifications for M-stationarity. Interestingly, one can easily check that the same conditions can also be obtained from Proposition 4.2 by demanding that any mixed-order stationary point is already M-stationary.

In the remaining part of the section, we prove that the assumptions of Theorem 5.3 are not stronger than FOSCMS(*u*) while the assumptions of Theorem 5.4 are strictly weaker than the so-called *Second-Order Sufficient Condition for Metric Subregularity* (SOSCMS) in direction *u*.

Given a point ${\bar{x}}\in {\mathbb {X}}$ with $({\bar{x}},0)\in {\text {gph}}\varPhi$, Lemma 3.1 (c) shows that the condition

$$\begin{aligned} u\in {\mathbb {S}}_{{\mathbb {X}}},\, \nabla g({\bar{x}})u\in {\mathcal {T}}_D(g({\bar{x}})),\, \nabla g({\bar{x}})^*y^*=0,\, y^*\in {\mathcal {N}}_D(g({\bar{x}});\nabla g({\bar{x}})u) \quad \Longrightarrow \quad y^*=0 \end{aligned}$$

equals FOSCMS in the current setting. In the case where *D* is locally polyhedral around $g({\bar{x}})$, the refined condition

$$\begin{aligned} \left. \begin{aligned}&u\in {\mathbb {S}}_{{\mathbb {X}}},\, \nabla g({\bar{x}})u\in {\mathcal {T}}_D(g({\bar{x}})),\, \nabla g({\bar{x}})^*y^*=0,\\&\nabla ^2\langle y^*, g\rangle ({\bar{x}})[u,u]\ge 0,\, y^*\in {\mathcal {N}}_D(g({\bar{x}});\nabla g({\bar{x}})u) \end{aligned} \right\} \quad \Longrightarrow \quad y^*=0, \end{aligned}$$

is referred to as SOSCMS in the literature. As these names suggest, both conditions are sufficient for metric subregularity of $\varPhi$ at $({\bar{x}},0)$, see [39, Corollary 1]. Particularly, they provide constraint qualifications for M-stationarity of local minimizers. Again, with the aid of Lemma 3.1 (c), one can easily check that

$$\begin{aligned} \nabla g({\bar{x}})^*y^*=0,\, y^*\in {\mathcal {N}}_D(g({\bar{x}});\nabla g({\bar{x}})u) \quad \Longrightarrow \quad y^*=0 \end{aligned}$$

equals FOSCMS(*u*) in the present setting, and

$$\begin{aligned} \nabla g({\bar{x}})^*y^*=0,\, \nabla ^2\langle y^*, g\rangle ({\bar{x}})[u,u]\ge 0,\, y^*\in {\mathcal {N}}_D(g({\bar{x}});\nabla g({\bar{x}})u) \quad \Longrightarrow \quad y^*=0 \end{aligned}$$

will be denoted by SOSCMS(*u*). Each of the conditions FOSCMS(*u*) and SOSCMS(*u*) is sufficient for metric subregularity of $\varPhi$ at $({\bar{x}},0)$ in direction *u*.

##### Proposition 5.3

Consider $({\bar{x}},0) \in {\text {gph}}\varPhi$ and $u\in {\mathbb {S}}_{{\mathbb {X}}}$. Under FOSCMS(*u*) all assumptions of Theorem 5.3 are satisfied.

##### Proof

Let $y^* \in {\mathcal {N}}_{D}(g({\bar{x}});\nabla g({\bar{x}}) u)$ be such that $\nabla g({\bar{x}})^* y^* = 0$. Then FOSCMS(*u*) yields $y^* = 0$ and so (3.9) is satisfied. Moreover, we only need to show the remaining assertions for $y^* = 0$.

Assume that $\nabla g({\bar{x}})u \ne 0$ holds. Suppose now that (3.11) is violated, i.e., there exists ${{\hat{z}}}^* \in D_{\text {sub}}{\mathcal {N}}_{D}(g({\bar{x}}),0)(q)$ for $q:=\nabla g({\bar{x}})u/\left\| \nabla g({\bar{x}})u\right\|$ with $\nabla g({\bar{x}})^*{{\hat{z}}}^*=0$. By Lemma 2.5 and FOSCMS(*u*), we thus get ${{\hat{z}}}^* = 0$ which is a contradition since ${{\hat{z}}}^* \in {\mathbb {S}}_{{\mathbb {Y}}}$ by Definition 2.2. Similarly, in the case $\nabla g({\bar{x}})u=0$, we can verify (3.10) which reduces to

$$\begin{aligned} \nabla g({\bar{x}})^*{{\hat{z}}}^*=0, {\hat{z}}^* \in D{\mathcal {N}}_{D}(g({\bar{x}}),0)(0) \quad \Longrightarrow \quad {{\hat{z}}}^*=0. \end{aligned}$$

Applying Lemma 2.5 again, we get ${\hat{z}}^* \in {\mathcal {N}}_D(g({\bar{x}}))$ which implies ${\hat{z}}^* = 0$ since FOSCMS(*u*) corresponds to the Mordukhovich criterion due to $\nabla g({\bar{x}})u = 0$. Thus, we have shown that (3.10) or, in the case $\nabla g({\bar{x}})u\ne 0$, (3.11) holds.

Validity of the last assumption follows immediately since $z^* \in {\mathcal {N}}_D(g({\bar{x}});\nabla g({\bar{x}})u)$ is obtained from Lemma 2.5, and so we can just take $\lambda := z^*$ due to $y^*=0$. $\square$

##### Remark 5.4

Note that for $u\in {\mathbb {S}}_{{\mathbb {X}}}$ satisfying $\nabla g({\bar{x}})u\ne 0$, we have the trivial upper estimate $D_{sub }{\mathcal {N}}_{D}(g({\bar{x}}),y^*)(\nabla g({\bar{x}})u/\left\| \nabla g({\bar{x}})u\right\| ) \subset D{\mathcal {N}}_{D}(g({\bar{x}}),y^*)(0)$. Hence, in Theorem 5.3, it is possible to replace validity of (3.10) or, in the case $\nabla g({\bar{x}})u\ne 0$, (3.11) by the slightly stronger assumption that (3.10) has to hold (even in the case $\nabla g({\bar{x}})u\ne 0$). However, we cannot show anymore that FOSCMS(*u*) is sufficient for this stronger assumption to hold, i.e., dropping directional information comes for a price.

##### Proposition 5.4

Let $({\bar{x}},0)\in {\text {gph}}\varPhi$ as well as $u\in {\mathbb {S}}_{{\mathbb {X}}}$ be fixed, let ${\mathbb {Y}}:={\mathbb {R}}^m$, and let *D* be polyhedral locally around $g({\bar{x}})$. If SOSCMS(*u*) is valid, then the assumptions of Theorem 5.4 are satisfied.

##### Proof

The key step is to realize that if $y^* \in {\mathcal {N}}_{{\textbf{T}}(u)}(w_s(u,v))\cap \ker \nabla g({\bar{x}})^*$ for some $s\in {\mathbb {X}}$ and $v \in {\mathbb {R}}^m$, then we get

$$\begin{aligned} \frac{1}{2} \nabla ^2\langle y^*,g\rangle ({\bar{x}})[u,u] = \langle w_s(u,v), y^*\rangle + \langle v, y^*\rangle = \langle v, y^* \rangle \end{aligned}$$

by Remark 3.3 and $\nabla g({\bar{x}})^* y^* = 0$, and $y^*\in {\mathcal {N}}_D(g({\bar{x}});\nabla g({\bar{x}})u)$ also holds, again by Remark 3.3.

Then (3.13) follows because for $y^* \in {\mathcal {N}}_{{\textbf{T}}(u)}(w_s(u,0))\cap \ker \nabla g({\bar{x}})^*$, the relation $\nabla ^2\langle y^*,g\rangle ({\bar{x}})[u,u] = 0$ is obtained, and SOSCMS(*u*) yields $y^* = 0$.

Next, for arbitrary $y^* \in {\mathcal {N}}_{{\textbf{T}}(u)}(w_s(u,v))\cap \ker \nabla g({\bar{x}})^*$ with $s\in {\mathbb {X}}$ and $v:=\alpha y^*$ for some $\alpha \ge 0$, we get $\nabla ^2\langle y^*,g\rangle ({\bar{x}})[u,u] = 2\langle v, y^* \rangle =2\alpha \Vert y^*\Vert ^2 \ge 0$, so SOSCMS(*u*) can still be applied to give $y^* = 0$. Now, we can always take $\lambda := z^*$ since $z^* \in {\mathcal {N}}_{{\textbf{T}}(u)}(w_s(u,v)) \subset {\mathcal {N}}_D(g({\bar{x}});\nabla g({\bar{x}})u)$. $\square$

We immediately arrive at the following corollary.

##### Corollary 5.3

The constraint mapping $\varPhi$ is strongly asymptotically regular at $({\bar{x}},0) \in {\text {gph}}\varPhi$ in direction $u\in {\mathbb {S}}_{{\mathbb {X}}}$ if FOSCMS(*u*) holds or if ${\mathbb {Y}}:={\mathbb {R}}^m$, *D* is locally polyhedral around $g({\bar{x}})$, and SOSCMS(*u*) holds.

The following example shows that our new conditions from Theorem 5.4 are in fact strictly milder than SOSCMS.

##### Example 5.5

Let $g:{\mathbb {R}}\rightarrow {\mathbb {R}}^2$ and $D \subset {\mathbb {R}}^2$ be given by $g(x):= (x,-x^2)$, $x\in {\mathbb {R}}$, and $D:=({\mathbb {R}}_+ \times {\mathbb {R}}) \cup ({\mathbb {R}}\times {\mathbb {R}}_+)$. Observe that *D* is a polyhedral set. We consider the constraint map $\varPhi :{\mathbb {R}}\rightrightarrows {\mathbb {R}}^2$ given by $\varPhi (x):=g(x)-D$, $x\in {\mathbb {R}}$. We note that $\varPhi ^{-1}(0)=[0,\infty )$ holds. Hence, fixing ${\bar{x}}:=0$, we can easily check that $\varPhi$ is metrically subregular at $({\bar{x}},0)$ in direction 1 but not in direction $-1$, i.e., FOSCMS and SOSCMS must be violated.

First, we claim that all the assumptions from Theorem 5.4 are satisfied for $u=\pm 1$. Taking into account Remark 3.3, it suffices to verify these assumptions for $w_s(u,v)$ replaced by 0. Let us fix $u=\pm 1$, $y^*, z^* \in {\mathcal {N}}_{{\mathcal {T}}_D(g({\bar{x}}))}(\nabla g({\bar{x}})u)$ such that $\nabla g({\bar{x}})^* y^* = 0$ and $\nabla ^2\langle y^*,g\rangle ({\bar{x}})(u)+\nabla g({\bar{x}})^* z^*=x^*$ for $x^* \in {\mathbb {R}}$. We have $\nabla g({\bar{x}})u=(u,0)$, $\nabla ^2\langle y^*,g\rangle ({\bar{x}})(u)=-2y_2^*u$, and

$$\begin{aligned} {\mathcal {N}}_{{\mathcal {T}}_D(g({\bar{x}}))}(\nabla g({\bar{x}})u) = {\left\{ \begin{array}{ll} \{0\} \times {\mathbb {R}}_- &{} u=-1,\\ \{(0,0)\} &{} u=1. \end{array}\right. } \end{aligned}$$

Thus, for $u=1$, we have $y^*=0$ regardless of $x^*$. Hence, condition (3.13) holds trivially and we can choose $\lambda :=z^*$ to find $x^* = \nabla g({\bar{x}})^*\lambda$ as well as $\lambda \in {\mathcal {N}}_{{\mathcal {T}}_D(g({\bar{x}}))}(\nabla g({\bar{x}})u)$. For $u=-1$, we get $y_1^*=z_1^*=0$ and $y^*_2\le 0$. Thus, if $x^*=0$, from $-2 y_2^*u + z_1^* = 0$ we deduce $y_2^*=0$, and (3.13) follows. For arbitrary $x^*\in {\mathbb {R}}$, we get $x^* = -2 y_2^*u + z_1^* = 2 y_2^* \le 0$ and we can choose $\lambda := (x^*,0) \in {\mathcal {N}}_D(g({\bar{x}}))$ to obtain $\nabla g({\bar{x}})^*\lambda =x^*$. Note, however, that $(x^*,0) \notin {\mathcal {N}}_{{\mathcal {T}}_D(g({\bar{x}}))}(\nabla g({\bar{x}})u) = \{0\} \times {\mathbb {R}}_-$ unless $x^* = 0$.

Regarding the assumptions of Theorem 5.3, let us just mention, without providing the details, that (3.10) and (3.11) fail since the graphical (sub)derivative is too large. Particularly, this clarifies that these assumptions are not necessary e.g. in the polyhedral setting, but not because they would be satisfied automatically.

## Concluding remarks

In this paper, we enriched the general concepts of asymptotic stationarity and regularity with the aid of tools from directional limiting variational analysis. Our central result Theorem 4.1 states that, even in the absence of any constraint qualification, local minimizers of a rather general optimization problem are M-stationary, mixed-order stationary in terms of a suitable pseudo-coderivative, or asymptotically stationary in a critical direction (of a certain order). By ruling out the last option, we were in position to distill new mixed-order necessary optimality conditions. Some novel upper estimates for the second-order directional pseudo-coderivative of constraint mappings were successfully employed to make these results fully explicit in the presence of geometric constraints. Our findings also gave rise to the formulation of directional notions of asymptotic regularity for set-valued mappings. These conditions have been shown to serve as constraint qualifications guaranteeing M-stationarity of local minimizers in nonsmooth optimization. We embedded these new qualification conditions into the landscape of constraint qualifications which are already known from the literature, showing that these conditions are comparatively mild. Noting that directional asymptotic regularity might be difficult to check in practice, we then focused on the derivation of applicable sufficient conditions for its validity. First, we suggested directional notions of pseudo- and quasi-normality for that purpose which have been shown to generalize related concepts for geometric constraint systems to arbitrary set-valued mappings. Second, with the aid of so-called super- and pseudo-coderivatives, sufficient conditions for the presence of directional asymptotic regularity for geometric constraint systems in terms of first- and second-order derivatives of the associated mapping as well as standard variational objects associated with the underlying set were derived. These sufficient conditions turned out to be not stronger than the First- and Second-Order Sufficient Condition for Metric Subregularity from the literature.

In this paper, we completely neglected to study the potential value of directional asymptotic regularity in numerical optimization which might be a promising topic of future research. Furthermore, it has been shown in [63] that nondirectional asymptotic regularity can be applied nicely as a qualification condition in the limiting variational calculus. Most likely, directional asymptotic regularity may play a similar role in the directional limiting calculus. Finally, it seems desirable to further develop the calculus for pseudo-coderivatives for mappings which possess a more difficult structure than constraint mappings.

## Missing proofs

### Proof of Lemma 2.8

We only verify the (more technical) assertion regarding Definition 2.3 (b) as the proof for the assertion which addresses Definition 2.3 (c) follows in similar (but slightly easier) fashion.

Thus, fix $x^*\in {\mathbb {X}}$ and $y^*\in {\mathbb {Y}}$ as well as $\{u_k\}_{k\in {\mathbb {N}}},\{x_k^*\}_{k\in {\mathbb {N}}}\subset {\mathbb {X}}$, $\{v_k\}_{k\in {\mathbb {N}}},\{y_k^*\}_{k\in {\mathbb {N}}}\subset {\mathbb {Y}}$, and $\{t_k\}_{k\in {\mathbb {N}}}\subset {\mathbb {R}}_+$ which satisfy $u_k\rightarrow u$, $v_k\rightarrow v$, $t_k\downarrow 0$, $x_k^*\rightarrow x^*$, $y_k^*\rightarrow y^*$, and

$$\begin{aligned} \forall k\in {\mathbb {N}}:\quad \left( x_k^*,-\frac{y_k^*}{(t_k\Vert u_k\Vert )^{\gamma -1}}\right) \in {\mathcal {N}}_{{\text {gph}}\varPhi }({\bar{x}}+t_ku_k,{\bar{y}}+(t_k\Vert u_k\Vert )^\gamma v_k). \end{aligned}$$

By definition of the limiting normal cone, for each $k\in {\mathbb {N}}$, we find $\{x_{k,\ell }\}_{\ell \in {\mathbb {N}}},\{x_{k,\ell }^*\}_{\ell \in {\mathbb {N}}}\subset {\mathbb {X}}$ and $\{y_{k,\ell }\}_{\ell \in {\mathbb {N}}},\{y_{k,\ell }^*\}_{\ell \in {\mathbb {N}}}\subset {\mathbb {Y}}$ such that $x_{k,\ell }\rightarrow {\bar{x}}+t_ku_k$, $y_{k,\ell }\rightarrow {\bar{y}}+(t_k\Vert u_k\Vert )^\gamma v_k$, $x_{k,\ell }^*\rightarrow x_k^*$, and $y_{k,\ell }^*\rightarrow y_k^*/(t_k\Vert u_k\Vert )^{\gamma -1}$ as $\ell \rightarrow \infty$ and $(x_{k,\ell }^*,-y_{k,\ell }^*)\in \widehat{{\mathcal {N}}}_{{\text {gph}}\varPhi }(x_{k,\ell },y_{k,\ell })$ for all $\ell \in {\mathbb {N}}$.

For each $k\in {\mathbb {N}}$, let us define sequences $\{u_{k,\ell }\}_{\ell \in {\mathbb {N}}}\subset {\mathbb {X}}$ and $\{v_{k,\ell }\}_{\ell \in {\mathbb {N}}},\{{{\hat{y}}}_{k,\ell }^*\}_{\ell \in {\mathbb {N}}}\subset {\mathbb {Y}}$ by means of

$$\begin{aligned} \forall \ell \in {\mathbb {N}}:\quad u_{k,\ell }:=\frac{x_{k,\ell }-{\bar{x}}}{t_k},\qquad v_{k,\ell }:=\frac{y_{k,\ell }-{\bar{y}}}{(t_k\Vert u_{k,\ell }\Vert )^\gamma },\qquad {{\hat{y}}}_{k,\ell }^*:=(t_k\Vert u_{k,\ell }\Vert )^{\gamma -1}y_{k,\ell }^*. \end{aligned}$$

This gives

A.1 $$\begin{aligned} \forall \ell \in {\mathbb {N}}:\quad \left( x_{k,\ell }^*,-\frac{{{\hat{y}}}_{k,\ell }^*}{(t_k\Vert u_{k,\ell }\Vert )^{\gamma -1}}\right) \in \widehat{{\mathcal {N}}}_{{\text {gph}}\varPhi } \bigl ({\bar{x}}+t_ku_{k,\ell },{\bar{y}}+(t_k\Vert u_{k,\ell }\Vert )^\gamma v_{k,\ell }\bigr ). \nonumber \\ \end{aligned}$$

Furthermore, we have the convergences $u_{k,\ell }\rightarrow u_k$, $v_{k,\ell }\rightarrow v_k$, and ${{\hat{y}}}_{k,\ell }^*\rightarrow y_k^*$ as $\ell \rightarrow \infty$ by construction. Thus, for each $k\in {\mathbb {N}}$, we find an index $\ell (k)\in {\mathbb {N}}$ such that

$$\begin{aligned}{} & {} \Vert u_{k,\ell (k)}-u_k\Vert \le \frac{1}{k},\quad \Vert v_{k,\ell (k)}-v_k\Vert \le \frac{1}{k},\\{} & {} \Vert x_{k,\ell (k)}^*-x_k^*\Vert \le \frac{1}{k},\quad \Vert {{\hat{y}}}_{k,\ell (k)}^*-y_k^*\Vert \le \frac{1}{k}. \end{aligned}$$

Let us set ${{\tilde{u}}}_k:=u_{k,\ell (k)}$, ${{\tilde{v}}}_k:=v_{k,\ell (k)}$, ${{\tilde{x}}}_k^*:=x_{k,\ell (k)}^*$, and ${{\tilde{y}}}_k^*:={{\hat{y}}}_{k,\ell (k)}^*$ for each $k\in {\mathbb {N}}$. The above estimates and $u_k\rightarrow u$, $v_k\rightarrow v$, $x_k^*\rightarrow x^*$, as well as $y_k^*\rightarrow y^*$ give ${{\tilde{u}}}_k\rightarrow u$, ${{\tilde{v}}}_k\rightarrow v$, ${{\tilde{x}}}_k^*\rightarrow x^*$, as well as ${{\tilde{y}}}_k^*\rightarrow y^*$. Additionally, (A.1) guarantees

$$\begin{aligned} \forall k\in {\mathbb {N}}:\quad \left( {{\tilde{x}}}_k^*,-\frac{{{\tilde{y}}}_k^*}{(t_k\Vert {{\tilde{u}}}_k\Vert )^{\gamma -1}}\right) \in \widehat{{\mathcal {N}}}_{{\text {gph}}\varPhi } \bigl ({\bar{x}}+t_k{{\tilde{u}}}_k,{\bar{y}}+(t_k\Vert {{\tilde{u}}}_k\Vert )^\gamma {{\tilde{v}}}_k\bigr ). \end{aligned}$$

By definition of the directional pseudo-coderivative, $x^*\in D^*_\gamma \varPhi (({\bar{x}},{\bar{y}});(u,v))(y^*)$, is obtained and this shows the claim. $\square$

### Proof of Proposition 5.1

For nondirectional asymptotic regularity, the proof is standard and follows from a simple diagonal sequence argument. The proof for strong directional asymptotic regularity parallels the one for directional asymptotic regularity which is presented below.

Since one implication is clear by definition of the regular and limiting coderivative, we only show the other one. Therefore, let $\varPhi$ be asymptotically regular at $({\bar{x}},{\bar{y}})$ in direction *u*. Let us fix sequences $\{(x_k,y_k)\}_{k\in {\mathbb {N}}}\subset {\text {gph}}\varPhi$, $\{x_k^*\}_{k\in {\mathbb {N}}}\subset {\mathbb {X}}$, and $\{\lambda _k\}_{k\in {\mathbb {N}}}\subset {\mathbb {Y}}$ as well as $x^*\in {\mathbb {X}}$ and $y^*\in {\mathbb {Y}}$ satisfying $x_k\notin \varPhi ^{-1}({\bar{y}})$, $y_k\ne {\bar{y}}$, and $x_k^*\in D^*\varPhi (x_k,y_k)(\lambda _k)$ for each $k\in {\mathbb {N}}$ as well as the convergences (5.1). For each $k\in {\mathbb {N}}$, we find sequences $\{(x_{k,\ell },y_{k,\ell })\}_{\ell \in {\mathbb {N}}}\subset {\text {gph}}\varPhi$, $\{x_{k,\ell }^*\}_{\ell \in {\mathbb {N}}}\subset {\mathbb {X}}$, and $\{\lambda _{k,\ell }\}_{\ell \in {\mathbb {N}}}\subset {\mathbb {Y}}$ with $x_{k,\ell }\rightarrow x_k$, $x_{k,\ell }^*\rightarrow x_k^*$, $y_{k,\ell }\rightarrow y_k$, and $\lambda _{k,\ell }\rightarrow \lambda _k$ as $\ell \rightarrow \infty$ as well as $x_{k,\ell }^*\in {{\widehat{D}}}^*\varPhi (x_{k,\ell },y_{k,\ell })(\lambda _{k,\ell })$ for each $\ell \in {\mathbb {N}}$. Observing that $\varPhi ^{-1}({\bar{y}})$ is closed, its complement is open so that $x_{k,\ell }\notin \varPhi ^{-1}({\bar{y}})$ holds for sufficiently large $\ell \in {\mathbb {N}}$. Furthermore, since $\left\| x_k-{\bar{x}}\right\| >0$ and $\left\| y_k-{\bar{y}}\right\| >0$ are valid, we can choose an index $\ell (k)\in {\mathbb {N}}$ so large such that the estimates

$$\begin{aligned} \begin{aligned} \Vert x_{k,\ell (k)}-x_k\Vert&<\frac{1}{k}\left\| x_k-{\bar{x}}\right\| ,&\quad \Vert x_{k,\ell (k)}^*-x_k^*\Vert&<\frac{1}{k},&\\ \Vert y_{k,\ell (k)}-y_k\Vert&<\frac{1}{k}\left\| y_k-{\bar{y}}\right\| ,&\quad \Vert \lambda _{k,\ell (k)}-\lambda _k\Vert&<\frac{1}{k}&\end{aligned} \end{aligned}$$

and $x_{k,\ell (k)}\notin \varPhi ^{-1}({\bar{y}})$ as well as $y_{k,\ell (k)}\ne {\bar{y}}$ are valid. For each $k\in {\mathbb {N}}$, we set ${{\tilde{x}}}_k:=x_{k,\ell (k)}$, ${{\tilde{x}}}_k^*:=x_{k,\ell (k)}^*$, ${{\tilde{y}}}_k:=y_{k,\ell (k)}$, and ${\tilde{\lambda }}_k:=\lambda _{k,\ell (k)}$. Clearly, we have ${{\tilde{x}}}_k\rightarrow {\bar{x}}$, ${{\tilde{y}}}_k\rightarrow {\bar{y}}$, ${{\tilde{x}}}_k^*\rightarrow x^*$, $\Vert {\tilde{\lambda }}_k\Vert \rightarrow \infty$, $\{({{\tilde{x}}}_k,{{\tilde{y}}}_k)\}_{k\in {\mathbb {N}}}\subset {\text {gph}}\varPhi$, and ${{\tilde{x}}}_k\notin \varPhi ^{-1}({\bar{y}})$, ${{\tilde{y}}}_k\ne {\bar{y}}$, as well as ${{\tilde{x}}}_k^*\in {{\widehat{D}}}^*\varPhi ({{\tilde{x}}}_k,{{\tilde{y}}}_k)({\tilde{\lambda }}_k)$ for each $k\in {\mathbb {N}}$ by construction. Furthermore, we find

$$\begin{aligned} \left\| {{\tilde{x}}}_k-{\bar{x}}\right\| \ge \left\| x_k-{\bar{x}}\right\| -\left\| {{\tilde{x}}}_k-x_k\right\| \ge \frac{k-1}{k}\left\| x_k-{\bar{x}}\right\| \end{aligned}$$

for each $k\in {\mathbb {N}}$. With the above estimates at hand, we obtain

$$\begin{aligned} \left\| \frac{x_k-{\bar{x}}}{\Vert x_k-{\bar{x}}\Vert } - \frac{{{\tilde{x}}}_k-{\bar{x}}}{\left\| {{\tilde{x}}}_k-{\bar{x}}\right\| } \right\|&= \left\| \frac{x_k-{{\tilde{x}}}_k}{\left\| x_k-{\bar{x}}\right\| } + ({{\tilde{x}}}_k-{\bar{x}}) \left( \frac{1}{\Vert x_k-{\bar{x}}\Vert }-\frac{1}{\Vert {{\tilde{x}}}_k-{\bar{x}}\Vert } \right) \right\| \\&\le \frac{\left\| x_k-{{\tilde{x}}}_k\right\| }{\left\| x_k-{\bar{x}}\right\| } + \frac{\left\| {{\tilde{x}}}_k-{\bar{x}}\right\| \left\| x_k-{{\tilde{x}}}_k\right\| }{\left\| x_k-{\bar{x}}\right\| \left\| {{\tilde{x}}}_k-{\bar{x}}\right\| } \le \frac{2}{k} \end{aligned}$$

and

A.2 $$\begin{aligned} \begin{aligned} \left\| \frac{y_k-{\bar{y}}}{\Vert x_k-{\bar{x}}\Vert } - \frac{{{\tilde{y}}}_k-{\bar{y}}}{\left\| {{\tilde{x}}}_k-{\bar{x}}\right\| } \right\|&= \left\| \frac{y_k-{{\tilde{y}}}_k}{\left\| x_k-{\bar{x}}\right\| } + ({{\tilde{y}}}_k-{\bar{y}}) \left( \frac{1}{\Vert x_k-{\bar{x}}\Vert }-\frac{1}{\Vert {{\tilde{x}}}_k-{\bar{x}}\Vert } \right) \right\| \\&\le \frac{\left\| y_k-{{\tilde{y}}}_k\right\| }{\left\| x_k-{\bar{x}}\right\| } + \frac{\left\| {{\tilde{y}}}_k-{\bar{y}}\right\| {\left\| x_k-{{\tilde{x}}}_k\right\| }}{\left\| x_k-{\bar{x}}\right\| \left\| {{\tilde{x}}}_k-{\bar{x}}\right\| } \\&\le \frac{1}{k}\frac{\left\| y_k-{\bar{y}}\right\| }{\left\| x_k-{\bar{x}}\right\| } + \frac{1}{k-1}\frac{\left\| {{\tilde{y}}}_k-y_k\right\| +\left\| y_k-{\bar{y}}\right\| }{\left\| x_k-{\bar{x}}\right\| } \\ {}&\le \left( \frac{1}{k} + \frac{1}{k(k-1)} + \frac{1}{k-1}\right) \frac{\left\| y_k-{\bar{y}}\right\| }{\left\| x_k-{\bar{x}}\right\| } \\&= \frac{2}{k-1}\frac{\left\| y_k-{\bar{y}}\right\| }{\left\| x_k-{\bar{x}}\right\| }, \end{aligned}\nonumber \\ \end{aligned}$$

so that, with the aid of (5.1), we find $({{\tilde{x}}}_k-{\bar{x}})/\left\| {{\tilde{x}}}_k-{\bar{x}}\right\| \rightarrow u$ as well as $({{\tilde{y}}}_k-{\bar{y}})/\left\| {{\tilde{x}}}_k-{\bar{x}}\right\| \rightarrow 0$. With the aid of (A.2),

$$\begin{aligned} \left\| \frac{\Vert {{\tilde{y}}}_k-{\bar{y}}\Vert }{\Vert {{\tilde{x}}}_k-{\bar{x}}\Vert } {\tilde{\lambda }}_k - \frac{\left\| y_k-{\bar{y}}\right\| }{\left\| x_k-{\bar{x}}\right\| }\lambda _k \right\|&\le \frac{\Vert {{\tilde{y}}}_k-{\bar{y}}\Vert }{\Vert {{\tilde{x}}}_k-{\bar{x}}\Vert } \Vert {\tilde{\lambda }}_k-\lambda _k\Vert + \left| \frac{\Vert {{\tilde{y}}}_k-{\bar{y}}\Vert }{\Vert {{\tilde{x}}}_k-{\bar{x}}\Vert } - \frac{\left\| y_k-{\bar{y}}\right\| }{\left\| x_k-{\bar{x}}\right\| } \right| \left\| \lambda _k\right\| \\&\le \frac{1}{k}\frac{\Vert {{\tilde{y}}}_k-{\bar{y}}\Vert }{\Vert {{\tilde{x}}}_k-{\bar{x}}\Vert } + \frac{2}{k-1}\frac{\left\| y_k-{\bar{y}}\right\| }{\left\| x_k-{\bar{y}}\right\| }\left\| \lambda _k\right\| \end{aligned}$$

is obtained, which gives ${\tilde{\lambda }}_k\Vert {{\tilde{y}}}_k-{\bar{y}}\Vert /\Vert {{\tilde{x}}}_k-{\bar{x}}\Vert \rightarrow y^*$. Similar as above, we find

$$\begin{aligned} \left\| \frac{{{\tilde{y}}}_k-{\bar{y}}}{\left\| {{\tilde{y}}}_k-{\bar{y}}\right\| } -\frac{y_k-{\bar{y}}}{\left\| y_k-{\bar{y}}\right\| }\right\| \le \frac{2}{k} \end{aligned}$$

and

$$\begin{aligned} \bigl \Vert {\tilde{\lambda }}_k/\Vert {\tilde{\lambda }}_k\Vert - \lambda _k/\left\| \lambda _k\right\| \bigr \Vert \le 2\Vert \lambda _k-{\tilde{\lambda }}_k\Vert /\left\| \lambda _k\right\| \le 2/(k\left\| \lambda _k\right\| ), \end{aligned}$$

so that (5.1) gives us

$$\begin{aligned} \lim \limits _{k\rightarrow \infty } \left( \frac{{{\tilde{y}}}_k-{\bar{y}}}{\left\| {{\tilde{y}}}_k-{\bar{y}}\right\| } -\frac{{\tilde{\lambda }}_k}{\Vert {\tilde{\lambda }}_k\Vert }\right) = \lim \limits _{k\rightarrow \infty } \left( \frac{y_k-{\bar{y}}}{\left\| y_k-{\bar{y}}\right\| } -\frac{\lambda _k}{\left\| \lambda _k\right\| }\right) = 0. \end{aligned}$$

Now, since $\varPhi$ is asymptotically regular at $({\bar{x}},{\bar{y}})$ in direction *u*, we obtain $x^*\in {\text {Im}}D^*\varPhi ({\bar{x}},{\bar{y}})$. $\square$

## References

[1] Adam, L., Červinka, M., Pištěk, M.: Normally admissible stratifications and calculation of normal cones to a finite union of polyhedral sets. Set-Valued Var. Anal. **24**, 207–229 (2016). 10.1007/s11228-015-0325-8

[2] Andreani, R., Gómez, W., Haeser, G., Mito, L.M., Ramos, A.: On optimality conditions for nonlinear conic programming. Math. Oper. Res. **47**(3), 2160–2185 (2021). 10.1287/moor.2021.1203

[3] Andreani, R., Haeser, G., Martínez, J.M.: On sequential optimality conditions for smooth constrained optimization. Optimization **60**(5), 627–641 (2011). 10.1080/02331930903578700

[4] Andreani, R., Haeser, G., Secchin, L.D., Silva, P.J.S.: New sequential optimality conditions for mathematical programs with complementarity constraints and algorithmic consequences. SIAM J. Optim. **29**(4), 3201–3230 (2019). 10.1137/18M121040X

[5] Andreani, R., Martínez, J.M., Ramos, A., Silva, P.J.S.: A cone-continuity constraint qualification and algorithmic consequences. SIAM J. Optim. **26**(1), 96–110 (2016). 10.1137/15M1008488

[6] Andreani, R., Martínez, J.M., Ramos, A., Silva, P.J.S.: Strict constraint qualifications and sequential optimality conditions for constrained optimization. Math. Oper. Res. **43**(3), 693–717 (2018). 10.1287/moor.2017.0879

[7] Andreani, R., Martínez, J.M., Svaiter, B.F.: A new sequential optimality condition for constrained optimization and algorithmic consequences. SIAM J. Optim. **20**(6), 3533–3554 (2010). 10.1137/090777189

[8] Arutyunov, A.V., Izmailov, A.F.: Covering on a convex set in the absence of Robinson’s regularity. SIAM J. Optim. **30**(1), 604–629 (2020). 10.1137/19M1256634

[9] Arutyunov, A.V., Avakov, E.R., Izmailov, A.F.: Necessary optimality conditions for constrained optimization problems under relaxed constraint qulifications. Math. Program. **114**, 37–68 (2008). 10.1007/s10107-006-0082-4

[10] Aubin, J.P., Frankowska, H.: Set-valued Analysis. Modern Birkhäuser Classics, Birkhäuser, Boston, (2009). 10.1007/978-0-8176-4848-0, reprint of the 1990 edition

[11] Avakov, E.R.: Extremum conditions for smooth problems with equality-type constraints. USSR Comput. Math. Math. Phys. **25**(3), 24–32 (1985). 10.1016/0041-5553(85)90069-2

[12] Avakov, E.R.: Necessary extremum conditions for smooth anormal problems with equality- and inequality-type constraints. Math. Notes Acad. Sci. USSR **45**, 431–437 (1989). 10.1007/BF01158229

[13] Avakov, E.R., Arutyunov, A.V., Izmailov, A.F.: Necessary conditions for an extremum in a mathematical programming problem. Proc. Stekalov Inst. Math. **256**, 2–25 (2007). 10.1134/S0081543807010014

[14] Bai, K., Ye, J.J.: Directional necessary optimality conditions for bilevel programs. Math. Oper. Res. **47**(2), 1169–1191 (2022). 10.1287/moor.2021.1164

[15] Bai, K., Ye, J.J., Zhang, J.: Directional quasi-/pseudo-normality as sufficient conditions for metric subregularity. SIAM J. Optim. **29**(4), 2625–2649 (2019). 10.1137/18M1232498

[16] Benko, M., Červinka, M., Hoheisel, T.: Sufficient conditions for metric subregularity of constraint systems with applications to disjunctive and ortho-disjunctive programs. Set-Valued Var. Anal. **30**, 1143–177 (2022). 10.1007/s11228-020-00569-7

[17] Benko, M., Gfrerer, H.: New verifiable stationarity concepts for a class of mathematical programs with disjunctive constraints. Optimization **67**(1), 1–23 (2018). 10.1080/02331934.2017.1387547 29375237 10.1080/02331934.2017.1387547 PMC5761710

[18] Benko, M., Gfrerer, H., Outrata, J.V.: Calculus for directional limiting normal cones and subdifferentials. Set-Valued Var. Anal. **27**(3), 713–745 (2019). 10.1007/s11228-018-0492-5

[19] Benko, M., Gfrerer, H., Ye, J.J., Zhang, J., Zhou, J.C.: Second-order optimality conditions for general nonconvex optimization problems and variational analysis of disjunctive systems. SIAM J. Optim. **33**(4), 2625–2653 (2023). 10.1137/22M1484742

[20] Benko, M., Mehlitz, P.: Calmness and calculus: two basic patterns. Set-Valued Var. Anal. **30**, 81–117 (2022a). 10.1007/s11228-021-00589-x

[21] Benko, M., Mehlitz, P.: On the directional asymptotic approach in optimization theory Part A: approximate, M-, and mixed-order stationarity. Tech. rep., preprint arXiv, (2022b) arxiv:2204.13932

[22] Benko, M., Mehlitz, P.: On the directional asymptotic approach in optimization theory Part B: constraint qualifications. Tech. rep., preprint arXiv, (2022c) arxiv:2205.00775 10.1007/s10107-024-02089-w PMC11735583 39830447

[23] Bertsekas, D., Nedić, A., Ozdaglar, A.E.: Convex analysis and optimization. Athena Scientific, Belmont (2003)

[24] Bertsekas, D.P., Ozdaglar, A.E.: Pseudonormality and a Lagrange multiplier theory for constrained optimization. J. Optim. Theory Appl. **114**, 287–343 (2002). 10.1023/A:1016083601322

[25] Bonnans, J.F., Shapiro, A.: Perturbation Analysis of Optimization Problems. Springer, New York (2000). 10.1007/978-1-4612-1394-9

[26] Börgens, E., Kanzow, C., Mehlitz, P., Wachsmuth, G.: New constraint qualifications for optimization problems in Banach spaces based on asymptotic KKT conditions. SIAM J. Optim. **30**(4), 2956–2982 (2020). 10.1137/19M1306804

[27] Burke, J.V.: Calmness and exact penalization. SIAM J. Control. Optim. **29**(2), 493–497 (1991). 10.1137/0329027

[28] Clarke, F.: Optimization and Nonsmooth Analysis. Wiley, New York (1983). 10.1137/1.9781611971309

[29] Dempe, S.: Foundations of Bilevel Programming. Kluwer, Dordrecht (2002). 10.1007/b101970

[30] Dempe, S., Kalashnikov, V., Pérez-Valdéz, G., Kalashnykova, N.: Bilevel Programming Problems - Theory. Algorithms and Applications to Energy Networks, Springer, Berlin (2015). 10.1007/978-3-662-45827-3

[31] Dontchev, A.L., Rockafellar, R.T.: Implicit Functions and Solution Mappings. Springer, Heidelberg (2014). 10.1007/978-0-387-87821-8

[32] Facchinei, F., Pang, J.S.: Finite-Dimensional Variational Inequalities and Complementarity Problems. Springer, New York (2003). 10.1007/b97543

[33] Fischer, A., Izmailov, A.F., Jelitte, M.: Newton-type methods near critical solutions of piecewise smooth nonlinear equations. Comput. Optim. Appl. **80**, 587–615 (2021). 10.1007/s10589-021-00306-2

[34] Fischer, A., Izmailov, A.F., Jelitte, M.: Behavior of Newton-type methods near critical solutions of nonlinear equations with semismooth derivatives. J. Optim. Theory Appl. (2023). 10.1007/s10957-023-02350-w

[35] Gfrerer, H.: Second-order necessary conditions for nonlinear optimization problems with abstract constraints: the degenerate case. SIAM J. Optim. **18**(2), 589–612 (2007). 10.1137/050641387

[36] Gfrerer, H.: On directional metric regularity, subregularity and optimality conditions for nonsmooth mathematical programs. Set-Valued Var. Anal. **21**(2), 151–176 (2013). 10.1007/s11228-012-0220-5

[37] Gfrerer, H.: On metric pseudo-(sub)regularity of multifunctions and optimality conditions for degenerated mathematical programs. Set-Valued Var. Anal. **22**(1), 79–115 (2014). 10.1007/s11228-013-0266-z

[38] Gfrerer, H.: Optimality conditions for disjunctive programs based on generalized differentiation with application to mathematical programs with equilibrium constraints. SIAM J. Optim. **24**(2), 898–931 (2014). 10.1137/130914449

[39] Gfrerer, H., Klatte, D.: Lipschitz and Hölder stability of optimization problems and generalized equations. Math. Program. **158**, 35–75 (2016). 10.1007/s10107-015-0914-1

[40] Gfrerer, H., Outrata, J.V.: On computation of limiting coderivatives of the normal-cone mapping to inequality systems and their applications. Optimization **65**(4), 671–700 (2016). 10.1080/02331934.2015.1066372

[41] Gfrerer, H., Ye, J.J.: New constraint qualifications for mathematical programs with equilibrium constraints via variational analysis. SIAM J. Optim. **27**(2), 842–865 (2017). 10.1137/16M1088752

[42] Gfrerer, H., Ye, J.J., Zhou, J.: Second-order optimality conditions for nonconvex set-constrained optimization problems. Math. Oper. Res. **47**(3), 2344–2365 (2022). 10.1287/moor.2021.1211

[43] Guo, L., Ye, J.J., Zhang, J.: Mathematical programs with geometric constraints in Banach spaces: enhanced optimality, exact penalty, and sensitivity. SIAM J. Optim. **23**(4), 2295–2319 (2013). 10.1137/130910956

[44] Haraux, A.: How to differentiate the projection on a convex set in Hilbert space. Some applications to variational inequalities. J. Math. Soc. Jpn. **29**(4), 615–631 (1977). 10.2969/jmsj/02940615

[45] Helou, E.S., Santos, S.A., Simões, L.E.A.: A new sequential optimality condition for constrained nonsmooth optimization. SIAM J. Optim. **30**(2), 1610–1637 (2020). 10.1137/18M1228608

[46] Hestenes, M.R.: Optimization Theory - the Finite-Dimensional Case. Wiley, New York (1975)

[47] Ioffe, A.D.: Regular points of Lipschitz functions. Trans. Am. Math. Soc. **251**, 61–69 (1979). 10.1090/S0002-9947-1979-0531969-6

[48] Ioffe, A.D.: Variational Analysis of Regular Mappings. Springer, Cham (2017). 10.1007/978-3-319-64277-2

[49] Izmailov, A.F., Kurennoy, A.S., Solodov, M.V.: Critical solutions of nonlinear equations: local attraction for Newton-type methods. Math. Program. **167**, 355–379 (2018). 10.1007/s10107-017-1128-5

[50] Izmailov, A.F., Solodov, M.V.: Complementarity constraint qualification via the theory of 2-regularity. SIAM J. Optim. **13**(2), 368–385 (2002). 10.1137/S1052623499365292

[51] Izmailov, A.F., Solodov, M.V.: The theory of 2-regularity for mappings with Lipschitzian derivatives and its applications to optimality conditions. Math. Oper. Res. **27**(3), 614–635 (2002). 10.1287/moor.27.3.614.308

[52] Kanzow, C., Raharja, A.B., Schwartz, A.: An augmented Lagrangian method for cardinality-constrained optimization problems. J. Optim. Theory Appl. **189**, 793–813 (2021). 10.1007/s10957-021-01854-7

[53] Kanzow, C., Raharja, A.B., Schwartz, A.: Sequential optimality conditions for cardinality-constrained optimization problems with applications. Comput. Optim. Appl. **80**, 185–211 (2021). 10.1007/s10589-021-00298-z

[54] Kanzow, C., Schwartz, A.: Mathematical programs with equilibrium constraints: enhanced Fritz John-conditions, new constraint qualifications, and improved exact penalty results. SIAM J. Optim. **20**(5), 2730–2753 (2010). 10.1137/090774975

[55] Kanzow, C., Steck, D., Wachsmuth, D.: An augmented Lagrangian method for optimization problems in Banach spaces. SIAM J. Control. Optim. **56**(1), 272–291 (2018). 10.1137/16M1107103

[56] Klatte, D., Kummer, B.: Constrained minima and Lipschitzian penalties in metric spaces. SIAM J. Optim. **13**(2), 619–633 (2002). 10.1137/S105262340139625X

[57] Kruger, A.Y.: Generalized differentials of nonsmooth functions and necessary conditions for an extremum. Sibirian Math. J. **26**, 370–379 (1985)

[58] Kruger, A.Y., Mehlitz, P.: Optimality conditions, approximate stationarity, and applications-a story beyond Lipschitzness. ESAIM Control Optim. Calc. Var. **28**, 42 (2022). 10.1051/cocv/2022024

[59] Kruger, A.Y., Mordukhovich, B.S.: Extremal points and the Euler equation in nonsmooth optimization problems. Doklady Akademii Nauk BSSR **24**(8), 684–687 (1980)

[60] Levy, A.B.: Implicit multifunction theorems for the sensitivity analysis of variational conditions. Math. Program. **74**, 333–350 (1996). 10.1007/BF02592203

[61] Liang, Y.C., Ye, J.J.: Optimality conditions and exact penalty for mathematical programs with switching constraints. J. Optim. Theory Appl. **190**, 1–31 (2021). 10.1007/s10957-021-01879-y

[62] Luo, Z.Q., Pang, J.S., Ralph, D.: Mathematical Programs with Equilibrium Constraints. Cambridge University Press, Cambridge (1996). 10.1017/CBO9780511983658

[63] Mehlitz, P.: Asymptotic stationarity and regularity for nonsmooth optimization problems. J. Nonsmooth Anal. Optim. 1,6575, (2020) 10.46298/jnsao-2020-6575

[64] Mehlitz, P.: Asymptotic regularity for Lipschitzian nonlinear optimization problems with applications to complementarity constrained and bilevel programming. Optimization **72**(1), 277–320 (2023). 10.1080/02331934.2022.2031190

[65] Mordukhovich, B.S.: Variational Analysis and Generalized Differentiation, Part I: Basic Theory. Applications. Springer, Berlin, Part II (2006). 10.1007/3-540-31247-1

[66] Mordukhovich, B.S.: Variational Analysis and Applications. Springer, Cham (2018). 10.1007/978-3-319-92775-6

[67] Mordukhovich, B.S., Outrata, J.V., Ramírez, C.H.: Second-order variational analysis in conic programming with applications to optimality and stability. SIAM J. Optim. **25**(1), 76–101 (2015). 10.1137/120903221

[68] Outrata, J.V., Kočvara, M., Zowe, J.: Nonsmooth Approach to Optimization Problems with Equilibrium Constraints. Kluwer Academic, Dordrecht (1998). 10.1007/978-1-4757-2825-5

[69] Outrata, J.V., Sun, D.: On the coderivative of the projection operator onto the second-order cone. Set-Valued Anal. **16**, 999–1014 (2008). 10.1007/s11228-008-0092-x

[70] Ramos, A.: Mathematical programs with equilibrium constraints: a sequential optimality condition, new constraint qualifications and algorithmic consequences. Optim. Methods Softw. **36**, 45–81 (2021). 10.1080/10556788.2019.1702661

[71] Rockafellar, R.T., Wets, R.J.B.: Variational Analysis, Grundlehren der mathematischen Wissenschaften, vol. 317. Springer, Berlin (1998). 10.1007/978-3-642-02431-3

[72] Shapiro, A.: On concepts of directional differentiability. J. Optim. Theory Appl. **66**, 477–478 (1990). 10.1007/BF00940933

[73] Sun, D., Sun, J.: Semismooth matrix-valued functions. Math. Oper. Res. **27**(1), 150–169 (2002). 10.1287/moor.27.1.150.342

[74] Tret’ Yakov, A.A.: Necessary and sufficient conditions for optimality of -th order. USSR Comput. Math. Math. Phys. **24**(1), 123–127 (1984). 10.1016/0041-5553(84)90132-0

[75] Wu, J., Zhang, L., Zhang, Y.: Mathematical programs with semidefinite cone complementarity constraints: constraint qualifications and optimality conditions. Set-Valued Var. Anal. **22**, 155–187 (2014). 10.1007/s11228-013-0242-7

[76] Ye, J.J., Ye, X.Y.: Necessary optimality conditions for optimization problems with variational inequality constraints. Math. Oper. Res. **22**(4), 977–997 (1997). 10.1287/moor.22.4.977

